# *MonteCarbo*: A software to generate and dock multifunctionalized ring molecules

**DOI:** 10.1002/jcc.26559

**Published:** 2021-05-13

**Authors:** Santiago Alonso‐Gil

**Affiliations:** ^1^ Department of Structural and Computational Biology, Max F. Perutz Laboratories University of Vienna Vienna Austria

**Keywords:** carbohydrates, conformations, docking, drug design, Monte Carlo

## Abstract

MonteCarbo is an open‐source software to construct simple 5‐, 6‐, and 7‐membered ring multifunctionalized monosaccharides and nucleobases and dock them into the active site of carbohydrate‐active enzymes. The core bash script executes simple orders to generate the *Z*‐matrix of the neutral molecule of interest. After that, a Fortran90 code based on a pseudo‐random number generator (Monte Carlo method) is executed to assign dihedral angles to the different rotamers present in the structure (ring and rotating functional groups). The program also has a generalized internal coordinates (GIC) implementation of the Cremer and Pople puckering coordinates ring. Once the structures are generated and optimized, a second code is ready to execute in serial the docking of multiple conformers in the active site of a wide family of enzymes.

## Graphical abstract

*MonteCarbo* is a computational tool that may help in the research of medical drug design. Experiments and computation combine their power to improve the strategies to find new solutions for carbohydrate‐related illnesses. The synthesis of new compounds, derivatives of natural sugars, evolves continuously and theory may be the key to optimize such chemical processes with standard easy‐to‐use interfaces. *MonteCarbo* will become a powerful ally in carbohydrate research.

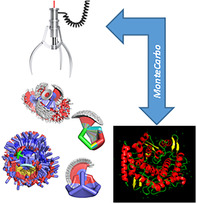

## 1 INTRODUCTION

The symbiosis between experiments and computation is key, in the XXI century, for the development of new substrates and inhibitors in enzymology.^1^, ^2^, ^3^, ^4^ In glycobiology, understanding the catalytic mechanism, how the natural monosaccharides change their shape in the active site of the enzymes, is crucial to develop and design new drugs for glyco‐related illnesses.^5^ Acarbose and miglitol for type 2 diabetes^6^ and swainsonine for cancer treatment^7^ are good examples of drugs acting as glycosidase inhibitors. In general, the most extensively used molecules for this purpose are derivatives of piperidine (*N*‐based rings), thiane (S‐based rings), and sulfolane (SO_2_‐based rings).^8^, ^9^, ^10^, ^11^

Apart from medical purposes, the design of new in silico monosaccharides is a fundamental step to predict the reaction mechanism of carbohydrate‐active enzymes. First, sulfur‐based derivatives usually mimic natural substrates' shapes for glycosidases, showing the conformation of the Michaelis complex of the catalyzed reaction.^12^, ^13^ Second, gluco‐, manno‐, and galacto‐imidazole are used as transition state mimics for glycoside hydrolases, showing the conformation of the most energetic state along the hydrolysis.^14^ Finally, fluoro‐derivative monosaccharides are used to activate the glycosyl‐enzyme intermediate (or covalent intermediate) formation in retaining glycosidases. This information is key to decipher the conformation of the sugar moiety after the glycosylation step of the reaction.^15^, ^16^ However, experiments show that thio‐sugars do not adopt the same conformations as the natural ligand.^17^, ^18^, ^19^ In these cases, where the conformation of the mimic will not be the same as the natural ligand, it can be advantageous to quickly produce a large number of conformations for consideration.

**FIGURE 1:**
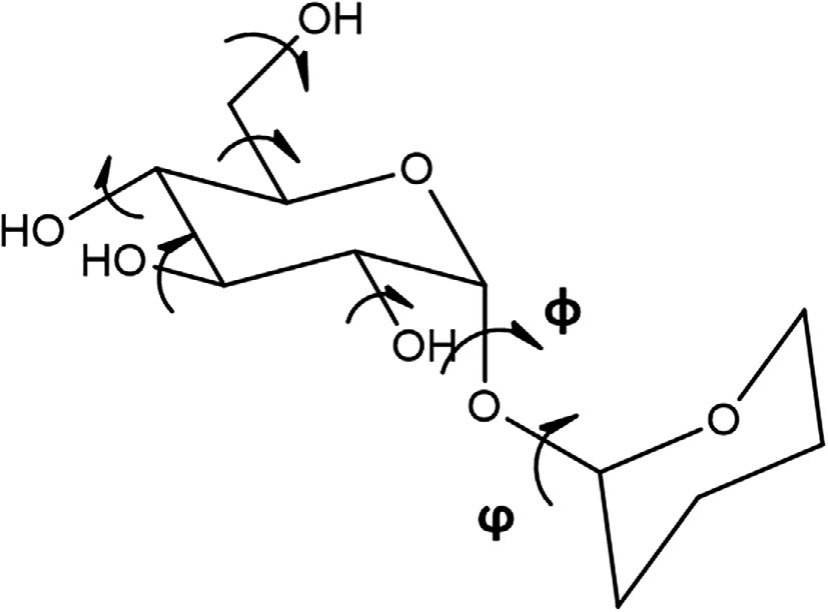
Simplified representation of a disaccharide and the rotamers present on it

Following the prescription reported by French and Brady in 1990,^20^ the main limitations connected with proper modeling of carbohydrates are:

- *Hydrogen bonding*: saccharides are polyalcohol molecules. The oxygen atoms present in the ring and OH groups are H‐bond acceptors for the alcohol groups' hydrogen atoms. A proper description of the H‐bond interactions is determinant for the reliability of the models.
- *Anomeric effects*: carbohydrates present a particular sensitivity in the asymmetric centers where a carbon atom is connected to two electronegative oxygen atoms. Depending on the medium (solvent or enzymatic environment), the bond length of the glycosidic bond, C—O bonds and torsions in the vicinity of the anomeric carbon are altered depending on the ring's conformation and several interactions between the sugar and the medium.
- *Multiple minimum problems*: during the geometry and energy minimization of our model (using a force field or quantum mechanics), the optimization will show a local minimum. This structure could not be a global minimum due to the many torsional degrees of freedom of a simple monosaccharide.
- *Comparison with experiments*: the matching between a single model and experiments with carbohydrates is not always possible. Monosaccharides present one or more conformations in solution that differ in their intramolecular interactions and may affect the results of, for instance, an NMR spectra.
- *Conformational analysis*: natural carbohydrates are formed by 5‐ and 6‐membered rings. This kind of structure presents a complex conformational free energy surface. Twenty different canonical conformations can describe Furanose‐like systems (10 envelops—E—and 10 twisted—T—conformers) and 38 canonical conformations represent pyranose‐like structures (2 chairs—C—, 6 boats—B—, 6 skew‐boats—S—, 12 envelops—E—, and 12 half‐chairs—H).^21^ Furthermore, every rotamer (alcohol group, CH_2_OH, *φ*, and *ϕ* dihedral angle in oligosaccharides—Figure 1) and its interaction with other rotamers can affect the model's stability pattern.

Improvements in computational capabilities since the publication of the French and Brady article have turned some of the limitations described in the article into simple problems solved in a matter of minutes. A recently published review by Scherbinina and Toukach shows state‐of‐the‐art carbohydrates structure databases and computational techniques to obtain reliable models.^22^ Also, French and Johnson reported a review about the most insightful works in the matter of modeling carbohydrates.^23^

In the present work, we want to focus on applying Monte Carlo (MC) techniques^24^ in the conformational study of carbohydrates. This method's primary basis is to generate random changes in a saccharide structure to search a possible conformation with lower energy and repeat this procedure hundreds of thousands or millions times until ensuring a reliable sampling of the molecule's conformational space. To show some examples, in 1993, Peters and coworkers applied this technique for the conformational analysis of four disaccharides focusing on the random sampling of the exocyclic dihedral angles (CH_2_OH, *φ*, and *ϕ* – Figure 1),^25^ Dowd et al. in 2011 also add the OH groups to the Peters approach to study the opened‐ and closed‐ring forms of carbohydrates^26^ and in 2017, Zhang and collaborators combined MC and torsion‐angle molecular dynamics simulations for oligo‐ and polysaccharides.^27^

Many computational approaches were used to construct conformational free and potential energy surfaces for ring molecules.^28^, ^29^, ^30^, ^31^ In this article, we present an MC‐based code called *MonteCarbo*. Its principal function is to generate conformers of multi‐functionalized 5‐, 6‐, or 7‐membered ring molecules. Afterward, the program can perform docking calculations with them into the active site of several glycosidases to test their substrate/inhibitor mimic capabilities. While the previous MC‐based studies addressed only the exocyclic dihedral angles, our approach increased the versatility of such methods to include the ring's puckering as a random variable. With this cheap‐and‐fast approximation, we firmly believe that MonteCarbo will become a powerful tool in the field of drug design.

## 2 METHODS

### 2.1 Theoretical background

#### 2.1.1 Cremer and Pople puckering coordinates

In 1975, Cremer and Pople developed a mathematical expression to describe the conformational space of *N*‐membered rings.^32^ Given a ring structure with *N* atoms, *N* − 3 puckering coordinates are needed to describe the whole conformational space. For cases where *N* is odd, the expressions for the puckering amplitude(s) *q*_m_ and phase angle(s) *ϕ*_m_ are defined as:

(1) $$q_{m}\cdot \cos\varphi_{m}={\left(\frac{2}{N}\right)}^{\frac{1}{2}}\sum_{j=1}^{N}z_{j}\cdot \cos\left(\frac{2\mathrm{\pi m}\left(j-1\right)}{N}\right)=B_{m}$$

(2) $$q_{m}\cdot \sin\varphi_{m}=-{\left(\frac{2}{N}\right)}^{\frac{1}{2}}\sum_{j=1}^{N}z_{j}\cdot \sin\left(\frac{2\mathrm{\pi m}\left(j-1\right)}{N}\right)=A_{m}$$

Applying for m = 2, 3…, (*N* − 1)/2. For systems with an even *N*, the last puckering coordinate is defined as:

(3) $$q_{\frac{N}{2}}=N^{-\frac{1}{2}}\sum_{j=1}^{N}{\left(-1\right)}^{j-1}\cdot z_{j}$$

where *z*_*j*_ are the normalized coordinates of the *N* atoms of the ring using their geometrical center as the origin of coordinates.

**FIGURE 2:**
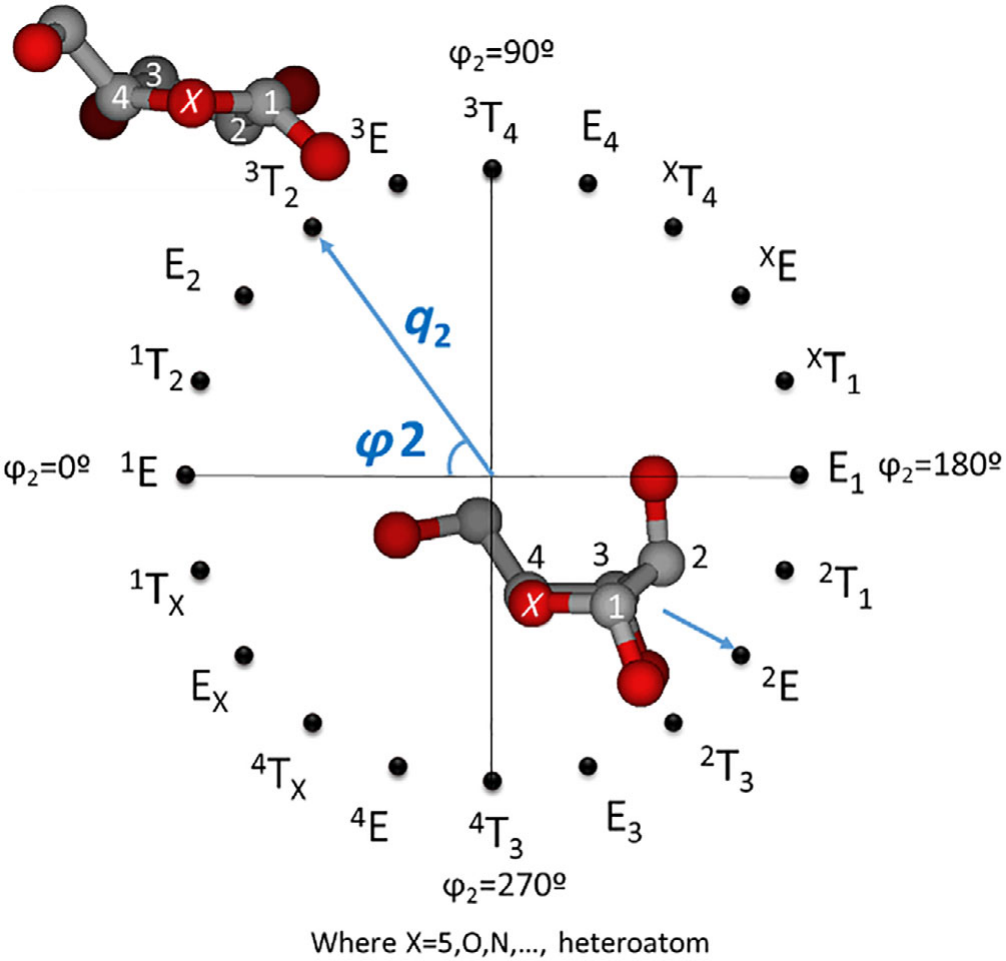
Conformational space for five‐membered rings

**FIGURE 3:**
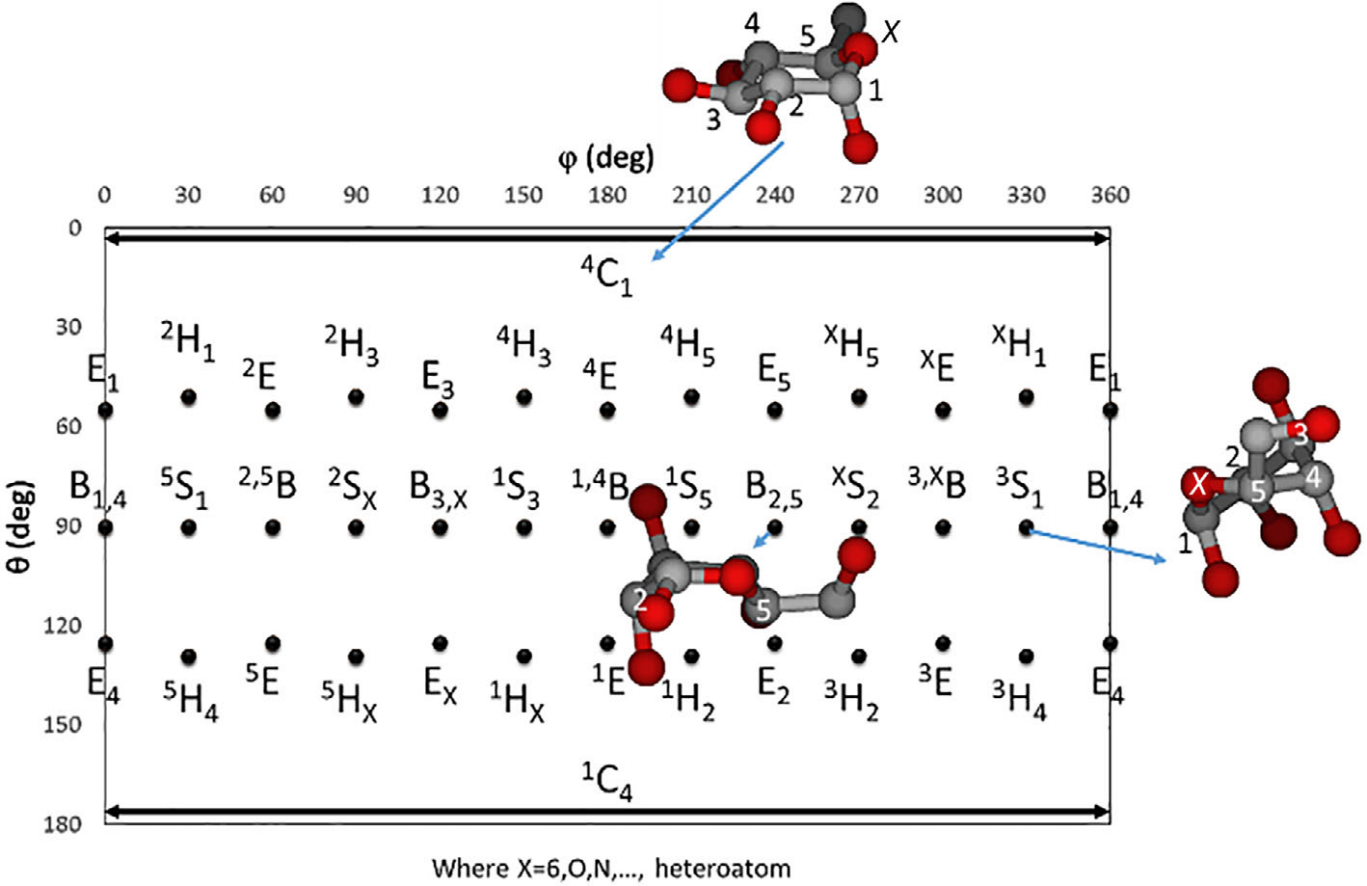
Mercator representation of the conformational space for six‐membered rings

**FIGURE 4:**
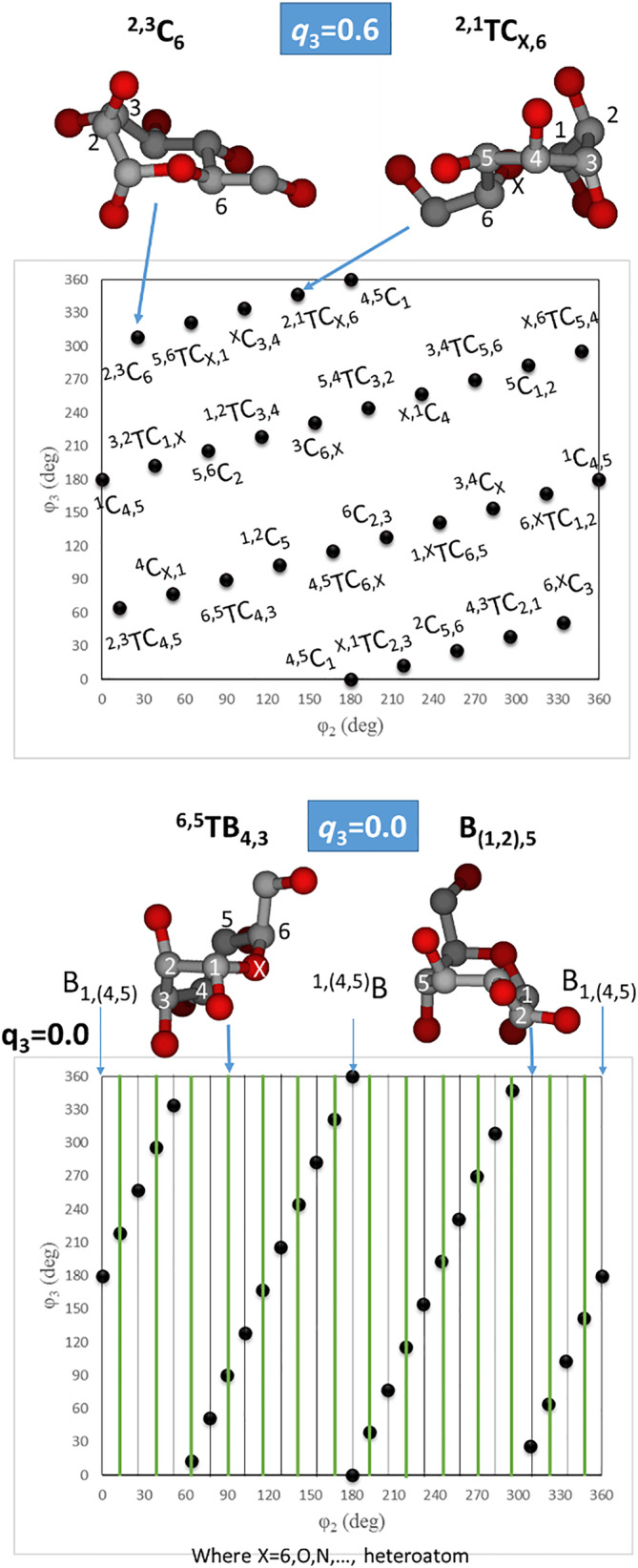
Conformational subspaces for seven‐membered rings at *q*3 = 0.6 (up) and *q*3 = 0.0 (down)

For a consistent description of the Cremer and Pople coordinates, the ring members are tagged from 1 to *N*. In this work, the anomeric carbon is indicated by the number 1 and the heteroatom by the number *N* (*X* in Figures 2, 3, 4). The rest of the members have to be connected consecutively. In case that the ring has no heteroatom, the selection of the first and last atoms will depend on the user's choice.

The conformational space of a 5‐membered ring formed by 20 canonical conformers is described by q_2_ and *ϕ*_2_ (Figure 2). Envelops—E—have four atoms on‐the‐plane and one only atom above or below the plane. Twists—T—have three coplanar atoms and two consecutive atoms on opposite sides of the plane.^32^The three‐dimensional (3‐D) conformational space of 6‐membered rings is defined by *q*_2_, *q*_3_ and *ϕ*_2_. However, the scientific community uses the *Q*, *ϕ*, and *θ* polar coordinates and project them into a Mercator representation (Figure 3). More details about the terminology and symbolism of the different 38 conformers are already described by IUPAC in reference 33.The complex (*q*_2_, *q*_3_, *ϕ*_2_, *ϕ*_3_) 4‐Dimensional conformational space for 7‐membered rings can be simplified to a 3‐D representation and divided into three (*ϕ*_2_, *ϕ*_3_) planes at *q*_3_ = 0.6 (Twist‐Chair/Chair plane), *q*_3_ = 0.0 (Twist‐Boat/Boat plane) and *q*_3_ = 0.4 (Sofa/Twist‐Sofa/Sofa‐Boat).^34^ For clarity and practical reasons, the *q*_3_ = 0.4 plane is not depicted in Figure 4.

It is worth mentioning that the *q*_3_ = 0 plane presents a harp distribution (Figure 4, *down*) where the twist‐boat (TB)—green strings—and the boat (B)—gray strings—conformations are described by a given value of *ϕ*_2_ while a change in *ϕ*_3_ does not affect to the structure (more details in Supporting Information).

In case the bond distances and angles are known, *N* − 3 endocyclic dihedral angles are necessary to construct and define a specific conformation for an *N*‐membered ring. In this article's following point, this geometrical property will be used to establish a random selection of conformers.

### 2.2 Simulation algorithm

#### 2.2.1 GICs‐based puckering code for Gaussian 16

The main idea of this work is to develop an algorithm to pick a random conformation from a group of structures. This follows a three‐stepped pathway: generating the conformers by changing their puckering coordinates, creating a database with the endocyclic structural information, and selecting randomly the array of endocyclic dihedral angles corresponding to a unique conformation. For the first step, we present a strategy based on performing scan calculations with the Gaussian 16 software.^35^ The main initial problem was that Cremer and Pople's mathematical expressions are not implemented as generalized internal coordinates (GICs) in the quantum mechanics code. However, the last version of Gaussian includes adding and defining homemade GICs using the most common mathematical operators.

Following the recipe described in reference 31 and starting from the x, y and z (Cartesian) coordinates of the *N* atoms of the ring, the code calculates the center of geometry (XCntr, YCntr, and ZCntr functions). It recalculates the *N* atoms' new coordinates using the center of geometry as the origin of coordinates. After that, employing simple mathematical operators and the function *SQRT*, the code obtains the values of *A*_*m*_, *B*_*m*_, and *q*_*N*/2_ (for *N* = 6) defined by Equations ((1), (2), (3)).

At the time to get the values of q_m_ and *ϕ*_m_ for *N* = 5 and *N* = 7 and *Q*, *ϕ*, and *θ* for *N* = 6, one technical problem appeared: Gaussian does not have the function *arctan* defined in its code and its necessary to use it to get the puckering phases:

(4) $$q_{m}=\sqrt{{A_{m}}^{2}+{B_{m}}^{2}}$$

(5) $$\varphi_{m}=\arctan\left(\frac{A_{m}}{B_{m}}\right)$$

By a trigonometric relationship between the *arctan* and the *arccos* functions, we can transform the Equation (5) into:

(6) $$\varphi_{m}=\arctan\left(\frac{A_{m}}{B_{m}}\right)=\arccos\left(\frac{B_{m}}{\sqrt{{A_{m}}^{2}+{B_{m}}^{2}}}\right)$$

However, this conversion only defines the interval [0,*π*] of *ϕ*_m_. To solve this problem, we observed that the function A_m_ is antisymmetrical at *ϕ*_m_ = 0. So, to define *ϕ*_m_ in the interval [0,2*π*], we use the following expression:

(7) $$\varphi_{m}=\frac{A_{m}}{\sqrt{{A_{m}}^{2}}+\varepsilon }\arccos\left(\frac{B_{m}}{\sqrt{{A_{m}}^{2}+{B_{m}}^{2}}}\right)+180$$

where *ε* = 10^−6^ avoids a division by zero when *A*_*m*_ = 0.

In the case of *N* = 6, the polar coordinates are calculated as follows:

(8) $$Q=\sqrt{q_{2}^{2}+q_{3}^{2}}>0$$

(9) $$\varphi_{m}=\frac{A_{m}}{\sqrt{{A_{m}}^{2}}+\varepsilon }\arccos\left(\frac{B_{m}}{q_{2}}\right)+180\in \left[0,2\pi \right]$$

(10) $$\theta =\arccos\left(\frac{q_{3}}{\sqrt{q_{2}^{2}+q_{3}^{2}}}\right)\in \left[0,\pi \right]$$

One of the limitations of the mathematical interface of Gaussian to define GICs is the absence of periodicity. For this reason, the phase puckering coordinates present problems when their values are close to 0 or 2*π*. Although we could able to explore around 99% of the conformational potential energy surfaces of 5‐, 6‐, and 7‐membered rings. The reader can find these codes in the files *puckN.gic* (for *N* = 5, 6, and 7) of the *MonteCarbo* distribution.

#### 2.2.2 Quantum‐mechanical calculations: obtaining the structural parameters for MonteCarbo libraries

All the quantum‐mechanics calculations were performed with Gaussian 16^35^ employing Density Functional Theory.^36^ By ensuring a proper equilibrium between a good H‐bond description, accuracy, and time‐economy,^37^ the calculations were performed with the B97‐2 functional^38^ and the def2‐SVP basis set.^39^ Scan optimizations were performed using the *tight* criteria for convergence.

For collecting distances, angles and, more importantly, endocyclic dihedral angles, scan calculations of saturated and nonsubstituted 5‐, 6‐, and 7‐membered rings were performed using the previously GICs‐based implementation of the puckering coordinates. The chosen molecules had the C_*N*_H_2*N*_X formula where *N* = 5, 6, and 7 and *X* = CH_2_, O, S, SO_2_, NH, BH, and PH (Figure 5, more details in Figures S1–S21).The evolution of the endocyclic dihedral angles is available in the *N‐x‐H‐y‐X‐D#.txt* files of the *MonteCarbo* distribution (*x* = 5, 6, 7; *y* = 0, 1; *X* = Oxy, S, SO2, N, B, P, and # = 1…, *N* − 3). For instance, the cyclopentane information is in the *N‐5‐H‐0‐D1.txt* and *N‐5‐H‐0‐D2.txt* files.

**FIGURE 5:**
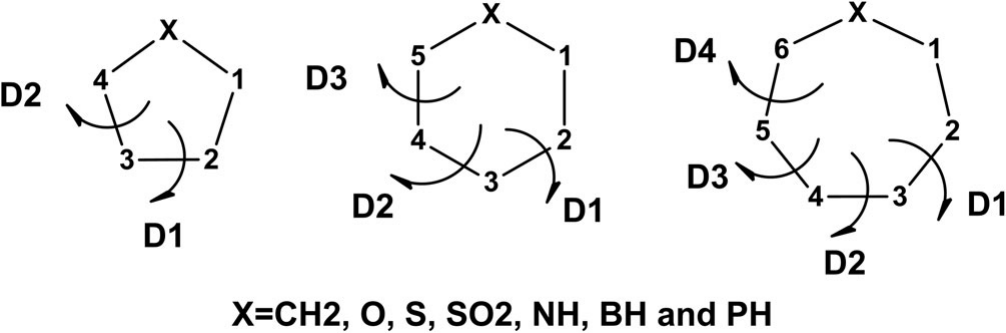
Schematic representation of the studied rings and the definition of the main endocyclic dihedral angles *D*#, where # = 1…, *N* − 3

Also, for the proper construction of functional groups attached to the rings, several molecular models formed by a ring and the substitute groups were optimized (list of available functional groups and structural details in Supporting Information).

The distances, angles, and exocyclic dihedral angles are available in the files with extension *.var of the *MonteCarbo* distribution.

#### 2.2.3 Random number generator

*MonteCarbo* generates a z‐matrix Gaussian input with the structural information of the molecule of study. However, there are at least *N* − 3 endocyclic dihedral angles that will change from one structure to another. If the molecule contains rotamers like —OH, —CH_2_OH, and so on, the number of random degrees of freedom increases.

Inspired by Vilaseca and coworkers' work,^40^ we developed a *Fortran 90* code based on a pseudo‐random number generator. This code generates *N* − 3 pseudo‐random numbers that take *N* − 3 endocyclic dihedral angles. After that, it generates one pseudo‐random number in the range of [−180°, 180°] per rotamer. Finally, the *MonteCarbo* script implements the given dihedral angles into the file where the variables of the z‐matrix are defined. In addition to that, for rings with NH and PH, the equatorial/axial position of the H‐N/H‐P hydrogen requires an extra random number that can be −120° or 120°.

The rangen*.f90 files are available and open‐source, ready for changes, and corrections in the *MonteCarbo* folder.

#### 2.2.4 MonteCarbo script

**FIGURE 6:**
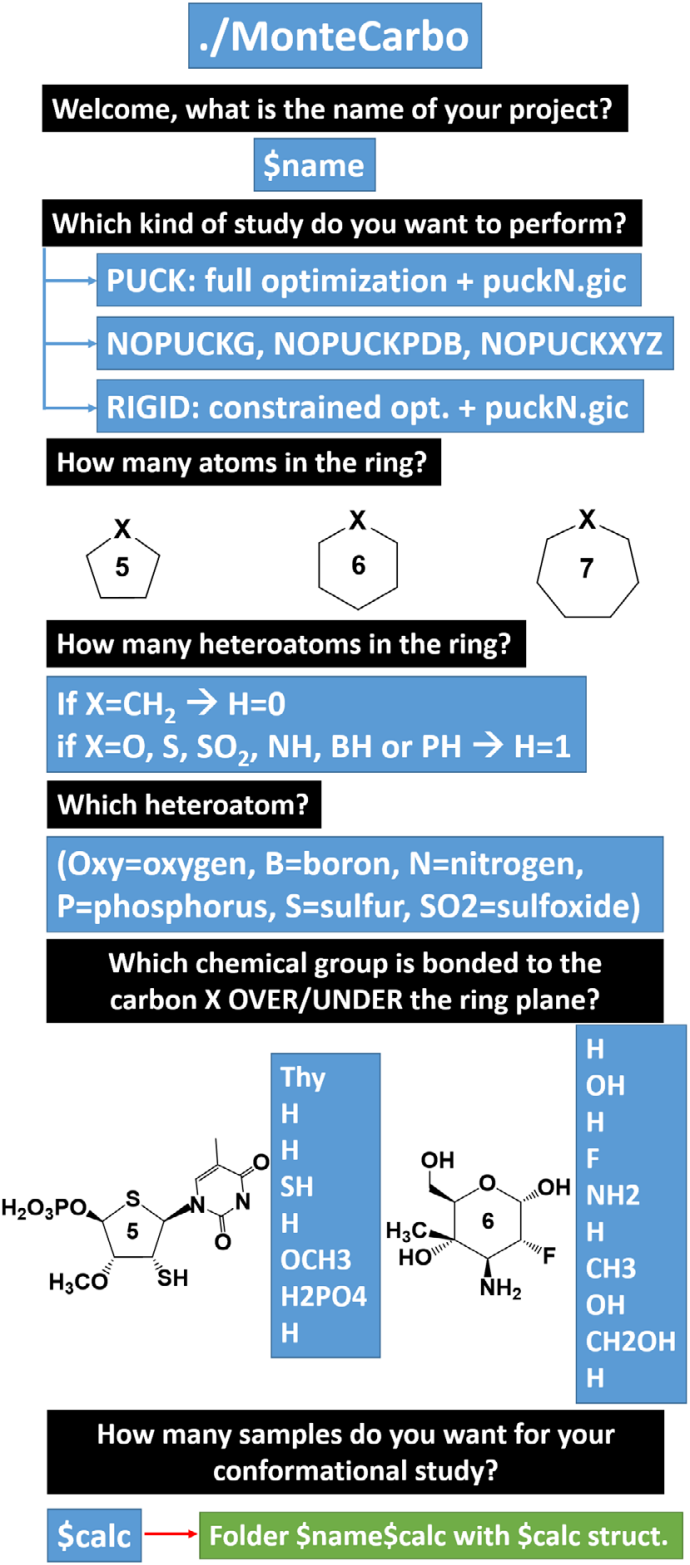
Workflow for the execution of the *MonteCarbo* script. The authors strongly recommend using an *input.dat* file to perform parallel jobs and increase the projects' efficiency (more details in the README file). Depending on the user's will, MonteCarbo will generate *$calc* Gaussian *Z*‐matrix input, *$calc* PDB or *$calc* XYZ coordinates files (*$calc* is the number of conformers requested by the user)

*MonteCarbo* is a bash script that generates a z‐matrix Gaussian input model of a neutral, multi‐functionalized (5, 6, or 7)‐membered ring molecule. The script requires some information as an input to construct the model and the random replicas (Figure 6).

The script combines the information and power of the *N‐x‐H‐y‐X‐D#.txt*, rangen*.f90 and *puckN.gic* files to develop a *calc.gjf* input file. Depending on the user's selection, the final output of the required structures can be a Gaussian input, a PDB or an *XYZ* file (Open Babel^41^ is required for the conversions).

The main limitation of *MonteCarbo* is that in extreme multi‐functionalization cases with voluminous groups, the code generates structures with steric hindrance or overlapping. For instance, in case our molecule presents two neighbor CH_2_OH groups, the generation of random conformers of it will lead into some structures where the OH groups overlap or cross the same point in the space.

The code is free to download in https://github.com/drsalonsogil/montecarbo and a *README* file is available with further information.

#### 2.2.5 MCdock: testing the substrate/inhibitor role of the monosaccharide in glycosidases

**FIGURE 7:**
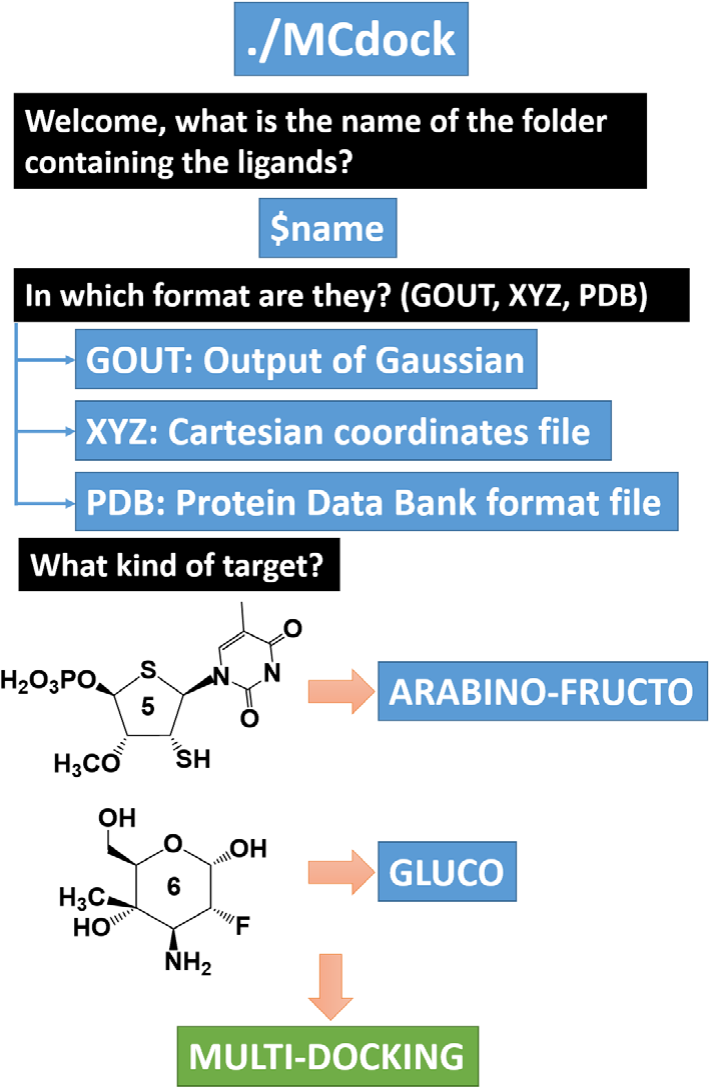
Workflow for the execution of the *MCdock* script (more details in the *README* file). This process' output(s) will be the output obtained by the multiple AutoDock Vina calculations^43^

*MCdock* is another bash script that prepares the generated and/or optimized structures^1^ by *MonteCarbo* to be docked in the active site of a wide group of glycoside hydrolases. For that purpose, the program requires the execution of the *prepare_ligand* code of AutoDock 4.2. package^42^ (present in MGLTools) and the *vina* code of the AutoDock Vina package.^43^ The workflow of *MCdock* is shown in Figure 7.The repository contains a *receptors* folder where several families of glycosidases are classified depending on the name of the sugar that hydrolyses: ARABINO‐FRUCTO, FUCO, GALACTO, GALNAC‐GLCNAC, GLUCO, IDURONIC‐SIALIC, MANNO, RHAMNO, and XYLO. The user is free to create new folders and generate the *pdbqt* and configuration files for the receptor of interest. Otherwise, AutoDock Vina will not find a receptor or the coordinates where the ligand has to be docked.

## 3 APPLICATIONS

Following the work‐flows of Figures 6 and 7, we have executed *MonteCarbo* in the generation of three ring molecules: 2‐hydroxy‐tetrahydrofurane, *α*‐d‐glucose and *α*‐d‐glycero‐d‐idoseptanoside. In the last example, after an initial geometrical optimization, we have also applied *MCdock* to analyze the binding properties between the 7‐membered ring sugar and mannosidases (details about input files and geometries are on pages S88–S100 of Figures S22–S26).

### 3.1 Simple case: 2‐hydroxy‐tetrahydrofurane

**FIGURE 8:**
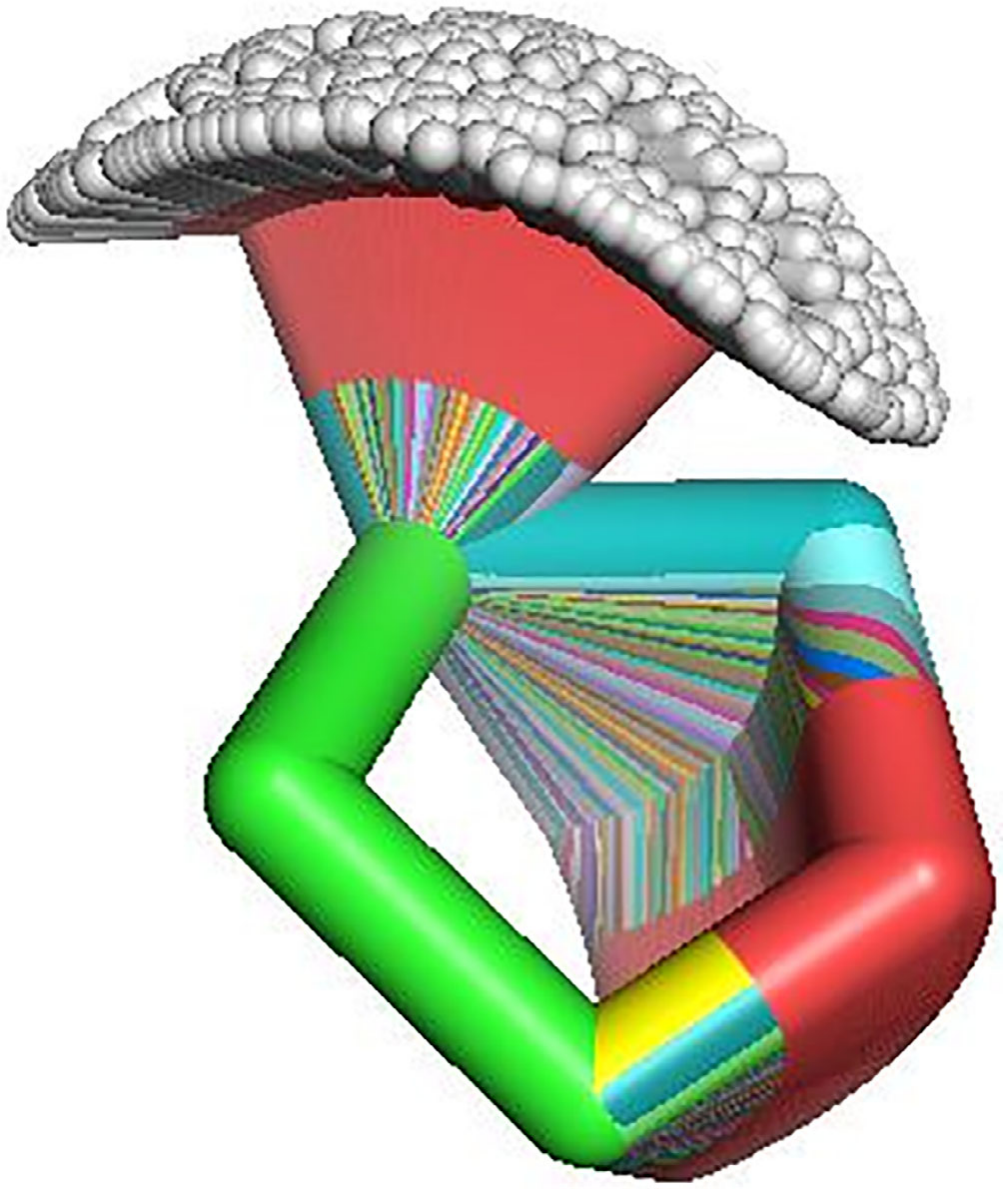
Overlap of 500 structures of the 2‐hydroxy‐tetrahydrofurane molecule generated by *MonteCarbo*

After generating 500 conformers of 2‐OH‐C_4_H_7_O using *MonteCarbo* and representing the overlap between the different obtained structures with PyMOL^2^ the result is shown in Figure 8.Using a simple case, we can easily observe how the code chooses between different conformers of a 5‐membered ring and the different orientations of the hydrogen atom of the hydroxyl group present in the molecule. Furthermore, due to the conversion from *z*‐matrix to Cartesian coordinates, the structure's first carbon is always in the origin of coordinates. The second atom is still at the same distance (and at the same position). The other ring members form a continuous rainbow due to the proximity between the structures over the conformational energy surface.

### 3.2 Test case: *α*‐d‐glucose

**FIGURE 9:**
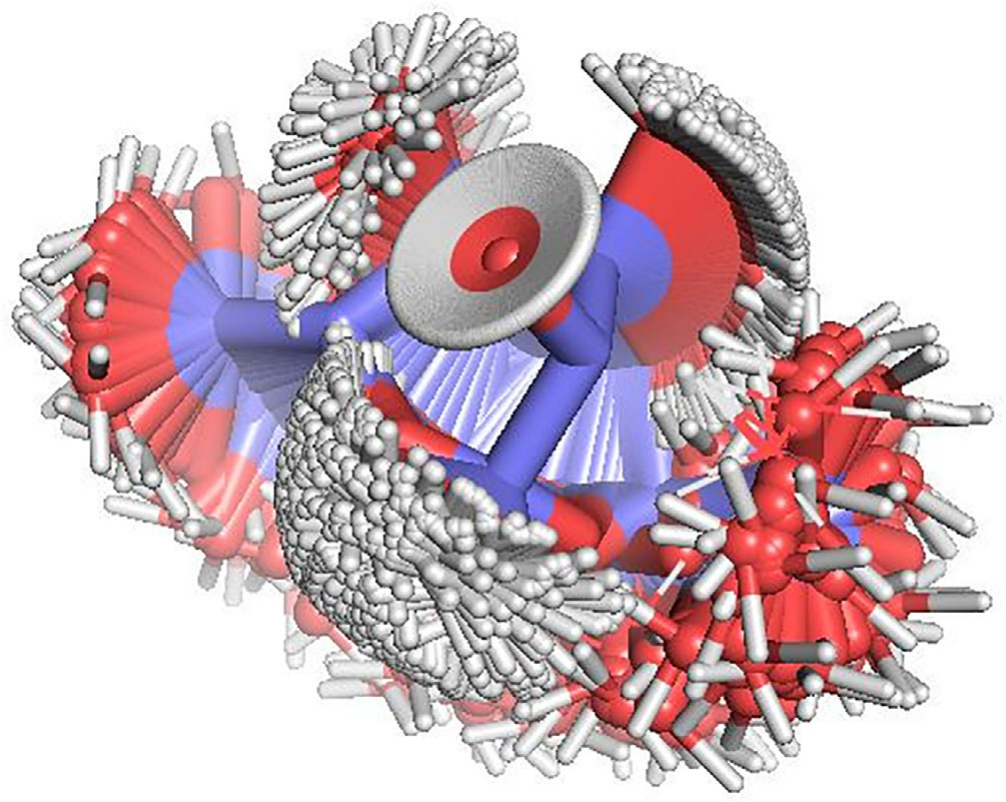
Overlap of 500 structures of *α*‐d‐glucose generated by *MonteCarbo*

As performed with the previous simple case, 500 conformers of *α*‐d‐glucose were generated executing *MonteCarbo* and the resulting overlap is represented in Figure 9.Compared with the previous structure, we observe a hydroxyl group whose oxygen remains in the same position in the center of the image. Its hydrogen position takes a continuous of positions due to the random assignation of the H—O—C—C dihedral angle between −180° and 180° by the pseudo‐random number generator.

### 3.3 Docking: a 7‐membered ring mimics mannose

Inspired by the work of Peczuh, Ernst et al. where a crystal structure of mannose‐specific bacterial lectin FimH in complex with a septanoside is reported,^44^ 50 structures of *α*‐d‐glycero‐d‐idoseptanoside were generated and minimized (B97‐2/def2‐SVP level) in the gas‐phase, using tight criteria. Frequencies were calculated to ensure the structures were located in minima over the conformational energy surface.

In the experiment with the septanoside, a ^3,4^TC_5,6_ conformation is observed(PDB 5CGB), while the experiment with mannose showed a ^4^C_1_ conformation (PDB 4BUQ).^45^ Furthermore, a computational analysis of the 1‐hydroxymethyl‐α‐D‐glycero‐D‐idoseptanoside shows the ^3,4^TC_5,6_ conformation as the most stable conformation.^46^ Also, after analyzing the 50 structures of the hydrolyzed *α*‐d‐glycero‐d‐idoseptanoside, the most stable conformation is the ^3,4^TC_5,6_ (more details in Supporting Information). Then, we can conclude that the FimH enzyme recognizes the sugar in the most stable conformation without changing its shape. But what happens if we try to dock the septanoside in a mannosidase?

**FIGURE 10:**
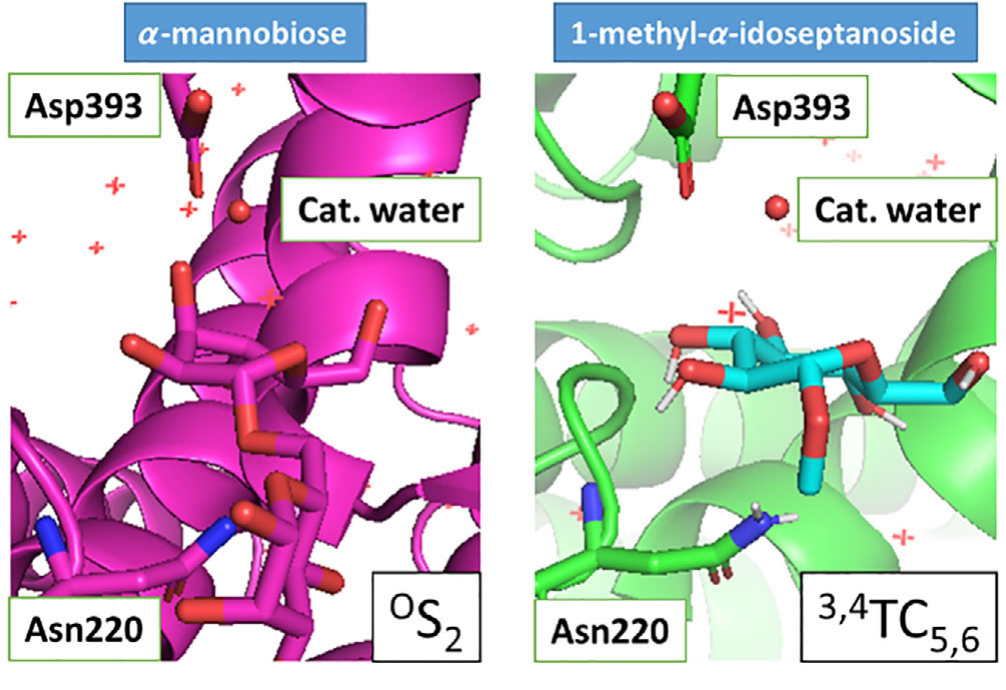
Representation of the active site of the GH125 *α*‐mannosidase in presence of the natural substrate (*α*‐mannobiose, PDB 5M7I18) and 1‐methyl‐*α*‐d‐glycerol‐d‐idoseptanoside (docking calculation)

Using *MCdock*, we docked a ^3,4^TC_5,6_ structure of hydrolyzed *α*‐d‐glycerol‐d‐idoseptanoside, 1‐methyl‐*α*‐d‐glycerol‐d‐idoseptanoside, and dimethyl‐1,*X*‐*α*‐d‐glycerol‐d‐idoseptanoside, for *X* = 2, 3, 4, 5, and 7, in the active site of nine mannosidases (six *α*‐ and three *β*‐mannosidases). The OCH_3_ groups were included to avoid nonexisting H‐bond interactions in presence of an oligosaccharide. In Figure 10, comparing the structure of the GH125 glycosidase in the presence of the natural substrate and the septanoside indicates the mimic power of the artificial sugar. It correlates the ^O^S_2_ conformation of the 6‐membered rings with the ^3,4^TC_5,6_ of the 7‐membered rings.The full report about the docking calculations is available in Supporting Information. Those calculations found four suitable GH‐septanoside complexes where the 7‐membered ring in a ^3,4^TC_5,6_ conformation mimics the 6‐membered ring of the natural substrates in an ^O^S_2_ conformation (GH38, GH76, GH92, and GH125, Figure S27).

## 4 CONCLUSIONS

*MonteCarbo* is an easy‐to‐use computation‐friendly software able to model and to dock multi‐functionalized monosaccharides. Being an open‐source package, the code‐user interaction is permitted, and, depending on the circumstances of the research, *MonteCarbo* and *MCdock* can change and evolve in the proper direction.

We have demonstrated the power of the provided codes in terms of quick‐and‐cheap structure generation and the relevance of the obtained results testing new substrates and inhibitors for carbohydrate‐active enzymes.

As a limitation, the program does not have any internal mechanism to decipher if a structure will be physically reliable or to avoid chemical changes during the optimization process. These processes require a postanalysis to confirm and delete incorrect configurations.

## Supporting information

**Appendix** S1: Supporting Information

## Endnotes

^1^
The scientific community strongly recommend that docking calculations are performed with optimized ligands. *MonteCarbo* has the RIGID option keeping the conformation fixed during the optimization, in case the conformation of interest is not a minimum in the conformational potential energy surface.

^2^
The PyMOL Molecular Graphics System, Version 1.2r3pre, Schrödinger, LLC.
