# Supplementary material for: MonteCarbo: A software to generate and dock multifunctionalized ring molecules
Source: J Comput Chem. 2021 May 13;42(21):1526–34. doi: 10.1002/jcc.26559 (PMC8359999; doi:10.1002/jcc.26559)
Supplement: Supplementary file 1 — Appendix S1: Supporting Information [file JCC-42-1526-s001.pdf]

SUPPORTING INFORMATION

MonteCarbo: a software to generate and dock multifunctionalized ring molecules

Santiago Alonso-Gil[1]

[1] Department of Structural and Computational Biology, Max F. Perutz Laboratories, University of Vienna, Dr.-Bohr-Gasse 9, Wien, Austria, 1030

*Corresponding author: santiago.alonso.gil@univie.ac.at

Supporting Information Contents

Conformational behavior of 7-membered rings p. S3

Scan calculations to obtain the endocyclic dihedral angles D# p. S4

List of available functional groups in *MonteCarbo* p. S85

Applications p. S88

Simple case: 2-hydroxy-tetrahydrofurane p. S88

Test case: α-D-glucose p. S89

Docking: a 7-membered ring mimics mannose p. S90

Conformational behavior of 7-membered rings

Details about the TB/B plane

Interconversion TB → B

The TB/B plane (q_3→0) is a complex subspace of the conformational landscape of a 7-membered ring. On the contrary as it happens in the TC/C plane, the conformational change occurs when φ_2 is activated. Furthermore, fixing φ_2 and changing φ_3 results in the same conformation (with subtle differences in the structure). For these reason, in Figure 4, we present the TB/B plane as an harp distribution (where the whole string represents slightly different structures with the same shape). For sake of clarity, the authors decide not to present the names of all the conformations in Figure 4. Here, we present the interconversion pathway between twist boats and boats.

(φ_2=0°) $B_{1,(4,5)}$ ←→ $^{2,3}TB_{4,5}$ ←→ $^{(2,3),6}B$ ←→ $^{3,2}TB_{1,X}$ ←→ $B_{(X,1),4}$ ←→ $^{5,6}TB_{X,1}$ ←→ $^{2,(5,6)}B$ ←→ $^{6,5}TB_{4,3}$ ←→ $B_{(3,4),X}$ ←→ $^{1,2}TB_{3,4}$ ←→ $^{(1,2),5}B$ ←→ $^{2,1}TB_{X,6}$ ←→ $B_{3,(6,X)}$ ←→ $^{4,5}TB_{6,X}$ ←→(φ_2=180°) $^{1,(4,5)}B$ ←→ $^{5,4}TB_{3,2}$ ←→ $B_{(2,3),6}$ ←→ $^{X,1}TB_{2,3}$ ←→ $^{(X,1),4}B$ ←→ $^{1,X}TB_{6,5}$ ←→ $B_{2,(5,6)}$ ←→ $^{3,4}TB_{5,6}$ ←→ $^{(3,4),X}B$ ←→ $^{4,3}TB_{2,1}$ ←→ $B_{(1,2),5}$ ←→ $^{6,X}TB_{1,2}$ ←→ $^{3,(6,X)}B$ ←→ $^{X,6}TB_{5,4}$ ←→ (φ_2=360°) $B_{1,(4,5)}$

Scan calculations to obtain the endocyclic dihedral angles D#

5-membered rings

C_5H_{10}

#p opt=(modredundant,tight) b972/def2svp nosymm maxdisk=8GB geom=GIC scf=xqc

SAM opt

0 1

C	0.04395400	0.21575900	0.03500800
C	0.01998900	-0.28395900	1.47571800
C	1.38668700	0.18481900	1.99403600
C	2.22058400	0.35130700	0.70252800
C	1.37381600	0.02727900	-0.38063300
H	-0.21636687	1.25284064	-0.00492588
H	-0.62225705	-0.36139656	-0.57158437
H	-0.05536858	-1.35079583	1.50858776
H	-0.77483766	0.17489339	2.02581350
H	1.82253013	-0.55592893	2.63140031
H	1.29511300	1.11945971	2.50683317
H	3.05954622	-0.31253364	0.72144885
H	2.55706406	1.36307161	0.61300957
H	1.58746016	0.66759477	-1.21084495
H	1.52306314	-0.99380634	-0.66349343

XC(inactive)=XCntr(1,2,3,4,5)
YC(inactive)=YCntr(1,2,3,4,5)
ZC(inactive)=ZCntr(1,2,3,4,5)
X1(inactive)=X(1)-XC
X2(inactive)=X(2)-XC
X3(inactive)=X(3)-XC
X4(inactive)=X(4)-XC
X5(inactive)=X(5)-XC
Y1(inactive)=Y(1)-YC
Y2(inactive)=Y(2)-YC
Y3(inactive)=Y(3)-YC
Y4(inactive)=Y(4)-YC
Y5(inactive)=Y(5)-YC
Z1(inactive)=Z(1)-ZC
Z2(inactive)=Z(2)-ZC
Z3(inactive)=Z(3)-ZC
Z4(inactive)=Z(4)-ZC
Z5(inactive)=Z(5)-ZC
Rpx(inactive)=0.95106*(X2-X5)+0.58779*(X3-X4)
Rpy(inactive)=0.95106*(Y2-Y5)+0.58779*(Y3-Y4)
Rpz(inactive)=0.95106*(Z2-Z5)+0.58779*(Z3-Z4)
Rppx(inactive)=X1+0.30902*(X2+X5)-0.80902*(X3+X4)
Rppy(inactive)=Y1+0.30902*(Y2+Y5)-0.80902*(Y3+Y4)
Rppz(inactive)=Z1+0.30902*(Z2+Z5)-0.80902*(Z3+Z4)
prx(inactive)=Rpy*Rppz-Rppy*Rpz
pry(inactive)=Rpz*Rppx-Rppz*Rpx
prz(inactive)=Rpx*Rppy-Rppx*Rpy

nx(inactive)=prx/SQRT(prx*prx+pry*pry+prz*prz)
ny(inactive)=pry/SQRT(prx*prx+pry*pry+prz*prz)
nz(inactive)=prz/SQRT(prx*prx+pry*pry+prz*prz)
Zt1(inactive)=X1*nx+Y1*ny+Z1*nz
Zt2(inactive)=X2*nx+Y2*ny+Z2*nz
Zt3(inactive)=X3*nx+Y3*ny+Z3*nz
Zt4(inactive)=X4*nx+Y4*ny+Z4*nz
Zt5(inactive)=X5*nx+Y5*ny+Z5*nz
PUCKB(inactive)=SQRT(2/5)*(Zt1-0.80902*(Zt2+Zt5)+0.30902*(Zt3+Zt4))
PUCKA(inactive)=(-1*SQRT(2/5)*(0.58779*(Zt2-Zt5)+0.95106*(Zt4-Zt3)))
q2=0.52918*SQRT(PUCKA*PUCKA+PUCKB*PUCKB)
PHI2=180+(57.29577951*(PUCKA/(SQRT(PUCKA**2)+0.000001))*ARCCOS(PUCKB/(SQRT(PUCKA*PUCKA+PUCKB*PUCKB))))
PHI2(NSteps=118,StepSize=3.0)

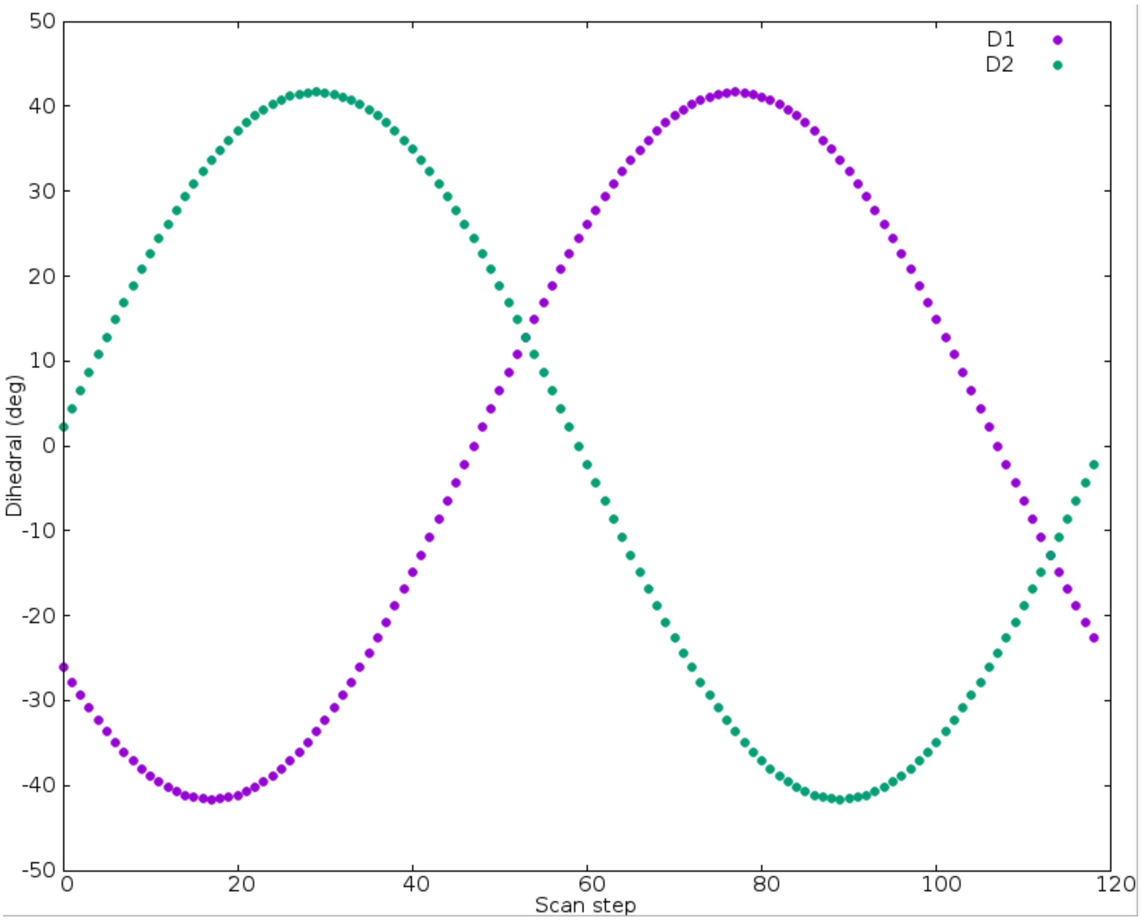

Figure S1. Evolution of the D1 and D2 endocyclic dihedral angles along the scan conformational coordinate of the X=CH2 5-membered ring (C_4H_8X) molecule.

C_4H_8O

```
#p opt=(modredundant,tight) b972/def2svp nosymm maxdisk=8GB geom=GIC scf=xqc

SAM opt

0 1
 C    0.043954     0.215759     0.035008
 C    0.019989    -0.283959     1.475718
 C    1.386687     0.184819     1.994036
 C    2.220584     0.351307     0.702528
 O    1.373816     0.027279    -0.380633
 H   -0.245204     1.285549    -0.013916
 H   -0.613558    -0.342064    -0.645312
 H   -0.823802     0.106708     2.059623
 H   -0.043640    -1.381361     1.483983
 H    1.298600     1.142415     2.525634
 H    1.835770    -0.531779     2.693652
 H    2.593457     1.387475     0.606559
 H    3.092335    -0.317536     0.661499

XC(inactive)=XCntr(1,2,3,4,5)
YC(inactive)=YCntr(1,2,3,4,5)
ZC(inactive)=ZCntr(1,2,3,4,5)
X1(inactive)=X(1)-XC
X2(inactive)=X(2)-XC
X3(inactive)=X(3)-XC
X4(inactive)=X(4)-XC
X5(inactive)=X(5)-XC
Y1(inactive)=Y(1)-YC
Y2(inactive)=Y(2)-YC
Y3(inactive)=Y(3)-YC
Y4(inactive)=Y(4)-YC
Y5(inactive)=Y(5)-YC
Z1(inactive)=Z(1)-ZC
Z2(inactive)=Z(2)-ZC
Z3(inactive)=Z(3)-ZC
Z4(inactive)=Z(4)-ZC
Z5(inactive)=Z(5)-ZC
Rpx(inactive)=0.95106*(X2-X5)+0.58779*(X3-X4)
Rpy(inactive)=0.95106*(Y2-Y5)+0.58779*(Y3-Y4)
Rpz(inactive)=0.95106*(Z2-Z5)+0.58779*(Z3-Z4)
Rppx(inactive)=X1+0.30902*(X2+X5)-0.80902*(X3+X4)
Rppy(inactive)=Y1+0.30902*(Y2+Y5)-0.80902*(Y3+Y4)
Rppz(inactive)=Z1+0.30902*(Z2+Z5)-0.80902*(Z3+Z4)
prx(inactive)=Rpy*Rppz-Rppy*Rpz
pry(inactive)=Rpz*Rppx-Rppz*Rpx
prz(inactive)=Rpx*Rppy-Rppx*Rpy
nx(inactive)=prx/SQRT(prx*prx+pry*pry+prz*prz)
ny(inactive)=pry/SQRT(prx*prx+pry*pry+prz*prz)
nz(inactive)=prz/SQRT(prx*prx+pry*pry+prz*prz)
Zt1(inactive)=X1*nx+Y1*ny+Z1*nz
Zt2(inactive)=X2*nx+Y2*ny+Z2*nz
Zt3(inactive)=X3*nx+Y3*ny+Z3*nz
Zt4(inactive)=X4*nx+Y4*ny+Z4*nz
```

Zt5(inactive)=X5*nx+Y5*ny+Z5*nz
PUCKB(inactive)=SQRT(2/5)*(Zt1-0.80902*(Zt2+Zt5)+0.30902*(Zt3+Zt4))
PUCKA(inactive)=(-1*SQRT(2/5)*(0.58779*(Zt2-Zt5)+0.95106*(Zt4-Zt3)))
q2=0.52918*SQRT(PUCKA*PUCKA+PUCKB*PUCKB)
PHI2=180+(57.29577951*(PUCKA/(SQRT(PUCKA**2)+0.000001))*ARCCOS(PUCKB/(SQRT(PUCKA*PUCKA+PUCKB*PUCKB))))
PHI2(NSteps=118,StepSize=3.0)

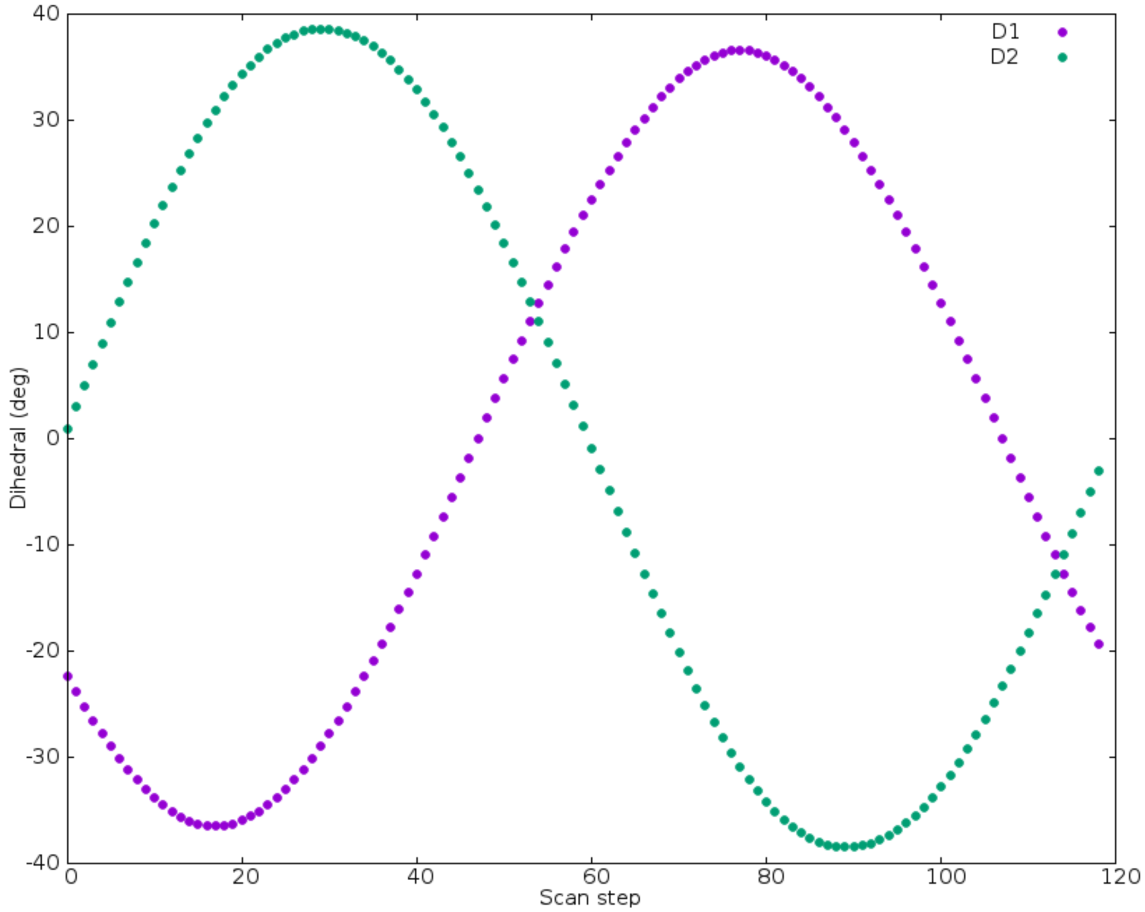

Figure S2. Evolution of the D1 and D2 endocyclic dihedral angles along the scan conformational coordinate of the X=O 5-membered ring (C_4H_8X) molecule.

C_4H_8S

```
#p opt=(modredundant,tight) b972/def2svp nosymm maxdisk=8GB geom=GIC scf=xqc

SAM opt

0 1
 C    0.043954    0.215759    0.035008
 C    0.019989   -0.283959    1.475718
 C    1.386687    0.184819    1.994036
 C    2.220584    0.351307    0.702528
 S    1.373816    0.027279   -0.380633
 H   -0.245204    1.285549   -0.013916
 H   -0.613558   -0.342064   -0.645312
 H   -0.823802    0.106708    2.059623
 H   -0.043640   -1.381361    1.483983
 H    1.298600    1.142415    2.525634
 H    1.835770   -0.531779    2.693652
 H    2.593457    1.387475    0.606559
 H    3.092335   -0.317536    0.661499

XC(inactive)=XCntr(1,2,3,4,5)
YC(inactive)=YCntr(1,2,3,4,5)
ZC(inactive)=ZCntr(1,2,3,4,5)
X1(inactive)=X(1)-XC
X2(inactive)=X(2)-XC
X3(inactive)=X(3)-XC
X4(inactive)=X(4)-XC
X5(inactive)=X(5)-XC
Y1(inactive)=Y(1)-YC
Y2(inactive)=Y(2)-YC
Y3(inactive)=Y(3)-YC
Y4(inactive)=Y(4)-YC
Y5(inactive)=Y(5)-YC
Z1(inactive)=Z(1)-ZC
Z2(inactive)=Z(2)-ZC
Z3(inactive)=Z(3)-ZC
Z4(inactive)=Z(4)-ZC
Z5(inactive)=Z(5)-ZC
Rpx(inactive)=0.95106*(X2-X5)+0.58779*(X3-X4)
Rpy(inactive)=0.95106*(Y2-Y5)+0.58779*(Y3-Y4)
Rpz(inactive)=0.95106*(Z2-Z5)+0.58779*(Z3-Z4)
Rppx(inactive)=X1+0.30902*(X2+X5)-0.80902*(X3+X4)
Rppy(inactive)=Y1+0.30902*(Y2+Y5)-0.80902*(Y3+Y4)
Rppz(inactive)=Z1+0.30902*(Z2+Z5)-0.80902*(Z3+Z4)
prx(inactive)=Rpy*Rppz-Rppy*Rpz
pry(inactive)=Rpz*Rppx-Rppz*Rpx
prz(inactive)=Rpx*Rppy-Rppx*Rpy
nx(inactive)=prx/SQRT(prx*prx+pry*pry+prz*prz)
ny(inactive)=pry/SQRT(prx*prx+pry*pry+prz*prz)
nz(inactive)=prz/SQRT(prx*prx+pry*pry+prz*prz)
Zt1(inactive)=X1*nx+Y1*ny+Z1*nz
Zt2(inactive)=X2*nx+Y2*ny+Z2*nz
Zt3(inactive)=X3*nx+Y3*ny+Z3*nz
Zt4(inactive)=X4*nx+Y4*ny+Z4*nz
```

Zt5(inactive)=X5*nx+Y5*ny+Z5*nz
PUCKB(inactive)=SQRT(2/5)*(Zt1-0.80902*(Zt2+Zt5)+0.30902*(Zt3+Zt4))
PUCKA(inactive)=(-1*SQRT(2/5)*(0.58779*(Zt2-Zt5)+0.95106*(Zt4-Zt3)))
q2=0.52918*SQRT(PUCKA*PUCKA+PUCKB*PUCKB)
PHI2=180+(57.29577951*(PUCKA/(SQRT(PUCKA**2)+0.000001))*ARCCOS(PUCKB/(SQRT(PUCKA*PUCKA+PUCKB*PUCKB))))
PHI2(NSteps=118,StepSize=3.0)

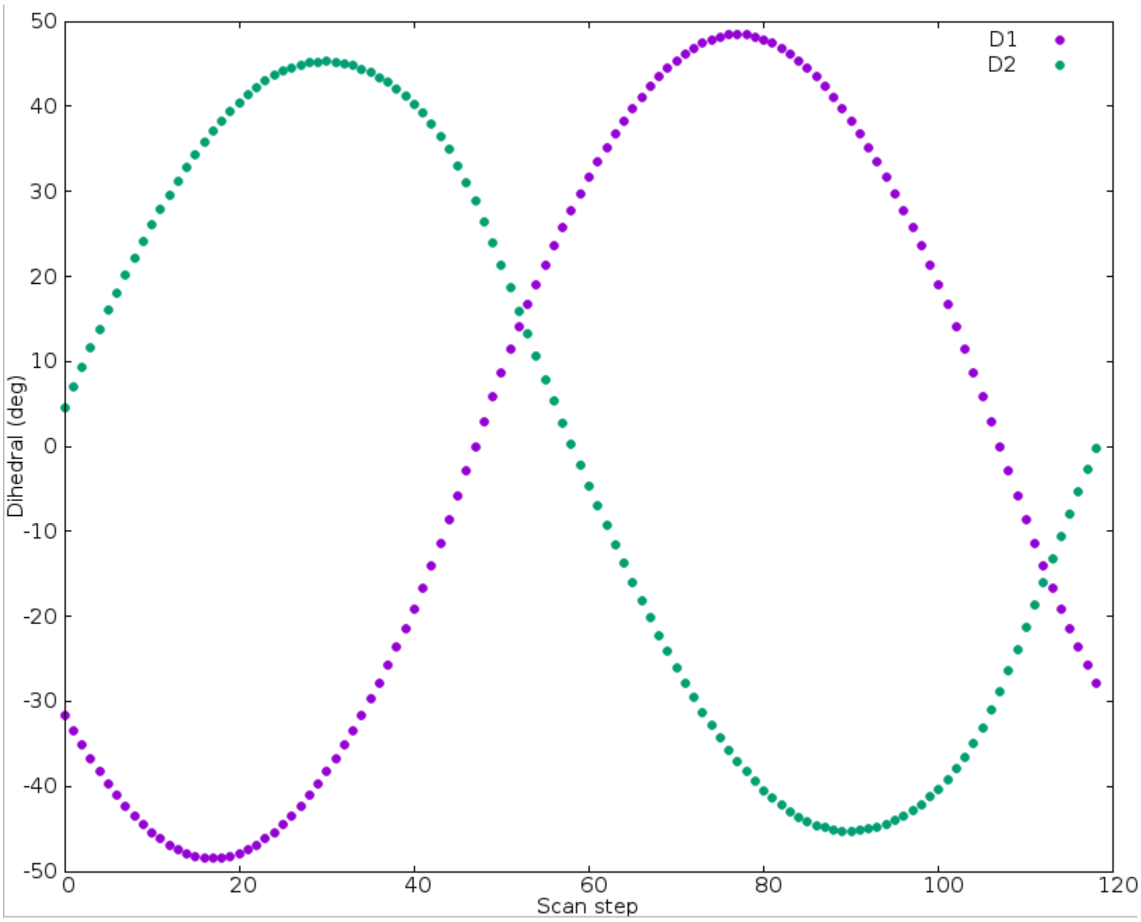

Figure S3. Evolution of the D1 and D2 endocyclic dihedral angles along the scan conformational coordinate of the X=S 5-membered ring (C_4H_8X) molecule.

$C_4H_8SO_2$

#p opt=(modredundant,tight) b972/def2svp nosymm maxdisk=8GB geom=GIC scf=xqc

SAM opt

0 1

C	-0.13728400	0.23611500	0.02763200
C	0.03567700	-0.29829400	1.44289400
C	1.38469800	0.21404200	1.96203500
C	2.37797400	0.34975400	0.78552500
S	1.47871400	-0.03030800	-0.76565600
O	1.82189954	1.04194677	-1.99910377
O	1.68066130	-1.59426248	-1.31535016
H	-0.37463654	1.27923259	0.04929914
H	-0.89987958	-0.31044578	-0.48677960
H	3.18591611	-0.34004702	0.91321249
H	2.75844831	1.34908765	0.74716404
H	1.77539540	-0.47668087	2.67977970
H	1.24847312	1.16914349	2.42477027
H	0.03256361	-1.36822423	1.43107878
H	-0.75526145	0.06155820	2.06725188

```
XC(inactive)=XCntr(1,2,3,4,5)
YC(inactive)=YCntr(1,2,3,4,5)
ZC(inactive)=ZCntr(1,2,3,4,5)
X1(inactive)=X(1)-XC
X2(inactive)=X(2)-XC
X3(inactive)=X(3)-XC
X4(inactive)=X(4)-XC
X5(inactive)=X(5)-XC
Y1(inactive)=Y(1)-YC
Y2(inactive)=Y(2)-YC
Y3(inactive)=Y(3)-YC
Y4(inactive)=Y(4)-YC
Y5(inactive)=Y(5)-YC
Z1(inactive)=Z(1)-ZC
Z2(inactive)=Z(2)-ZC
Z3(inactive)=Z(3)-ZC
Z4(inactive)=Z(4)-ZC
Z5(inactive)=Z(5)-ZC
Rpx(inactive)=0.95106*(X2-X5)+0.58779*(X3-X4)
Rpy(inactive)=0.95106*(Y2-Y5)+0.58779*(Y3-Y4)
Rpz(inactive)=0.95106*(Z2-Z5)+0.58779*(Z3-Z4)
Rppx(inactive)=X1+0.30902*(X2+X5)-0.80902*(X3+X4)
Rppy(inactive)=Y1+0.30902*(Y2+Y5)-0.80902*(Y3+Y4)
Rppz(inactive)=Z1+0.30902*(Z2+Z5)-0.80902*(Z3+Z4)
prx(inactive)=Rpy*Rppz-Rppy*Rpz
pry(inactive)=Rpz*Rppx-Rppz*Rpx
prz(inactive)=Rpx*Rppy-Rppx*Rpy
nx(inactive)=prx/SQRT(prx*prx+pry*pry+prz*prz)
ny(inactive)=pry/SQRT(prx*prx+pry*pry+prz*prz)
nz(inactive)=prz/SQRT(prx*prx+pry*pry+prz*prz)
Zt1(inactive)=X1*nx+Y1*ny+Z1*nz
Zt2(inactive)=X2*nx+Y2*ny+Z2*nz
```

Zt3(inactive)=X3*nx+Y3*ny+Z3*nz
Zt4(inactive)=X4*nx+Y4*ny+Z4*nz
Zt5(inactive)=X5*nx+Y5*ny+Z5*nz
PUCKB(inactive)=SQRT(2/5)*(Zt1-0.80902*(Zt2+Zt5)+0.30902*(Zt3+Zt4))
PUCKA(inactive)=(-1*SQRT(2/5)*(0.58779*(Zt2-Zt5)+0.95106*(Zt4-Zt3)))
q2=0.52918*SQRT(PUCKA*PUCKA+PUCKB*PUCKB)
PHI2=180+(57.29577951*(PUCKA/(SQRT(PUCKA**2)+0.000001))*ARCCOS(PUCKB/(SQRT(PUCKA*PUCKA+PUCKB*PUCKB))))
PHI2(NSteps=118,StepSize=3.0)

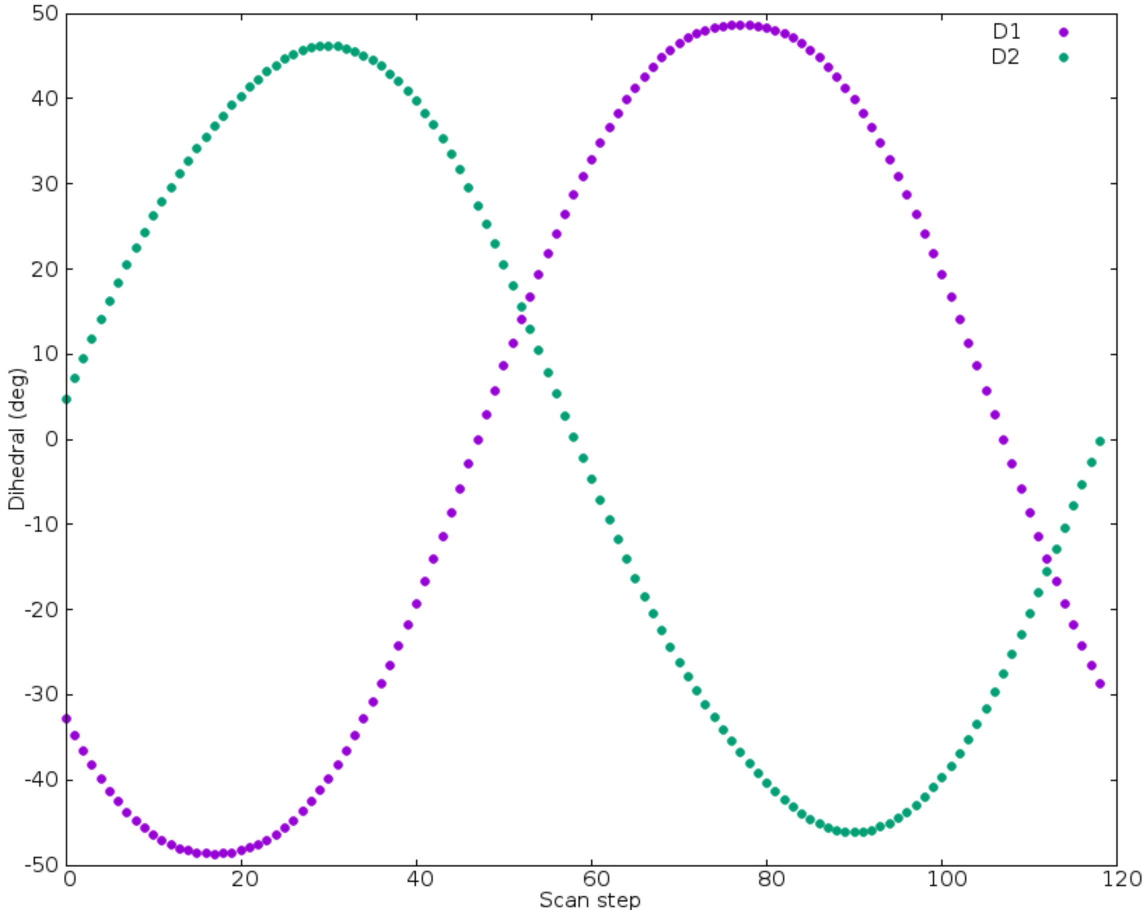

Figure S4. Evolution of the D1 and D2 endocyclic dihedral angles along the scan conformational coordinate of the X=SO_2 5-membered ring (C_4H_8X) molecule.

C_4H_8NH

#p opt=(modredundant,tight) b972/def2svp nosymm maxdisk=8GB geom=GIC scf=xqc

SAM opt

0 1

C	0.04395400	0.21575900	0.03500800
C	0.01998900	-0.28395900	1.47571800
C	1.38668700	0.18481900	1.99403600
C	2.22058400	0.35130700	0.70252800
N	1.37381600	0.02727900	-0.38063300
H	1.66858612	-0.28201330	-1.28476178
H	3.05954622	-0.31253364	0.72144885
H	2.55706406	1.36307161	0.61300957
H	1.82253013	-0.55592893	2.63140031
H	1.29511300	1.11945971	2.50683317
H	-0.05536858	-1.35079583	1.50858776
H	-0.77483766	0.17489339	2.02581350
H	-0.21636687	1.25284064	-0.00492588
H	-0.62225705	-0.36139656	-0.57158437

XC(inactive)=XCntr(1,2,3,4,5)
YC(inactive)=YCntr(1,2,3,4,5)
ZC(inactive)=ZCntr(1,2,3,4,5)
X1(inactive)=X(1)-XC
X2(inactive)=X(2)-XC
X3(inactive)=X(3)-XC
X4(inactive)=X(4)-XC
X5(inactive)=X(5)-XC
Y1(inactive)=Y(1)-YC
Y2(inactive)=Y(2)-YC
Y3(inactive)=Y(3)-YC
Y4(inactive)=Y(4)-YC
Y5(inactive)=Y(5)-YC
Z1(inactive)=Z(1)-ZC
Z2(inactive)=Z(2)-ZC
Z3(inactive)=Z(3)-ZC
Z4(inactive)=Z(4)-ZC
Z5(inactive)=Z(5)-ZC
Rpx(inactive)=0.95106*(X2-X5)+0.58779*(X3-X4)
Rpy(inactive)=0.95106*(Y2-Y5)+0.58779*(Y3-Y4)
Rpz(inactive)=0.95106*(Z2-Z5)+0.58779*(Z3-Z4)
Rppx(inactive)=X1+0.30902*(X2+X5)-0.80902*(X3+X4)
Rppy(inactive)=Y1+0.30902*(Y2+Y5)-0.80902*(Y3+Y4)
Rppz(inactive)=Z1+0.30902*(Z2+Z5)-0.80902*(Z3+Z4)
prx(inactive)=Rpy*Rppz-Rppy*Rpz
pry(inactive)=Rpz*Rppx-Rppz*Rpx
prz(inactive)=Rpx*Rppy-Rppx*Rpy
nx(inactive)=prx/SQRT(prx*prx+pry*pry+prz*prz)
ny(inactive)=pry/SQRT(prx*prx+pry*pry+prz*prz)
nz(inactive)=prz/SQRT(prx*prx+pry*pry+prz*prz)
Zt1(inactive)=X1*nx+Y1*ny+Z1*nz
Zt2(inactive)=X2*nx+Y2*ny+Z2*nz
Zt3(inactive)=X3*nx+Y3*ny+Z3*nz

Zt4(inactive)=X4*nx+Y4*ny+Z4*nz
Zt5(inactive)=X5*nx+Y5*ny+Z5*nz
PUCKB(inactive)=SQRT(2/5)*(Zt1-0.80902*(Zt2+Zt5)+0.30902*(Zt3+Zt4))
PUCKA(inactive)=(-1*SQRT(2/5)*(0.58779*(Zt2-Zt5)+0.95106*(Zt4-Zt3)))
q2=0.52918*SQRT(PUCKA*PUCKA+PUCKB*PUCKB)
PHI2=180+(57.29577951*(PUCKA/(SQRT(PUCKA**2)+0.000001))*ARCCOS(PUCKB/(SQRT(PUCKA*PUCKA+PUCKB*PUCKB))))
PHI2(NSteps=118,StepSize=3.0)

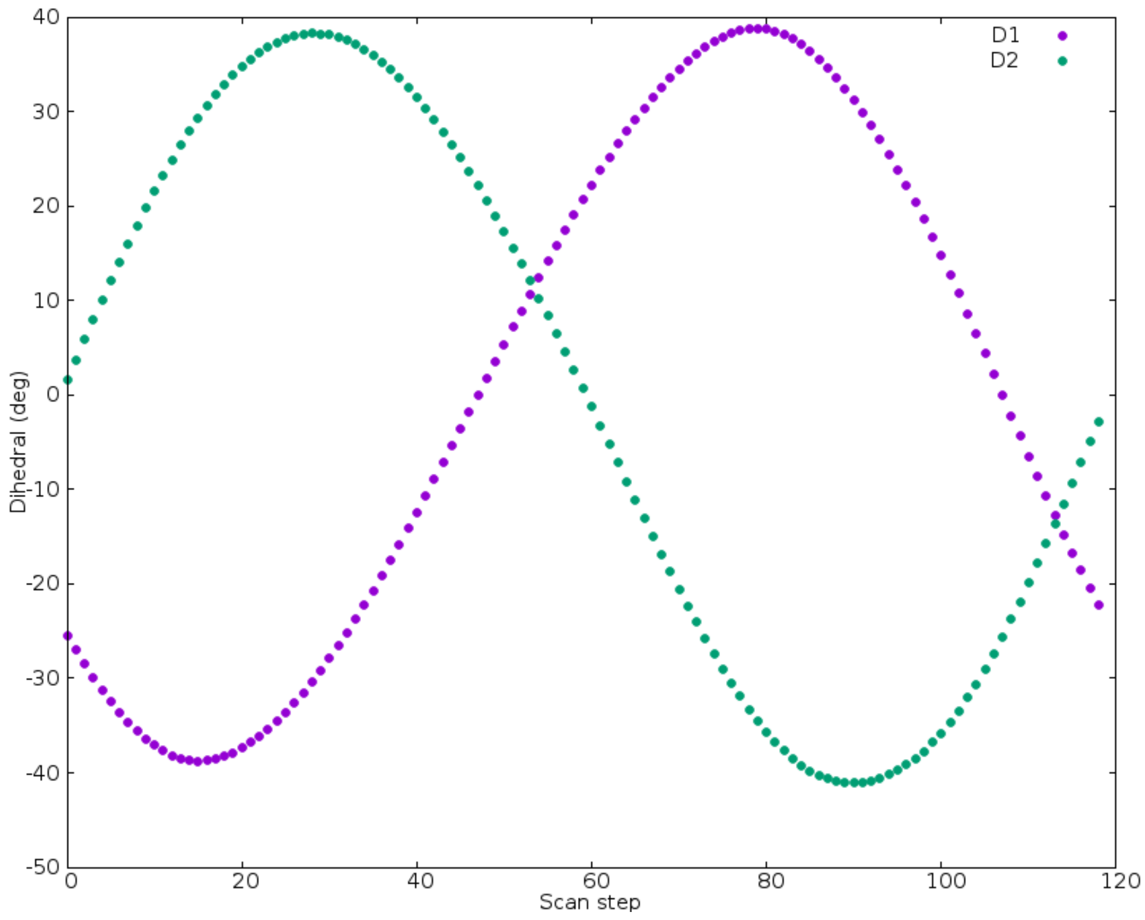

Figure S5. Evolution of the D1 and D2 endocyclic dihedral angles along the scan conformational coordinate of the X=NH 5-membered ring (C_4H_8X) molecule.

C_4H_8BH

#p opt=(modredundant,tight) b972/def2svp nosymm maxdisk=8GB geom=GIC scf=xqc

SAM opt

0 1

C	0.04395400	0.21575900	0.03500800
C	0.01998900	-0.28395900	1.47571800
C	1.38668700	0.18481900	1.99403600
C	2.22058400	0.35130700	0.70252800
B	1.37381600	0.02727900	-0.38063300
H	1.66858612	-0.28201330	-1.28476178
H	3.05954622	-0.31253364	0.72144885
H	2.55706406	1.36307161	0.61300957
H	1.82253013	-0.55592893	2.63140031
H	1.29511300	1.11945971	2.50683317
H	-0.05536858	-1.35079583	1.50858776
H	-0.77483766	0.17489339	2.02581350
H	-0.21636687	1.25284064	-0.00492588
H	-0.62225705	-0.36139656	-0.57158437

```
XC(inactive)=XCntr(1,2,3,4,5)
YC(inactive)=YCntr(1,2,3,4,5)
ZC(inactive)=ZCntr(1,2,3,4,5)
X1(inactive)=X(1)-XC
X2(inactive)=X(2)-XC
X3(inactive)=X(3)-XC
X4(inactive)=X(4)-XC
X5(inactive)=X(5)-XC
Y1(inactive)=Y(1)-YC
Y2(inactive)=Y(2)-YC
Y3(inactive)=Y(3)-YC
Y4(inactive)=Y(4)-YC
Y5(inactive)=Y(5)-YC
Z1(inactive)=Z(1)-ZC
Z2(inactive)=Z(2)-ZC
Z3(inactive)=Z(3)-ZC
Z4(inactive)=Z(4)-ZC
Z5(inactive)=Z(5)-ZC
Rpx(inactive)=0.95106*(X2-X5)+0.58779*(X3-X4)
Rpy(inactive)=0.95106*(Y2-Y5)+0.58779*(Y3-Y4)
Rpz(inactive)=0.95106*(Z2-Z5)+0.58779*(Z3-Z4)
Rppx(inactive)=X1+0.30902*(X2+X5)-0.80902*(X3+X4)
Rppy(inactive)=Y1+0.30902*(Y2+Y5)-0.80902*(Y3+Y4)
Rppz(inactive)=Z1+0.30902*(Z2+Z5)-0.80902*(Z3+Z4)
prx(inactive)=Rpy*Rppz-Rppy*Rpz
pry(inactive)=Rpz*Rppx-Rppz*Rpx
prz(inactive)=Rpx*Rppy-Rppx*Rpy
nx(inactive)=prx/SQRT(prx*prx+pry*pry+prz*prz)
ny(inactive)=pry/SQRT(prx*prx+pry*pry+prz*prz)
nz(inactive)=prz/SQRT(prx*prx+pry*pry+prz*prz)
Zt1(inactive)=X1*nx+Y1*ny+Z1*nz
Zt2(inactive)=X2*nx+Y2*ny+Z2*nz
Zt3(inactive)=X3*nx+Y3*ny+Z3*nz
```

Zt4(inactive)=X4*nx+Y4*ny+Z4*nz
Zt5(inactive)=X5*nx+Y5*ny+Z5*nz
PUCKB(inactive)=SQRT(2/5)*(Zt1-0.80902*(Zt2+Zt5)+0.30902*(Zt3+Zt4))
PUCKA(inactive)=(-1*SQRT(2/5)*(0.58779*(Zt2-Zt5)+0.95106*(Zt4-Zt3)))
q2=0.52918*SQRT(PUCKA*PUCKA+PUCKB*PUCKB)
PHI2=180+(57.29577951*(PUCKA/(SQRT(PUCKA**2)+0.000001))*ARCCOS(PUCKB/(SQRT(PUCKA*PUCKA+PUCKB*PUCKB))))
PHI2(NSteps=118,StepSize=3.0)

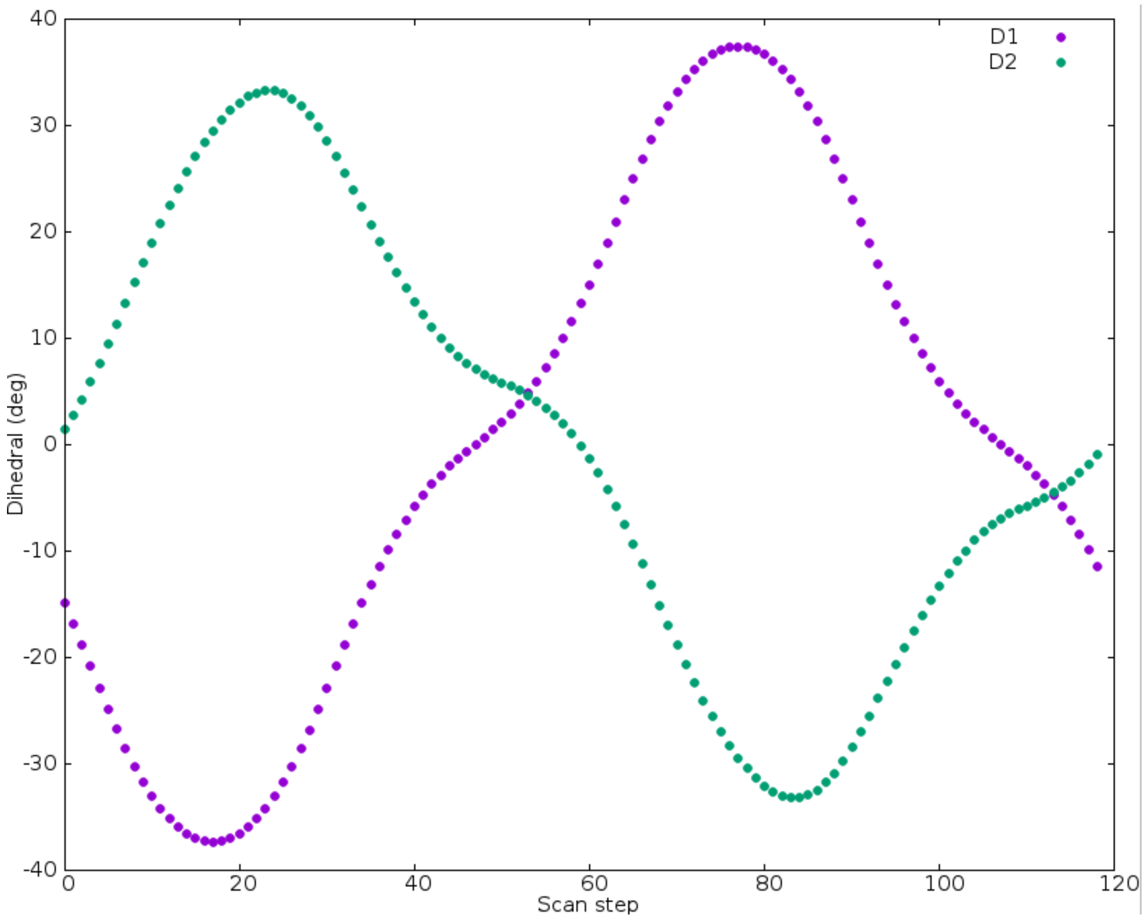

Figure S6. Evolution of the D1 and D2 endocyclic dihedral angles along the scan conformational coordinate of the X=BH 5-membered ring (C_4H_8X) molecule.

C_4H_8PH

#p opt=(modredundant,tight) b972/def2svp nosymm maxdisk=8GB geom=GIC scf=xqc

SAM opt

0 1

C	0.04395400	0.21575900	0.03500800
C	0.01998900	-0.28395900	1.47571800
C	1.38668700	0.18481900	1.99403600
C	2.22058400	0.35130700	0.70252800
P	1.37381600	0.02727900	-0.38063300
H	1.66858612	-0.28201330	-1.28476178
H	3.05954622	-0.31253364	0.72144885
H	2.55706406	1.36307161	0.61300957
H	1.82253013	-0.55592893	2.63140031
H	1.29511300	1.11945971	2.50683317
H	-0.05536858	-1.35079583	1.50858776
H	-0.77483766	0.17489339	2.02581350
H	-0.21636687	1.25284064	-0.00492588
H	-0.62225705	-0.36139656	-0.57158437

```
XC(inactive)=XCntr(1,2,3,4,5)
YC(inactive)=YCntr(1,2,3,4,5)
ZC(inactive)=ZCntr(1,2,3,4,5)
X1(inactive)=X(1)-XC
X2(inactive)=X(2)-XC
X3(inactive)=X(3)-XC
X4(inactive)=X(4)-XC
X5(inactive)=X(5)-XC
Y1(inactive)=Y(1)-YC
Y2(inactive)=Y(2)-YC
Y3(inactive)=Y(3)-YC
Y4(inactive)=Y(4)-YC
Y5(inactive)=Y(5)-YC
Z1(inactive)=Z(1)-ZC
Z2(inactive)=Z(2)-ZC
Z3(inactive)=Z(3)-ZC
Z4(inactive)=Z(4)-ZC
Z5(inactive)=Z(5)-ZC
Rpx(inactive)=0.95106*(X2-X5)+0.58779*(X3-X4)
Rpy(inactive)=0.95106*(Y2-Y5)+0.58779*(Y3-Y4)
Rpz(inactive)=0.95106*(Z2-Z5)+0.58779*(Z3-Z4)
Rppx(inactive)=X1+0.30902*(X2+X5)-0.80902*(X3+X4)
Rppy(inactive)=Y1+0.30902*(Y2+Y5)-0.80902*(Y3+Y4)
Rppz(inactive)=Z1+0.30902*(Z2+Z5)-0.80902*(Z3+Z4)
prx(inactive)=Rpy*Rppz-Rppy*Rpz
pry(inactive)=Rpz*Rppx-Rppz*Rpx
prz(inactive)=Rpx*Rppy-Rppx*Rpy
nx(inactive)=prx/SQRT(prx*prx+pry*pry+prz*prz)
ny(inactive)=pry/SQRT(prx*prx+pry*pry+prz*prz)
nz(inactive)=prz/SQRT(prx*prx+pry*pry+prz*prz)
Zt1(inactive)=X1*nx+Y1*ny+Z1*nz
Zt2(inactive)=X2*nx+Y2*ny+Z2*nz
Zt3(inactive)=X3*nx+Y3*ny+Z3*nz
```

Zt4(inactive)=X4*nx+Y4*ny+Z4*nz
Zt5(inactive)=X5*nx+Y5*ny+Z5*nz
PUCKB(inactive)=SQRT(2/5)*(Zt1-0.80902*(Zt2+Zt5)+0.30902*(Zt3+Zt4))
PUCKA(inactive)=(-1*SQRT(2/5)*(0.58779*(Zt2-Zt5)+0.95106*(Zt4-Zt3)))
q2=0.52918*SQRT(PUCKA*PUCKA+PUCKB*PUCKB)
PHI2=180+(57.29577951*(PUCKA/(SQRT(PUCKA**2)+0.000001))*ARCCOS(PUCKB/(SQRT(PUCKA*PUCKA+PUCKB*PUCKB))))
PHI2(NSteps=118,StepSize=3.0)

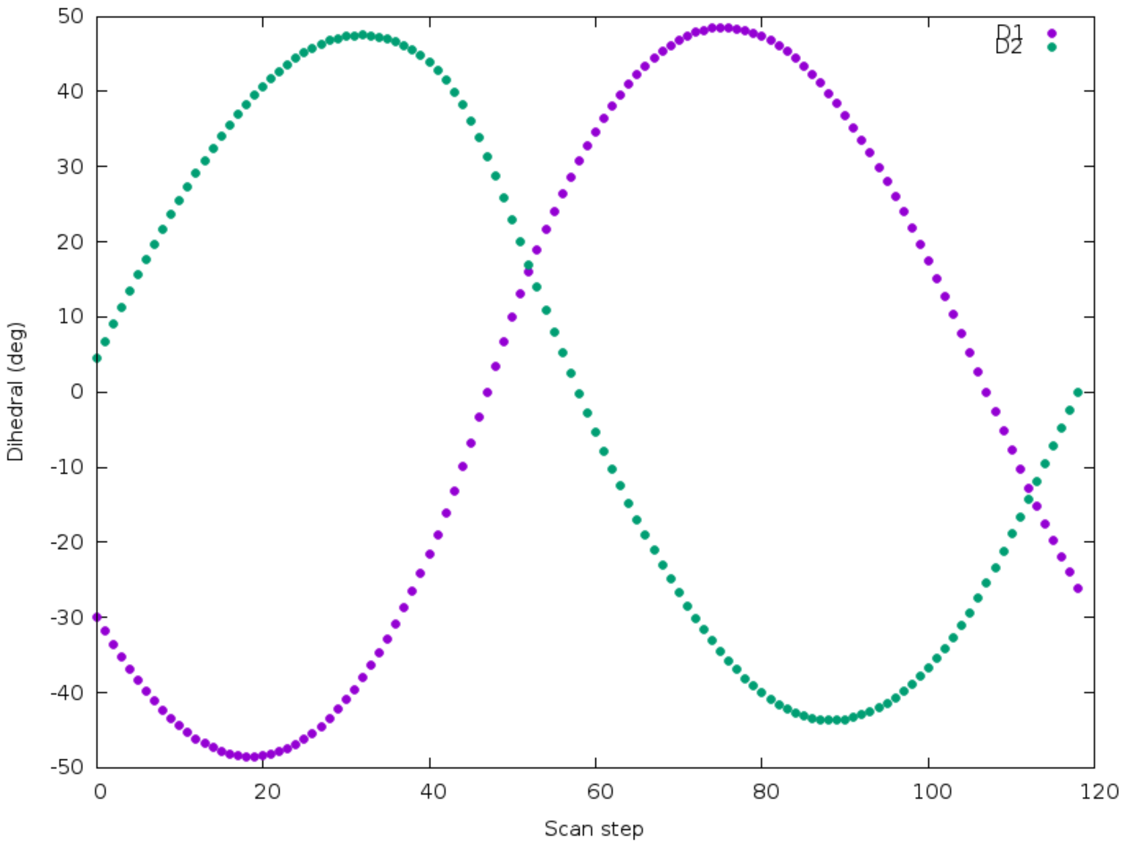

Figure S7. Evolution of the D1 and D2 endocyclic dihedral angles along the scan conformational coordinate of the X=PH 5-membered ring (C_4H_8X) molecule.

6-membered rings

C_6H_{12} - Optimization $^4C_1$, optimization $^1C_4$, scan at θ=90º (B), scan at θ=45º and θ=135º (E/H)

#p opt=(modredundant,tight) b972/def2svp nosymm maxdisk=8GB geom=GIC scf=xqc

SAM opt

0 1

H	-2.18647900	1.07639900	-1.74757400
C	-4.82561000	-1.12818200	-1.29147600
C	-2.74414900	0.26887500	-1.25065000
C	-1.96252600	-1.04394900	-1.30013900
C	-2.80527500	-2.18522800	-0.74191700
H	-5.77027900	-1.33607900	-1.81248900
H	-2.29936300	-3.15229300	-0.86812200
H	-1.01945900	-0.96655900	-0.73798200
H	-5.07199000	-0.93157100	-0.22739800
H	-2.86783200	0.57898400	-0.19879000
H	-1.69701200	-1.27937100	-2.34307200
H	-2.96291200	-2.03479300	0.34625600
C	-4.12185100	0.08598400	-1.88726800
H	-4.74493400	0.98294500	-1.75078400
H	-4.01234300	-0.06754000	-2.97264900
C	-4.04489000	-2.29349300	-1.39795600
H	-4.58841425	-3.10872902	-0.96798900
H	-3.86628503	-2.49102016	-2.43428765

#p opt=(modredundant,tight) b972/def2svp nosymm maxdisk=8GB geom=GIC scf=xqc

SAM opt

0 1

H	-2.58863500	-0.26926200	-2.83789100
C	-4.82203000	-1.13376400	-1.35210800
C	-2.67880800	0.08853500	-1.79782600
C	-2.00885000	-0.91554700	-0.85980900
C	-2.81115600	-2.21090300	-0.80732700
H	-4.85767000	-1.59800900	-2.35948200
H	-2.75875500	-2.72228000	-1.79087400
H	-1.95271900	-0.49442100	0.15661600
H	-5.85727900	-1.05144600	-0.99354300
H	-2.16762500	1.06183200	-1.76031000
H	-0.97661800	-1.12828100	-1.17703200
H	-2.40235500	-2.90215300	-0.05756600
C	-4.15825100	0.23576300	-1.44215600
H	-4.25787300	0.74038000	-0.46796200
H	-4.68479100	0.85799100	-2.18173800
C	-4.15348700	-1.98430200	-0.45299800
H	-4.66355803	-2.92460642	-0.42943648
H	-4.17917786	-1.54012095	0.52011205

```
#p opt=(modredundant,tight) b972/def2svp nosymm maxdisk=8GB geom=GIC scf=xqc

SAM opt

0 1
 H    -2.20132200   1.09061200  -1.76313500
 C    -4.82621600  -1.12969500  -1.22101600
 C    -2.74543700   0.29108000  -1.24050800
 C    -1.96562300  -1.02053400  -1.30850500
 C    -2.79538300  -2.16265600  -0.67465300
 H    -5.05931200  -1.88035700  -2.00164100
 H    -2.93392800  -2.99011700  -1.39793700
 H    -0.99661700  -0.91793100  -0.79971200
 H    -5.77665300  -0.85637700  -0.74421300
 H    -2.84056200   0.60713300  -0.19123500
 H    -1.73890300  -1.25311300  -2.36064900
 H    -2.28603800  -2.58322700   0.20242500
 C    -4.13642700   0.10669600  -1.84584300
 H    -4.74257100   1.01021500  -1.68874500
 H    -4.04613600  -0.01985800  -2.93580300
 C    -4.04577500  -1.71773900  -0.21083900
 H    -4.57438502  -2.55712312   0.19029160
 H    -3.89087756  -0.99217201   0.56017288

XC(inactive)=XCntr(16,5,4,3,13,2)
YC(inactive)=YCntr(16,5,4,3,13,2)
ZC(inactive)=ZCntr(16,5,4,3,13,2)
X1(inactive)=X(16)-XC
X2(inactive)=X(5)-XC
X3(inactive)=X(4)-XC
X4(inactive)=X(3)-XC
X5(inactive)=X(13)-XC
X6(inactive)=X(2)-XC
Y1(inactive)=Y(16)-YC
Y2(inactive)=Y(5)-YC
Y3(inactive)=Y(4)-YC
Y4(inactive)=Y(3)-YC
Y5(inactive)=Y(13)-YC
Y6(inactive)=Y(2)-YC
Z1(inactive)=Z(16)-ZC
Z2(inactive)=Z(5)-ZC
Z3(inactive)=Z(4)-ZC
Z4(inactive)=Z(3)-ZC
Z5(inactive)=Z(13)-ZC
Z6(inactive)=Z(2)-ZC
Rpx(inactive)=0.866025403*(X2+X3-X5-X6)
Rpy(inactive)=0.866025403*(Y2+Y3-Y5-Y6)
Rpz(inactive)=0.866025403*(Z2+Z3-Z5-Z6)
Rppx(inactive)=X1+0.5*(X2+X6-X3-X5)
Rppy(inactive)=Y1+0.5*(Y2+Y6-Y3-Y5)
Rppz(inactive)=Z1+0.5*(Z2+Z6-Z3-Z5)
prx(inactive)=Rpy*Rppz-Rppy*Rpz
pry(inactive)=Rpz*Rppx-Rppz*Rpx
prz(inactive)=Rpx*Rppy-Rppx*Rpy
nx(inactive)=prx/SQRT(prx*prx+pry*pry+prz*prz)
```

ny(inactive)=pry/SQRT(prx*prx+pry*pry+prz*prz)
nz(inactive)=prz/SQRT(prx*prx+pry*pry+prz*prz)
Zt1(inactive)=X1*nx+Y1*ny+Z1*nz
Zt2(inactive)=X2*nx+Y2*ny+Z2*nz
Zt3(inactive)=X3*nx+Y3*ny+Z3*nz
Zt4(inactive)=X4*nx+Y4*ny+Z4*nz
Zt5(inactive)=X5*nx+Y5*ny+Z5*nz
Zt6(inactive)=X6*nx+Y6*ny+Z6*nz
PUCKB(inactive)=SQRT(1/3)*(Zt1+Zt4-0.5*(Zt2+Zt3+Zt5+Zt6))
PUCKA(inactive)=-1*SQRT(1/3)*0.866025403*(Zt2+Zt5-Zt3-Zt6)
q2(inactive)=SQRT(PUCKA*PUCKA+PUCKB*PUCKB)
q3(inactive)=SQRT(1/6)*(Zt1-Zt2+Zt3-Zt4+Zt5-Zt6)
PHI=180+(57.29577951*(PUCKA/(SQRT(PUCKA**2)+0.0001))*ARCCOS(PUCKB/q2))
THETA=57.29577951*ARCCOS(q3/SQRT(q2*q2+q3*q3))
Qrad=SQRT(q2*q2+q3*q3)
PHI(NSteps=118,StepSize=-3.0)

#p opt=(modredundant,tight) b972/def2svp nosymm maxdisk=8GB geom=GIC scf=xqc

SAM opt

0 1

H	-2.21835700	1.11493700	-1.61016300
C	-4.87832600	-1.10544800	-1.29004100
C	-2.76373600	0.26246900	-1.18063300
C	-1.98807700	-1.03751200	-1.36406000
C	-2.72793100	-2.18397000	-0.68822300
H	-5.62445900	-1.47210700	-2.01218300
H	-2.33578200	-3.15506200	-1.02869500
H	-0.97474900	-0.98022100	-0.94009500
H	-5.44793400	-0.73630500	-0.41426400
H	-2.90312700	0.47894000	-0.10796800
H	-1.87053000	-1.23231800	-2.44238700
H	-2.51907200	-2.12515700	0.39918700
C	-4.10709500	0.07617200	-1.87602500
H	-4.73947200	0.97304800	-1.80378200
H	-3.91666500	-0.08564600	-2.94944500
C	-4.12159000	-2.21768700	-0.87323300
H	-4.53304892	-2.51628682	0.06827627
H	-4.30119296	-2.98252881	-1.59963494

XC(inactive)=XCntr(16,5,4,3,13,2)
YC(inactive)=YCntr(16,5,4,3,13,2)
ZC(inactive)=ZCntr(16,5,4,3,13,2)
X1(inactive)=X(16)-XC
X2(inactive)=X(5)-XC
X3(inactive)=X(4)-XC
X4(inactive)=X(3)-XC
X5(inactive)=X(13)-XC
X6(inactive)=X(2)-XC
Y1(inactive)=Y(16)-YC
Y2(inactive)=Y(5)-YC
Y3(inactive)=Y(4)-YC
Y4(inactive)=Y(3)-YC
Y5(inactive)=Y(13)-YC

Y6(inactive)=Y(2)-YC
Z1(inactive)=Z(16)-ZC
Z2(inactive)=Z(5)-ZC
Z3(inactive)=Z(4)-ZC
Z4(inactive)=Z(3)-ZC
Z5(inactive)=Z(13)-ZC
Z6(inactive)=Z(2)-ZC
Rpx(inactive)=0.866025403*(X2+X3-X5-X6)
Rpy(inactive)=0.866025403*(Y2+Y3-Y5-Y6)
Rpz(inactive)=0.866025403*(Z2+Z3-Z5-Z6)
Rppx(inactive)=X1+0.5*(X2+X6-X3-X5)
Rppy(inactive)=Y1+0.5*(Y2+Y6-Y3-Y5)
Rppz(inactive)=Z1+0.5*(Z2+Z6-Z3-Z5)
prx(inactive)=Rpy*Rppz-Rppy*Rpz
pry(inactive)=Rpz*Rppx-Rppz*Rpx
prz(inactive)=Rpx*Rppy-Rppx*Rpy
nx(inactive)=prx/SQRT(prx*prx+pry*pry+prz*prz)
ny(inactive)=pry/SQRT(prx*prx+pry*pry+prz*prz)
nz(inactive)=prz/SQRT(prx*prx+pry*pry+prz*prz)
Zt1(inactive)=X1*nx+Y1*ny+Z1*nz
Zt2(inactive)=X2*nx+Y2*ny+Z2*nz
Zt3(inactive)=X3*nx+Y3*ny+Z3*nz
Zt4(inactive)=X4*nx+Y4*ny+Z4*nz
Zt5(inactive)=X5*nx+Y5*ny+Z5*nz
Zt6(inactive)=X6*nx+Y6*ny+Z6*nz
PUCKB(inactive)=SQRT(1/3)*(Zt1+Zt4-0.5*(Zt2+Zt3+Zt5+Zt6))
PUCKA(inactive)=-1*SQRT(1/3)*0.866025403*(Zt2+Zt5-Zt3-Zt6)
q2(inactive)=SQRT(PUCKA*PUCKA+PUCKB*PUCKB)
q3(inactive)=SQRT(1/6)*(Zt1-Zt2+Zt3-Zt4+Zt5-Zt6)
PHI=180+(57.29577951*(PUCKA/(SQRT(PUCKA**2)+0.0001))*ARCCOS(PUCKB/q2))
THETA(frozen)=57.29577951*ARCCOS(q3/SQRT(q2*q2+q3*q3))
Qrad=SQRT(q2*q2+q3*q3)
PHI(NSteps=30,StepSize=-11.4453)

#p opt=(modredundant,tight) b972/def2svp nosymm maxdisk=8GB geom=GIC scf=xqc

SAM opt

0 1

H	-2.40404200	0.26258700	-2.70919500
C	-4.81240300	-1.18076200	-1.30877100
C	-2.68313000	0.21894700	-1.64447700
C	-1.95311400	-0.98878000	-1.01287600
C	-2.83623300	-2.20499900	-0.73924400
H	-4.79367800	-1.79966400	-2.22864300
H	-2.93962700	-2.82692800	-1.65174100
H	-1.53300500	-0.67312700	-0.04539200
H	-5.86138900	-1.09949600	-0.99243000
H	-2.30017300	1.15108200	-1.20971200
H	-1.09346200	-1.28502900	-1.63040600
H	-2.38150700	-2.84007900	0.03305900
C	-4.22357500	0.19980800	-1.56343300
H	-4.54585900	0.84049600	-0.72789500
H	-4.66241800	0.63945000	-2.47073400

C	-4.11328700	-1.83937400	-0.28012000
H	-4.64591548	-2.71973896	0.01341778
H	-4.02068780	-1.18208320	0.55910237

```
XC(inactive)=XCntr(16,5,4,3,13,2)
YC(inactive)=YCntr(16,5,4,3,13,2)
ZC(inactive)=ZCntr(16,5,4,3,13,2)
X1(inactive)=X(16)-XC
X2(inactive)=X(5)-XC
X3(inactive)=X(4)-XC
X4(inactive)=X(3)-XC
X5(inactive)=X(13)-XC
X6(inactive)=X(2)-XC
Y1(inactive)=Y(16)-YC
Y2(inactive)=Y(5)-YC
Y3(inactive)=Y(4)-YC
Y4(inactive)=Y(3)-YC
Y5(inactive)=Y(13)-YC
Y6(inactive)=Y(2)-YC
Z1(inactive)=Z(16)-ZC
Z2(inactive)=Z(5)-ZC
Z3(inactive)=Z(4)-ZC
Z4(inactive)=Z(3)-ZC
Z5(inactive)=Z(13)-ZC
Z6(inactive)=Z(2)-ZC
Rpx(inactive)=0.866025403*(X2+X3-X5-X6)
Rpy(inactive)=0.866025403*(Y2+Y3-Y5-Y6)
Rpz(inactive)=0.866025403*(Z2+Z3-Z5-Z6)
Rppx(inactive)=X1+0.5*(X2+X6-X3-X5)
Rppy(inactive)=Y1+0.5*(Y2+Y6-Y3-Y5)
Rppz(inactive)=Z1+0.5*(Z2+Z6-Z3-Z5)
prx(inactive)=Rpy*Rppz-Rppy*Rpz
pry(inactive)=Rpz*Rppx-Rppz*Rpx
prz(inactive)=Rpx*Rppy-Rppx*Rpy
nx(inactive)=prx/SQRT(prx*prx+pry*pry+prz*prz)
ny(inactive)=pry/SQRT(prx*prx+pry*pry+prz*prz)
nz(inactive)=prz/SQRT(prx*prx+pry*pry+prz*prz)
Zt1(inactive)=X1*nx+Y1*ny+Z1*nz
Zt2(inactive)=X2*nx+Y2*ny+Z2*nz
Zt3(inactive)=X3*nx+Y3*ny+Z3*nz
Zt4(inactive)=X4*nx+Y4*ny+Z4*nz
Zt5(inactive)=X5*nx+Y5*ny+Z5*nz
Zt6(inactive)=X6*nx+Y6*ny+Z6*nz
PUCKB(inactive)=SQRT(1/3)*(Zt1+Zt4-0.5*(Zt2+Zt3+Zt5+Zt6))
PUCKA(inactive)=-1*SQRT(1/3)*0.866025403*(Zt2+Zt5-Zt3-Zt6)
q2(inactive)=SQRT(PUCKA*PUCKA+PUCKB*PUCKB)
q3(inactive)=SQRT(1/6)*(Zt1-Zt2+Zt3-Zt4+Zt5-Zt6)
PHI=180+(57.29577951*(PUCKA/(SQRT(PUCKA**2)+0.0001))*ARCCOS(PUCKB/q2))
THETA(frozen)=57.29577951*ARCCOS(q3/SQRT(q2*q2+q3*q3))
Qrad=SQRT(q2*q2+q3*q3)
PHI(NSteps=30,StepSize=-11.4453)
```

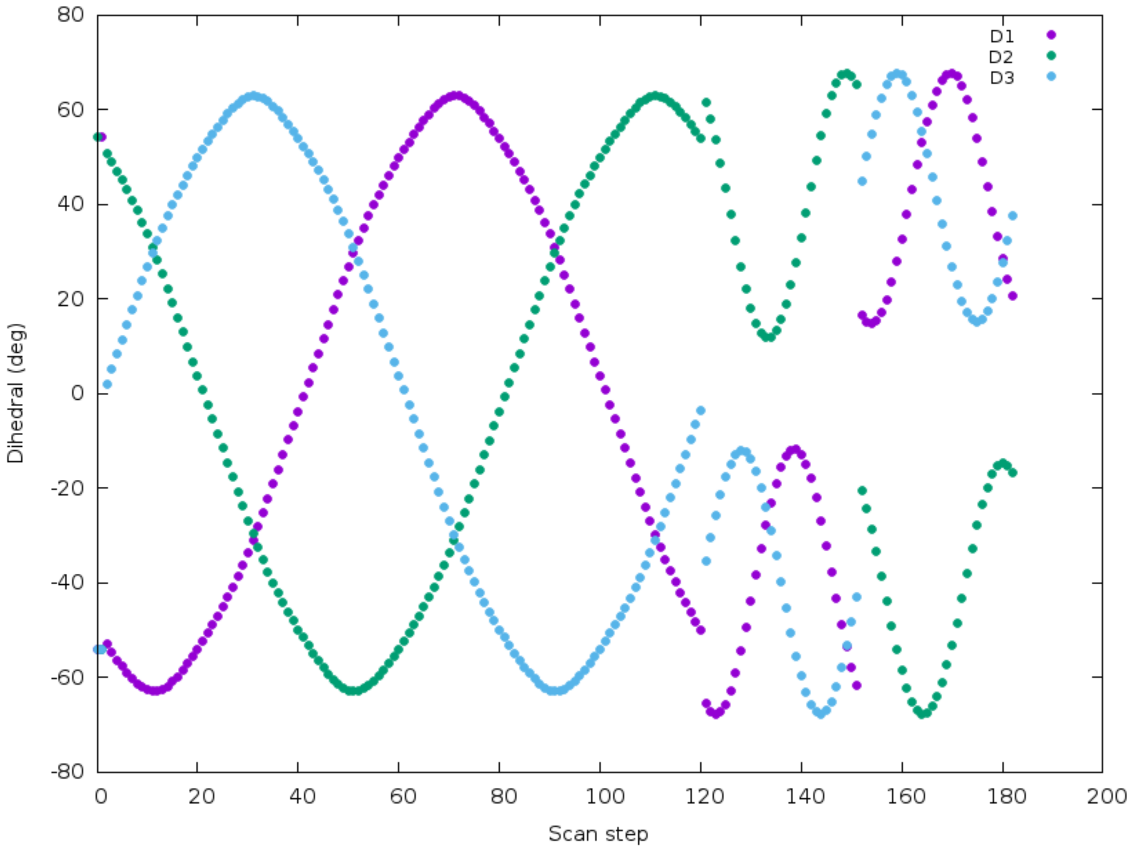

Figure S8. Evolution of the D1, D2 and D3 endocyclic dihedral angles along the scan conformational coordinate of the X=CH_2 6-membered ring ($C_5H_{10}X$) molecule.

$C_5H_{10}O$ - Optimization $^4C_1$, optimization $^1C_4$, Scan at θ=90º (B), Scan at θ=45º and θ=135º (E)

#p opt=(modredundant,tight) b972/def2svp nosymm maxdisk=8GB geom=GIC scf=xqc

SAM opt

0 1

H	-2.188171	1.081748	-1.730567
C	-4.827145	-1.126549	-1.292515
C	-2.745652	0.268165	-1.243384
C	-1.963903	-1.044173	-1.306489
C	-2.802507	-2.183911	-0.738883
H	-5.767353	-1.338192	-1.820045
H	-2.299108	-3.151937	-0.867796
H	-1.014726	-0.969840	-0.754263
H	-5.083547	-0.919865	-0.232561
H	-2.874305	0.566557	-0.188846
H	-1.710727	-1.276592	-2.353088
H	-2.947620	-2.030321	0.350701
C	-4.118837	0.084401	-1.889708
H	-4.743647	0.981238	-1.760465
H	-4.000688	-0.072185	-2.973728

```
 O    -4.048967    -2.294413    -1.380372

#p opt=(modredundant,tight) b972/def2svp nosymm maxdisk=8GB geom=GIC scf=xqc

SAM opt

0 1
 H    -2.586808    -0.236453    -2.836242
 C    -4.818426    -1.136893    -1.358206
 C    -2.680830     0.098160    -1.788660
 C    -2.004439    -0.922800    -0.873968
 C    -2.818290    -2.209782    -0.796141
 H    -4.827929    -1.614293    -2.359628
 H    -2.781963    -2.736729    -1.772078
 H    -1.915864    -0.505454     0.141921
 H    -5.861314    -1.060887    -1.020946
 H    -2.171174     1.071190    -1.730386
 H    -0.982936    -1.146279    -1.217250
 H    -2.406738    -2.893125    -0.040695
 C    -4.163368     0.236807    -1.443080
 H    -4.275783     0.736980    -0.468052
 H    -4.685484     0.858925    -2.185887
 O    -4.155556    -1.965236    -0.432710

#p opt=(modredundant,tight) b972/def2svp nosymm maxdisk=8GB geom=GIC scf=xqc

SAM opt

0 1
 H    -2.201322     1.090612    -1.763135
 C    -4.826216    -1.129695    -1.221016
 C    -2.745437     0.291080    -1.240508
 C    -1.965623    -1.020534    -1.308505
 C    -2.795383    -2.162656    -0.674653
 H    -5.059312    -1.880357    -2.001641
 H    -2.933928    -2.990117    -1.397937
 H    -0.996617    -0.917931    -0.799712
 H    -5.776653    -0.856377    -0.744213
 H    -2.840562     0.607133    -0.191235
 H    -1.738903    -1.253113    -2.360649
 H    -2.286038    -2.583227     0.202425
 C    -4.136427     0.106696    -1.845843
 H    -4.742571     1.010215    -1.688745
 H    -4.046136    -0.019858    -2.935803
 O    -4.045775    -1.717739    -0.210839

XC(inactive)=XCntr(16,5,4,3,13,2)
YC(inactive)=YCntr(16,5,4,3,13,2)
ZC(inactive)=ZCntr(16,5,4,3,13,2)
X1(inactive)=X(16)-XC
X2(inactive)=X(5)-XC
X3(inactive)=X(4)-XC
X4(inactive)=X(3)-XC
X5(inactive)=X(13)-XC
X6(inactive)=X(2)-XC
```

```
Y1(inactive)=Y(16)-YC
Y2(inactive)=Y(5)-YC
Y3(inactive)=Y(4)-YC
Y4(inactive)=Y(3)-YC
Y5(inactive)=Y(13)-YC
Y6(inactive)=Y(2)-YC
Z1(inactive)=Z(16)-ZC
Z2(inactive)=Z(5)-ZC
Z3(inactive)=Z(4)-ZC
Z4(inactive)=Z(3)-ZC
Z5(inactive)=Z(13)-ZC
Z6(inactive)=Z(2)-ZC
Rpx(inactive)=0.866025403*(X2+X3-X5-X6)
Rpy(inactive)=0.866025403*(Y2+Y3-Y5-Y6)
Rpz(inactive)=0.866025403*(Z2+Z3-Z5-Z6)
Rppx(inactive)=X1+0.5*(X2+X6-X3-X5)
Rppy(inactive)=Y1+0.5*(Y2+Y6-Y3-Y5)
Rppz(inactive)=Z1+0.5*(Z2+Z6-Z3-Z5)
prx(inactive)=Rpy*Rppz-Rppy*Rpz
pry(inactive)=Rpz*Rppx-Rppz*Rpx
prz(inactive)=Rpx*Rppy-Rppx*Rpy
nx(inactive)=prx/SQRT(prx*prx+pry*pry+prz*prz)
ny(inactive)=pry/SQRT(prx*prx+pry*pry+prz*prz)
nz(inactive)=prz/SQRT(prx*prx+pry*pry+prz*prz)
Zt1(inactive)=X1*nx+Y1*ny+Z1*nz
Zt2(inactive)=X2*nx+Y2*ny+Z2*nz
Zt3(inactive)=X3*nx+Y3*ny+Z3*nz
Zt4(inactive)=X4*nx+Y4*ny+Z4*nz
Zt5(inactive)=X5*nx+Y5*ny+Z5*nz
Zt6(inactive)=X6*nx+Y6*ny+Z6*nz
PUCKB(inactive)=SQRT(1/3)*(Zt1+Zt4-0.5*(Zt2+Zt3+Zt5+Zt6))
PUCKA(inactive)=-1*SQRT(1/3)*0.866025403*(Zt2+Zt5-Zt3-Zt6)
q2(inactive)=SQRT(PUCKA*PUCKA+PUCKB*PUCKB)
q3(inactive)=SQRT(1/6)*(Zt1-Zt2+Zt3-Zt4+Zt5-Zt6)
PHI=180+(57.29577951*(PUCKA/(SQRT(PUCKA**2)+0.0001))*ARCCOS(PUCKB/q2))
THETA=57.29577951*ARCCOS(q3/SQRT(q2*q2+q3*q3))
Qrad=SQRT(q2*q2+q3*q3)
PHI(NSteps=118,StepSize=-3.0)

#p opt=(modredundant,tight) b972/def2svp nosymm maxdisk=8GB geom=GIC scf=xqc

SAM opt

0 1
 H    -2.218357     1.114937    -1.610163
 C    -4.878326    -1.105448    -1.290041
 C    -2.763736     0.262469    -1.180633
 C    -1.988077    -1.037512    -1.364060
 C    -2.727931    -2.183970    -0.688223
 H    -5.624459    -1.472107    -2.012183
 H    -2.335782    -3.155062    -1.028695
 H    -0.974749    -0.980221    -0.940095
 H    -5.447934    -0.736305    -0.414264
 H    -2.903127     0.478940    -0.107968
 H    -1.870530    -1.232318    -2.442387
```

H	-2.519072	-2.125157	0.399187
C	-4.107095	0.076172	-1.876025
H	-4.739472	0.973048	-1.803782
H	-3.916665	-0.085646	-2.949445
O	-4.121590	-2.217687	-0.873233

```
XC(inactive)=XCntr(16,5,4,3,13,2)
YC(inactive)=YCntr(16,5,4,3,13,2)
ZC(inactive)=ZCntr(16,5,4,3,13,2)
X1(inactive)=X(16)-XC
X2(inactive)=X(5)-XC
X3(inactive)=X(4)-XC
X4(inactive)=X(3)-XC
X5(inactive)=X(13)-XC
X6(inactive)=X(2)-XC
Y1(inactive)=Y(16)-YC
Y2(inactive)=Y(5)-YC
Y3(inactive)=Y(4)-YC
Y4(inactive)=Y(3)-YC
Y5(inactive)=Y(13)-YC
Y6(inactive)=Y(2)-YC
Z1(inactive)=Z(16)-ZC
Z2(inactive)=Z(5)-ZC
Z3(inactive)=Z(4)-ZC
Z4(inactive)=Z(3)-ZC
Z5(inactive)=Z(13)-ZC
Z6(inactive)=Z(2)-ZC
Rpx(inactive)=0.866025403*(X2+X3-X5-X6)
Rpy(inactive)=0.866025403*(Y2+Y3-Y5-Y6)
Rpz(inactive)=0.866025403*(Z2+Z3-Z5-Z6)
Rppx(inactive)=X1+0.5*(X2+X6-X3-X5)
Rppy(inactive)=Y1+0.5*(Y2+Y6-Y3-Y5)
Rppz(inactive)=Z1+0.5*(Z2+Z6-Z3-Z5)
prx(inactive)=Rpy*Rppz-Rppy*Rpz
pry(inactive)=Rpz*Rppx-Rppz*Rpx
prz(inactive)=Rpx*Rppy-Rppx*Rpy
nx(inactive)=prx/SQRT(prx*prx+pry*pry+prz*prz)
ny(inactive)=pry/SQRT(prx*prx+pry*pry+prz*prz)
nz(inactive)=prz/SQRT(prx*prx+pry*pry+prz*prz)
Zt1(inactive)=X1*nx+Y1*ny+Z1*nz
Zt2(inactive)=X2*nx+Y2*ny+Z2*nz
Zt3(inactive)=X3*nx+Y3*ny+Z3*nz
Zt4(inactive)=X4*nx+Y4*ny+Z4*nz
Zt5(inactive)=X5*nx+Y5*ny+Z5*nz
Zt6(inactive)=X6*nx+Y6*ny+Z6*nz
PUCKB(inactive)=SQRT(1/3)*(Zt1+Zt4-0.5*(Zt2+Zt3+Zt5+Zt6))
PUCKA(inactive)=-1*SQRT(1/3)*0.866025403*(Zt2+Zt5-Zt3-Zt6)
q2(inactive)=SQRT(PUCKA*PUCKA+PUCKB*PUCKB)
q3(inactive)=SQRT(1/6)*(Zt1-Zt2+Zt3-Zt4+Zt5-Zt6)
PHI=180+(57.29577951*(PUCKA/(SQRT(PUCKA**2)+0.0001))*ARCCOS(PUCKB/q2))
THETA(frozen)=57.29577951*ARCCOS(q3/SQRT(q2*q2+q3*q3))
Qrad=SQRT(q2*q2+q3*q3)
PHI(NSteps=30,StepSize=-11.4453)
```

```
#p opt=(modredundant,tight) b972/def2svp nosymm maxdisk=8GB geom=GIC scf=xqc

SAM opt

0 1
 H    -2.404042     0.262587    -2.709195
 C    -4.812403    -1.180762    -1.308771
 C    -2.683130     0.218947    -1.644477
 C    -1.953114    -0.988780    -1.012876
 C    -2.836233    -2.204999    -0.739244
 H    -4.793678    -1.799664    -2.228643
 H    -2.939627    -2.826928    -1.651741
 H    -1.533005    -0.673127    -0.045392
 H    -5.861389    -1.099496    -0.992430
 H    -2.300173     1.151082    -1.209712
 H    -1.093462    -1.285029    -1.630406
 H    -2.381507    -2.840079     0.033059
 C    -4.223575     0.199808    -1.563433
 H    -4.545859     0.840496    -0.727895
 H    -4.662418     0.639450    -2.470734
 O    -4.113287    -1.839374    -0.280120

XC(inactive)=XCntr(16,5,4,3,13,2)
YC(inactive)=YCntr(16,5,4,3,13,2)
ZC(inactive)=ZCntr(16,5,4,3,13,2)
X1(inactive)=X(16)-XC
X2(inactive)=X(5)-XC
X3(inactive)=X(4)-XC
X4(inactive)=X(3)-XC
X5(inactive)=X(13)-XC
X6(inactive)=X(2)-XC
Y1(inactive)=Y(16)-YC
Y2(inactive)=Y(5)-YC
Y3(inactive)=Y(4)-YC
Y4(inactive)=Y(3)-YC
Y5(inactive)=Y(13)-YC
Y6(inactive)=Y(2)-YC
Z1(inactive)=Z(16)-ZC
Z2(inactive)=Z(5)-ZC
Z3(inactive)=Z(4)-ZC
Z4(inactive)=Z(3)-ZC
Z5(inactive)=Z(13)-ZC
Z6(inactive)=Z(2)-ZC
Rpx(inactive)=0.866025403*(X2+X3-X5-X6)
Rpy(inactive)=0.866025403*(Y2+Y3-Y5-Y6)
Rpz(inactive)=0.866025403*(Z2+Z3-Z5-Z6)
Rppx(inactive)=X1+0.5*(X2+X6-X3-X5)
Rppy(inactive)=Y1+0.5*(Y2+Y6-Y3-Y5)
Rppz(inactive)=Z1+0.5*(Z2+Z6-Z3-Z5)
prx(inactive)=Rpy*Rppz-Rppy*Rpz
pry(inactive)=Rpz*Rppx-Rppz*Rpx
prz(inactive)=Rpx*Rppy-Rppx*Rpy
nx(inactive)=prx/SQRT(prx*prx+pry*pry+prz*prz)
ny(inactive)=pry/SQRT(prx*prx+pry*pry+prz*prz)
nz(inactive)=prz/SQRT(prx*prx+pry*pry+prz*prz)
```

Zt1(inactive)=X1*nx+Y1*ny+Z1*nz
Zt2(inactive)=X2*nx+Y2*ny+Z2*nz
Zt3(inactive)=X3*nx+Y3*ny+Z3*nz
Zt4(inactive)=X4*nx+Y4*ny+Z4*nz
Zt5(inactive)=X5*nx+Y5*ny+Z5*nz
Zt6(inactive)=X6*nx+Y6*ny+Z6*nz
PUCKB(inactive)=SQRT(1/3)*(Zt1+Zt4-0.5*(Zt2+Zt3+Zt5+Zt6))
PUCKA(inactive)=-1*SQRT(1/3)*0.866025403*(Zt2+Zt5-Zt3-Zt6)
q2(inactive)=SQRT(PUCKA*PUCKA+PUCKB*PUCKB)
q3(inactive)=SQRT(1/6)*(Zt1-Zt2+Zt3-Zt4+Zt5-Zt6)
PHI=180+(57.29577951*(PUCKA/(SQRT(PUCKA**2)+0.0001))*ARCCOS(PUCKB/q2))
THETA(frozen)=57.29577951*ARCCOS(q3/SQRT(q2*q2+q3*q3))
Qrad=SQRT(q2*q2+q3*q3)
PHI(NSteps=30,StepSize=-11.4453)

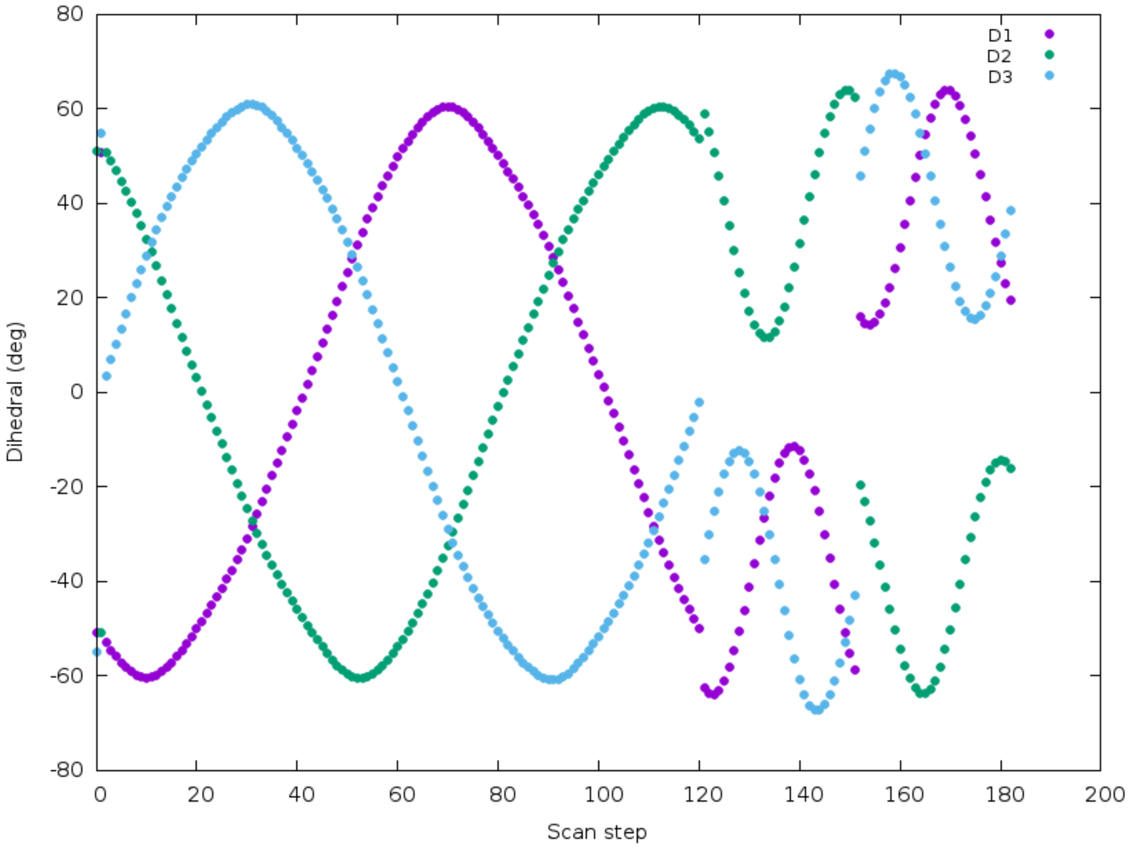

Figure S9. Evolution of the D1, D2 and D3 endocyclic dihedral angles along the scan conformational coordinate of the X=O 6-membered ring ($C_5H_{10}X$) molecule.

$C_5H_{10}S$ - Optimization $^4C_1$, optimization $^1C_4$, Scan at θ=90º (B), Scan at θ=45º and θ=135º (E)

#p opt=(modredundant,tight) b972/def2svp nosymm maxdisk=8GB geom=GIC scf=xqc

SAM opt

0 1

H	-2.188171	1.081748	-1.730567
C	-4.827145	-1.126549	-1.292515
C	-2.745652	0.268165	-1.243384
C	-1.963903	-1.044173	-1.306489
C	-2.802507	-2.183911	-0.738883
H	-5.767353	-1.338192	-1.820045
H	-2.299108	-3.151937	-0.867796
H	-1.014726	-0.969840	-0.754263
H	-5.083547	-0.919865	-0.232561
H	-2.874305	0.566557	-0.188846
H	-1.710727	-1.276592	-2.353088
H	-2.947620	-2.030321	0.350701
C	-4.118837	0.084401	-1.889708
H	-4.743647	0.981238	-1.760465
H	-4.000688	-0.072185	-2.973728
S	-4.048967	-2.294413	-1.380372

#p opt=(modredundant,tight) b972/def2svp nosymm maxdisk=8GB geom=GIC scf=xqc

SAM opt

0 1

H	-2.586808	-0.236453	-2.836242
C	-4.818426	-1.136893	-1.358206
C	-2.680830	0.098160	-1.788660
C	-2.004439	-0.922800	-0.873968
C	-2.818290	-2.209782	-0.796141
H	-4.827929	-1.614293	-2.359628
H	-2.781963	-2.736729	-1.772078
H	-1.915864	-0.505454	0.141921
H	-5.861314	-1.060887	-1.020946
H	-2.171174	1.071190	-1.730386
H	-0.982936	-1.146279	-1.217250
H	-2.406738	-2.893125	-0.040695
C	-4.163368	0.236807	-1.443080
H	-4.275783	0.736980	-0.468052
H	-4.685484	0.858925	-2.185887
S	-4.155556	-1.965236	-0.432710

#p opt=(modredundant,tight) b972/def2svp nosymm maxdisk=8GB geom=GIC scf=xqc

SAM opt

0 1

H	-2.20132200	1.09061200	-1.76313500
C	-4.82621600	-1.12969500	-1.22101600
C	-2.74543700	0.29108000	-1.24050800
C	-1.96562300	-1.02053400	-1.30850500

C	-2.79538300	-2.16265600	-0.67465300
H	-5.05931200	-1.88035700	-2.00164100
H	-2.93392800	-2.99011700	-1.39793700
H	-0.99661700	-0.91793100	-0.79971200
H	-5.77665300	-0.85637700	-0.74421300
H	-2.84056200	0.60713300	-0.19123500
H	-1.73890300	-1.25311300	-2.36064900
H	-2.28603800	-2.58322700	0.20242500
C	-4.13642700	0.10669600	-1.84584300
H	-4.74257100	1.01021500	-1.68874500
H	-4.04613600	-0.01985800	-2.93580300
S	-4.04577500	-1.71773900	-0.21083900

XC(inactive)=XCntr(16,5,4,3,13,2)
YC(inactive)=YCntr(16,5,4,3,13,2)
ZC(inactive)=ZCntr(16,5,4,3,13,2)
X1(inactive)=X(16)-XC
X2(inactive)=X(5)-XC
X3(inactive)=X(4)-XC
X4(inactive)=X(3)-XC
X5(inactive)=X(13)-XC
X6(inactive)=X(2)-XC
Y1(inactive)=Y(16)-YC
Y2(inactive)=Y(5)-YC
Y3(inactive)=Y(4)-YC
Y4(inactive)=Y(3)-YC
Y5(inactive)=Y(13)-YC
Y6(inactive)=Y(2)-YC
Z1(inactive)=Z(16)-ZC
Z2(inactive)=Z(5)-ZC
Z3(inactive)=Z(4)-ZC
Z4(inactive)=Z(3)-ZC
Z5(inactive)=Z(13)-ZC
Z6(inactive)=Z(2)-ZC
Rpx(inactive)=0.866025403*(X2+X3-X5-X6)
Rpy(inactive)=0.866025403*(Y2+Y3-Y5-Y6)
Rpz(inactive)=0.866025403*(Z2+Z3-Z5-Z6)
Rppx(inactive)=X1+0.5*(X2+X6-X3-X5)
Rppy(inactive)=Y1+0.5*(Y2+Y6-Y3-Y5)
Rppz(inactive)=Z1+0.5*(Z2+Z6-Z3-Z5)
prx(inactive)=Rpy*Rppz-Rppy*Rpz
pry(inactive)=Rpz*Rppx-Rppz*Rpx
prz(inactive)=Rpx*Rppy-Rppx*Rpy
nx(inactive)=prx/SQRT(prx*prx+pry*pry+prz*prz)
ny(inactive)=pry/SQRT(prx*prx+pry*pry+prz*prz)
nz(inactive)=prz/SQRT(prx*prx+pry*pry+prz*prz)
Zt1(inactive)=X1*nx+Y1*ny+Z1*nz
Zt2(inactive)=X2*nx+Y2*ny+Z2*nz
Zt3(inactive)=X3*nx+Y3*ny+Z3*nz
Zt4(inactive)=X4*nx+Y4*ny+Z4*nz
Zt5(inactive)=X5*nx+Y5*ny+Z5*nz
Zt6(inactive)=X6*nx+Y6*ny+Z6*nz
PUCKB(inactive)=SQRT(1/3)*(Zt1+Zt4-0.5*(Zt2+Zt3+Zt5+Zt6))
PUCKA(inactive)=-1*SQRT(1/3)*0.866025403*(Zt2+Zt5-Zt3-Zt6)
q2(inactive)=SQRT(PUCKA*PUCKA+PUCKB*PUCKB)

q3(inactive)=SQRT(1/6)*(Zt1-Zt2+Zt3-Zt4+Zt5-Zt6)
PHI=180+(57.29577951*(PUCKA/(SQRT(PUCKA**2)+0.0001))*ARCCOS(PUCKB/q2))
THETA=57.29577951*ARCCOS(q3/SQRT(q2*q2+q3*q3))
Qrad=SQRT(q2*q2+q3*q3)
PHI(NSteps=118,StepSize=-3.0)

#p opt=(modredundant,tight) b972/def2svp nosymm maxdisk=8GB geom=GIC scf=xqc

SAM opt

0 1

H	-2.21835700	1.11493700	-1.61016300
C	-4.87832600	-1.10544800	-1.29004100
C	-2.76373600	0.26246900	-1.18063300
C	-1.98807700	-1.03751200	-1.36406000
C	-2.72793100	-2.18397000	-0.68822300
H	-5.62445900	-1.47210700	-2.01218300
H	-2.33578200	-3.15506200	-1.02869500
H	-0.97474900	-0.98022100	-0.94009500
H	-5.44793400	-0.73630500	-0.41426400
H	-2.90312700	0.47894000	-0.10796800
H	-1.87053000	-1.23231800	-2.44238700
H	-2.51907200	-2.12515700	0.39918700
C	-4.10709500	0.07617200	-1.87602500
H	-4.73947200	0.97304800	-1.80378200
H	-3.91666500	-0.08564600	-2.94944500
S	-4.12159000	-2.21768700	-0.87323300

XC(inactive)=XCntr(16,5,4,3,13,2)
YC(inactive)=YCntr(16,5,4,3,13,2)
ZC(inactive)=ZCntr(16,5,4,3,13,2)
X1(inactive)=X(16)-XC
X2(inactive)=X(5)-XC
X3(inactive)=X(4)-XC
X4(inactive)=X(3)-XC
X5(inactive)=X(13)-XC
X6(inactive)=X(2)-XC
Y1(inactive)=Y(16)-YC
Y2(inactive)=Y(5)-YC
Y3(inactive)=Y(4)-YC
Y4(inactive)=Y(3)-YC
Y5(inactive)=Y(13)-YC
Y6(inactive)=Y(2)-YC
Z1(inactive)=Z(16)-ZC
Z2(inactive)=Z(5)-ZC
Z3(inactive)=Z(4)-ZC
Z4(inactive)=Z(3)-ZC
Z5(inactive)=Z(13)-ZC
Z6(inactive)=Z(2)-ZC
Rpx(inactive)=0.866025403*(X2+X3-X5-X6)
Rpy(inactive)=0.866025403*(Y2+Y3-Y5-Y6)
Rpz(inactive)=0.866025403*(Z2+Z3-Z5-Z6)
Rppx(inactive)=X1+0.5*(X2+X6-X3-X5)
Rppy(inactive)=Y1+0.5*(Y2+Y6-Y3-Y5)
Rppz(inactive)=Z1+0.5*(Z2+Z6-Z3-Z5)

```
prx(inactive)=Rpy*Rppz-Rppy*Rpz
pry(inactive)=Rpz*Rppx-Rppz*Rpx
prz(inactive)=Rpx*Rppy-Rppx*Rpy
nx(inactive)=prx/SQRT(prx*prx+pry*pry+prz*prz)
ny(inactive)=pry/SQRT(prx*prx+pry*pry+prz*prz)
nz(inactive)=prz/SQRT(prx*prx+pry*pry+prz*prz)
Zt1(inactive)=X1*nx+Y1*ny+Z1*nz
Zt2(inactive)=X2*nx+Y2*ny+Z2*nz
Zt3(inactive)=X3*nx+Y3*ny+Z3*nz
Zt4(inactive)=X4*nx+Y4*ny+Z4*nz
Zt5(inactive)=X5*nx+Y5*ny+Z5*nz
Zt6(inactive)=X6*nx+Y6*ny+Z6*nz
PUCKB(inactive)=SQRT(1/3)*(Zt1+Zt4-0.5*(Zt2+Zt3+Zt5+Zt6))
PUCKA(inactive)=-1*SQRT(1/3)*0.866025403*(Zt2+Zt5-Zt3-Zt6)
q2(inactive)=SQRT(PUCKA*PUCKA+PUCKB*PUCKB)
q3(inactive)=SQRT(1/6)*(Zt1-Zt2+Zt3-Zt4+Zt5-Zt6)
PHI=180+(57.29577951*(PUCKA/(SQRT(PUCKA**2)+0.0001))*ARCCOS(PUCKB/q2))
THETA(frozen)=57.29577951*ARCCOS(q3/SQRT(q2*q2+q3*q3))
Qrad=SQRT(q2*q2+q3*q3)
PHI(NSteps=30,StepSize=-11.4453)


#p opt=(modredundant,tight) b972/def2svp nosymm maxdisk=8GB geom=GIC scf=xqc

SAM opt

0 1
 H     -2.40404200    0.26258700   -2.70919500
 C     -4.81240300   -1.18076200   -1.30877100
 C     -2.68313000    0.21894700   -1.64447700
 C     -1.95311400   -0.98878000   -1.01287600
 C     -2.83623300   -2.20499900   -0.73924400
 H     -4.79367800   -1.79966400   -2.22864300
 H     -2.93962700   -2.82692800   -1.65174100
 H     -1.53300500   -0.67312700   -0.04539200
 H     -5.86138900   -1.09949600   -0.99243000
 H     -2.30017300    1.15108200   -1.20971200
 H     -1.09346200   -1.28502900   -1.63040600
 H     -2.38150700   -2.84007900    0.03305900
 C     -4.22357500    0.19980800   -1.56343300
 H     -4.54585900    0.84049600   -0.72789500
 H     -4.66241800    0.63945000   -2.47073400
 S     -4.11328700   -1.83937400   -0.28012000

XC(inactive)=XCntr(16,5,4,3,13,2)
YC(inactive)=YCntr(16,5,4,3,13,2)
ZC(inactive)=ZCntr(16,5,4,3,13,2)
X1(inactive)=X(16)-XC
X2(inactive)=X(5)-XC
X3(inactive)=X(4)-XC
X4(inactive)=X(3)-XC
X5(inactive)=X(13)-XC
X6(inactive)=X(2)-XC
Y1(inactive)=Y(16)-YC
Y2(inactive)=Y(5)-YC
```

```
Y3(inactive)=Y(4)-YC
Y4(inactive)=Y(3)-YC
Y5(inactive)=Y(13)-YC
Y6(inactive)=Y(2)-YC
Z1(inactive)=Z(16)-ZC
Z2(inactive)=Z(5)-ZC
Z3(inactive)=Z(4)-ZC
Z4(inactive)=Z(3)-ZC
Z5(inactive)=Z(13)-ZC
Z6(inactive)=Z(2)-ZC
Rpx(inactive)=0.866025403*(X2+X3-X5-X6)
Rpy(inactive)=0.866025403*(Y2+Y3-Y5-Y6)
Rpz(inactive)=0.866025403*(Z2+Z3-Z5-Z6)
Rppx(inactive)=X1+0.5*(X2+X6-X3-X5)
Rppy(inactive)=Y1+0.5*(Y2+Y6-Y3-Y5)
Rppz(inactive)=Z1+0.5*(Z2+Z6-Z3-Z5)
prx(inactive)=Rpy*Rppz-Rppy*Rpz
pry(inactive)=Rpz*Rppx-Rppz*Rpx
prz(inactive)=Rpx*Rppy-Rppx*Rpy
nx(inactive)=prx/SQRT(prx*prx+pry*pry+prz*prz)
ny(inactive)=pry/SQRT(prx*prx+pry*pry+prz*prz)
nz(inactive)=prz/SQRT(prx*prx+pry*pry+prz*prz)
Zt1(inactive)=X1*nx+Y1*ny+Z1*nz
Zt2(inactive)=X2*nx+Y2*ny+Z2*nz
Zt3(inactive)=X3*nx+Y3*ny+Z3*nz
Zt4(inactive)=X4*nx+Y4*ny+Z4*nz
Zt5(inactive)=X5*nx+Y5*ny+Z5*nz
Zt6(inactive)=X6*nx+Y6*ny+Z6*nz
PUCKB(inactive)=SQRT(1/3)*(Zt1+Zt4-0.5*(Zt2+Zt3+Zt5+Zt6))
PUCKA(inactive)=-1*SQRT(1/3)*0.866025403*(Zt2+Zt5-Zt3-Zt6)
q2(inactive)=SQRT(PUCKA*PUCKA+PUCKB*PUCKB)
q3(inactive)=SQRT(1/6)*(Zt1-Zt2+Zt3-Zt4+Zt5-Zt6)
PHI=180+(57.29577951*(PUCKA/(SQRT(PUCKA**2)+0.0001))*ARCCOS(PUCKB/q2))
THETA(frozen)=57.29577951*ARCCOS(q3/SQRT(q2*q2+q3*q3))
Qrad=SQRT(q2*q2+q3*q3)
PHI(NSteps=30,StepSize=-11.4453)
```

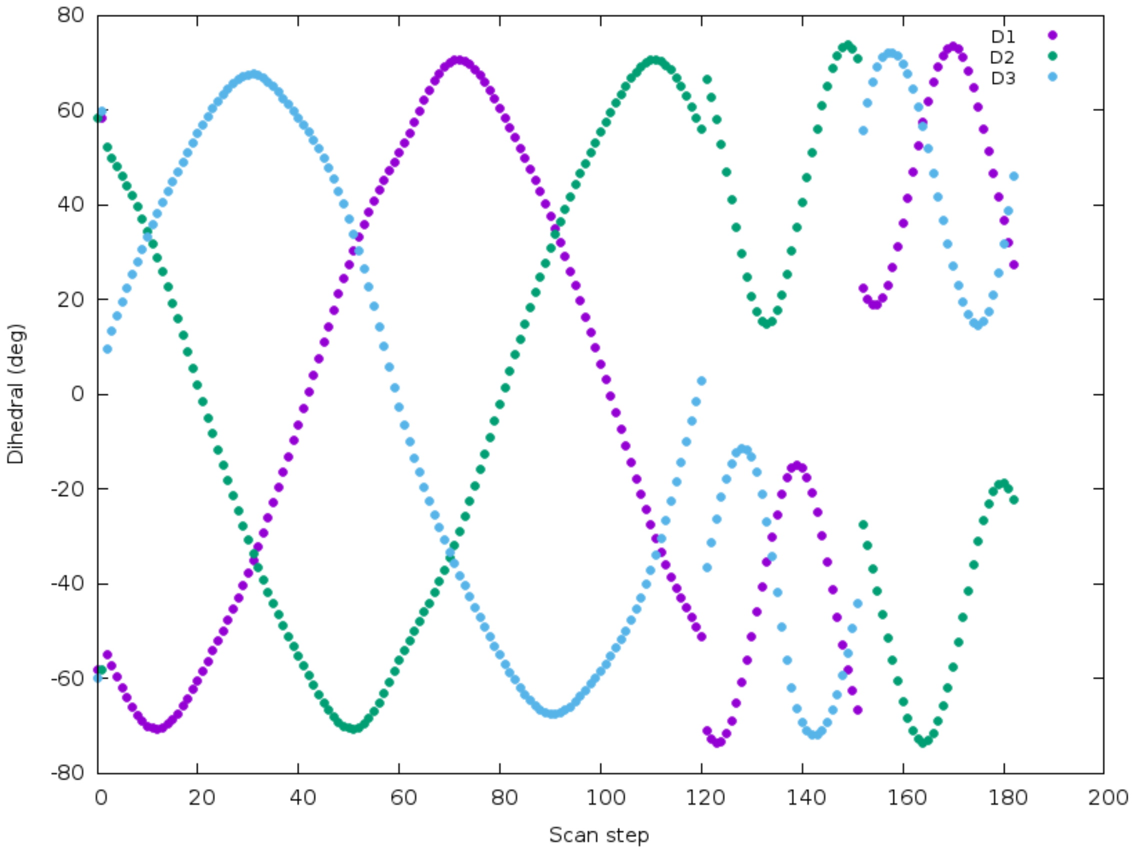

Figure S10. Evolution of the D1, D2 and D3 endocyclic dihedral angles along the scan conformational coordinate of the X=S 6-membered ring ($C_5H_{10}X$) molecule.

$C_5H_{10}SO_2$ - Optimization $^4C_1$, optimization $^1C_4$, Scan at θ=90º (B), Scan at θ=45º and θ=135º (E)

#p opt=(modredundant,tight) b972/def2svp nosymm maxdisk=8GB geom=GIC scf=xqc

SAM opt

0 1

H	-2.18647900	1.07639900	-1.74757400
C	-4.82561000	-1.12818200	-1.29147600
C	-2.74414900	0.26887500	-1.25065000
C	-1.96252600	-1.04394900	-1.30013900
C	-2.80527500	-2.18522800	-0.74191700
H	-5.77027900	-1.33607900	-1.81248900
H	-2.29936300	-3.15229300	-0.86812200
H	-1.01945900	-0.96655900	-0.73798200
H	-5.07199000	-0.93157100	-0.22739800
H	-2.86783200	0.57898400	-0.19879000
H	-1.69701200	-1.27937100	-2.34307200
H	-2.96291200	-2.03479300	0.34625600
C	-4.12185100	0.08598400	-1.88726800
H	-4.74493400	0.98294500	-1.75078400
H	-4.01234300	-0.06754000	-2.97264900
S	-4.04489000	-2.29349300	-1.39795600

O -4.89319420 -3.56587071 -0.72688601
O -3.76613271 -2.60178305 -3.01540820

#p opt=(modredundant,tight) b972/def2svp nosymm maxdisk=8GB geom=GIC scf=xqc

SAM opt

0 1
H -2.58863500 -0.26926200 -2.83789100
C -4.82203000 -1.13376400 -1.35210800
C -2.67880800 0.08853500 -1.79782600
C -2.00885000 -0.91554700 -0.85980900
C -2.81115600 -2.21090300 -0.80732700
H -4.85767000 -1.59800900 -2.35948200
H -2.75875500 -2.72228000 -1.79087400
H -1.95271900 -0.49442100 0.15661600
H -5.85727900 -1.05144600 -0.99354300
H -2.16762500 1.06183200 -1.76031000
H -0.97661800 -1.12828100 -1.17703200
H -2.40235500 -2.90215300 -0.05756600
C -4.15825100 0.23576300 -1.44215600
H -4.25787300 0.74038000 -0.46796200
H -4.68479100 0.85799100 -2.18173800
S -4.15348700 -1.98430200 -0.45299800
O -4.19358395 -1.29104747 1.06578124
O -4.94957917 -3.45187992 -0.41622442

#p opt=(modredundant,tight) b972/def2svp nosymm maxdisk=8GB geom=GIC scf=xqc

SAM opt

0 1
H -2.21063400 1.06201100 -1.78061100
C -4.97872700 -1.04883300 -1.27958000
C -2.75595400 0.26584200 -1.25246300
C -1.93896200 -1.02561200 -1.30194800
C -2.63289100 -2.24324100 -0.64468700
H -5.14169800 -1.82465500 -2.04242600
H -2.82155200 -3.03711400 -1.38234100
H -0.96759800 -0.84932600 -0.81771800
H -5.97348200 -0.72526600 -0.94623700
H -2.84540300 0.59030900 -0.20567300
H -1.71322800 -1.27142200 -2.35156500
H -2.00837600 -2.68822600 0.14114800
C -4.15738100 0.13034800 -1.85090300
H -4.70115500 1.07246100 -1.68981400
H -4.07832200 0.00839100 -2.94269100
S -4.21154000 -1.84153500 0.16550100
O -4.03413122 -0.78917876 1.45001354
O -5.03329088 -3.20436241 0.67178840

XC(inactive)=XCntr(16,5,4,3,13,2)
YC(inactive)=YCntr(16,5,4,3,13,2)

```
ZC(inactive)=ZCntr(16,5,4,3,13,2)
X1(inactive)=X(16)-XC
X2(inactive)=X(5)-XC
X3(inactive)=X(4)-XC
X4(inactive)=X(3)-XC
X5(inactive)=X(13)-XC
X6(inactive)=X(2)-XC
Y1(inactive)=Y(16)-YC
Y2(inactive)=Y(5)-YC
Y3(inactive)=Y(4)-YC
Y4(inactive)=Y(3)-YC
Y5(inactive)=Y(13)-YC
Y6(inactive)=Y(2)-YC
Z1(inactive)=Z(16)-ZC
Z2(inactive)=Z(5)-ZC
Z3(inactive)=Z(4)-ZC
Z4(inactive)=Z(3)-ZC
Z5(inactive)=Z(13)-ZC
Z6(inactive)=Z(2)-ZC
Rpx(inactive)=0.866025403*(X2+X3-X5-X6)
Rpy(inactive)=0.866025403*(Y2+Y3-Y5-Y6)
Rpz(inactive)=0.866025403*(Z2+Z3-Z5-Z6)
Rppx(inactive)=X1+0.5*(X2+X6-X3-X5)
Rppy(inactive)=Y1+0.5*(Y2+Y6-Y3-Y5)
Rppz(inactive)=Z1+0.5*(Z2+Z6-Z3-Z5)
prx(inactive)=Rpy*Rppz-Rppy*Rpz
pry(inactive)=Rpz*Rppx-Rppz*Rpx
prz(inactive)=Rpx*Rppy-Rppx*Rpy
nx(inactive)=prx/SQRT(prx*prx+pry*pry+prz*prz)
ny(inactive)=pry/SQRT(prx*prx+pry*pry+prz*prz)
nz(inactive)=prz/SQRT(prx*prx+pry*pry+prz*prz)
Zt1(inactive)=X1*nx+Y1*ny+Z1*nz
Zt2(inactive)=X2*nx+Y2*ny+Z2*nz
Zt3(inactive)=X3*nx+Y3*ny+Z3*nz
Zt4(inactive)=X4*nx+Y4*ny+Z4*nz
Zt5(inactive)=X5*nx+Y5*ny+Z5*nz
Zt6(inactive)=X6*nx+Y6*ny+Z6*nz
PUCKB(inactive)=SQRT(1/3)*(Zt1+Zt4-0.5*(Zt2+Zt3+Zt5+Zt6))
PUCKA(inactive)=-1*SQRT(1/3)*0.866025403*(Zt2+Zt5-Zt3-Zt6)
q2(inactive)=SQRT(PUCKA*PUCKA+PUCKB*PUCKB)
q3(inactive)=SQRT(1/6)*(Zt1-Zt2+Zt3-Zt4+Zt5-Zt6)
PHI=180+(57.29577951*(PUCKA/(SQRT(PUCKA**2)+0.0001))*ARCCOS(PUCKB/q2))
THETA=57.29577951*ARCCOS(q3/SQRT(q2*q2+q3*q3))
Qrad=SQRT(q2*q2+q3*q3)
PHI(NSteps=118,StepSize=-3.0)

#p opt=(modredundant,tight) b972/def2svp nosymm maxdisk=8GB geom=GIC scf=xqc

SAM opt

0 1
 H    -2.24886700    1.08913000   -1.54051600
 C    -5.06439700   -0.97822000   -1.34197700
 C    -2.79613600    0.21719300   -1.15378500
 C    -1.96257300   -1.04490700   -1.36277400
```

C	-2.51373500	-2.25936500	-0.62892300
H	-5.79339200	-1.28010800	-2.10536300
H	-2.05576900	-3.18412800	-1.00452500
H	-0.92831800	-0.88921700	-1.01938700
H	-5.63653200	-0.55558500	-0.50255200
H	-2.95818300	0.39966900	-0.07831100
H	-1.90250800	-1.25118300	-2.44347200
H	-2.25355800	-2.18044400	0.43768900
C	-4.12579900	0.09344400	-1.89169300
H	-4.66907400	1.05047600	-1.87678600
H	-3.90577500	-0.11602000	-2.95086200
S	-4.32228700	-2.53660000	-0.71877600
O	-4.92390876	-2.89640158	0.79697269
O	-4.66017072	-3.79392262	-1.76466052

```
XC(inactive)=XCntr(16,5,4,3,13,2)
YC(inactive)=YCntr(16,5,4,3,13,2)
ZC(inactive)=ZCntr(16,5,4,3,13,2)
X1(inactive)=X(16)-XC
X2(inactive)=X(5)-XC
X3(inactive)=X(4)-XC
X4(inactive)=X(3)-XC
X5(inactive)=X(13)-XC
X6(inactive)=X(2)-XC
Y1(inactive)=Y(16)-YC
Y2(inactive)=Y(5)-YC
Y3(inactive)=Y(4)-YC
Y4(inactive)=Y(3)-YC
Y5(inactive)=Y(13)-YC
Y6(inactive)=Y(2)-YC
Z1(inactive)=Z(16)-ZC
Z2(inactive)=Z(5)-ZC
Z3(inactive)=Z(4)-ZC
Z4(inactive)=Z(3)-ZC
Z5(inactive)=Z(13)-ZC
Z6(inactive)=Z(2)-ZC
Rpx(inactive)=0.866025403*(X2+X3-X5-X6)
Rpy(inactive)=0.866025403*(Y2+Y3-Y5-Y6)
Rpz(inactive)=0.866025403*(Z2+Z3-Z5-Z6)
Rppx(inactive)=X1+0.5*(X2+X6-X3-X5)
Rppy(inactive)=Y1+0.5*(Y2+Y6-Y3-Y5)
Rppz(inactive)=Z1+0.5*(Z2+Z6-Z3-Z5)
prx(inactive)=Rpy*Rppz-Rppy*Rpz
pry(inactive)=Rpz*Rppx-Rppz*Rpx
prz(inactive)=Rpx*Rppy-Rppx*Rpy
nx(inactive)=prx/SQRT(prx*prx+pry*pry+prz*prz)
ny(inactive)=pry/SQRT(prx*prx+pry*pry+prz*prz)
nz(inactive)=prz/SQRT(prx*prx+pry*pry+prz*prz)
Zt1(inactive)=X1*nx+Y1*ny+Z1*nz
Zt2(inactive)=X2*nx+Y2*ny+Z2*nz
Zt3(inactive)=X3*nx+Y3*ny+Z3*nz
Zt4(inactive)=X4*nx+Y4*ny+Z4*nz
Zt5(inactive)=X5*nx+Y5*ny+Z5*nz
Zt6(inactive)=X6*nx+Y6*ny+Z6*nz
PUCKB(inactive)=SQRT(1/3)*(Zt1+Zt4-0.5*(Zt2+Zt3+Zt5+Zt6))
```

```
PUCKA(inactive)=-1*SQRT(1/3)*0.866025403*(Zt2+Zt5-Zt3-Zt6)
q2(inactive)=SQRT(PUCKA*PUCKA+PUCKB*PUCKB)
q3(inactive)=SQRT(1/6)*(Zt1-Zt2+Zt3-Zt4+Zt5-Zt6)
PHI=180+(57.29577951*(PUCKA/(SQRT(PUCKA**2)+0.0001))*ARCCOS(PUCKB/q2))
THETA(frozen)=57.29577951*ARCCOS(q3/SQRT(q2*q2+q3*q3))
Qrad=SQRT(q2*q2+q3*q3)
PHI(NSteps=30,StepSize=-11.4453)


#p opt=(modredundant,tight) b972/def2svp nosymm maxdisk=8GB geom=GIC scf=xqc

SAM opt

0 1
 H        -2.40404200    0.26258700   -2.70919500
 C        -4.81240300   -1.18076200   -1.30877100
 C        -2.68313000    0.21894700   -1.64447700
 C        -1.95311400   -0.98878000   -1.01287600
 C        -2.83623300   -2.20499900   -0.73924400
 H        -4.79367800   -1.79966400   -2.22864300
 H        -2.93962700   -2.82692800   -1.65174100
 H        -1.53300500   -0.67312700   -0.04539200
 H        -5.86138900   -1.09949600   -0.99243000
 H        -2.30017300    1.15108200   -1.20971200
 H        -1.09346200   -1.28502900   -1.63040600
 H        -2.38150700   -2.84007900    0.03305900
 C        -4.22357500    0.19980800   -1.56343300
 H        -4.54585900    0.84049600   -0.72789500
 H        -4.66241800    0.63945000   -2.47073400
 S        -4.11328700   -1.83937400   -0.28012000
 O        -3.96919240   -0.81329050    1.02957058
 O        -4.94415628   -3.21361998    0.17814219

XC(inactive)=XCntr(16,5,4,3,13,2)
YC(inactive)=YCntr(16,5,4,3,13,2)
ZC(inactive)=ZCntr(16,5,4,3,13,2)
X1(inactive)=X(16)-XC
X2(inactive)=X(5)-XC
X3(inactive)=X(4)-XC
X4(inactive)=X(3)-XC
X5(inactive)=X(13)-XC
X6(inactive)=X(2)-XC
Y1(inactive)=Y(16)-YC
Y2(inactive)=Y(5)-YC
Y3(inactive)=Y(4)-YC
Y4(inactive)=Y(3)-YC
Y5(inactive)=Y(13)-YC
Y6(inactive)=Y(2)-YC
Z1(inactive)=Z(16)-ZC
Z2(inactive)=Z(5)-ZC
Z3(inactive)=Z(4)-ZC
Z4(inactive)=Z(3)-ZC
Z5(inactive)=Z(13)-ZC
Z6(inactive)=Z(2)-ZC
Rpx(inactive)=0.866025403*(X2+X3-X5-X6)
```

Rpy(inactive)=0.866025403*(Y2+Y3-Y5-Y6)
Rpz(inactive)=0.866025403*(Z2+Z3-Z5-Z6)
Rppx(inactive)=X1+0.5*(X2+X6-X3-X5)
Rppy(inactive)=Y1+0.5*(Y2+Y6-Y3-Y5)
Rppz(inactive)=Z1+0.5*(Z2+Z6-Z3-Z5)
prx(inactive)=Rpy*Rppz-Rppy*Rpz
pry(inactive)=Rpz*Rppx-Rppz*Rpx
prz(inactive)=Rpx*Rppy-Rppx*Rpy
nx(inactive)=prx/SQRT(prx*prx+pry*pry+prz*prz)
ny(inactive)=pry/SQRT(prx*prx+pry*pry+prz*prz)
nz(inactive)=prz/SQRT(prx*prx+pry*pry+prz*prz)
Zt1(inactive)=X1*nx+Y1*ny+Z1*nz
Zt2(inactive)=X2*nx+Y2*ny+Z2*nz
Zt3(inactive)=X3*nx+Y3*ny+Z3*nz
Zt4(inactive)=X4*nx+Y4*ny+Z4*nz
Zt5(inactive)=X5*nx+Y5*ny+Z5*nz
Zt6(inactive)=X6*nx+Y6*ny+Z6*nz
PUCKB(inactive)=SQRT(1/3)*(Zt1+Zt4-0.5*(Zt2+Zt3+Zt5+Zt6))
PUCKA(inactive)=-1*SQRT(1/3)*0.866025403*(Zt2+Zt5-Zt3-Zt6)
q2(inactive)=SQRT(PUCKA*PUCKA+PUCKB*PUCKB)
q3(inactive)=SQRT(1/6)*(Zt1-Zt2+Zt3-Zt4+Zt5-Zt6)
PHI=180+(57.29577951*(PUCKA/(SQRT(PUCKA**2)+0.0001))*ARCCOS(PUCKB/q2))
THETA(frozen)=57.29577951*ARCCOS(q3/SQRT(q2*q2+q3*q3))
Qrad=SQRT(q2*q2+q3*q3)
PHI(NSteps=30,StepSize=-11.4453)

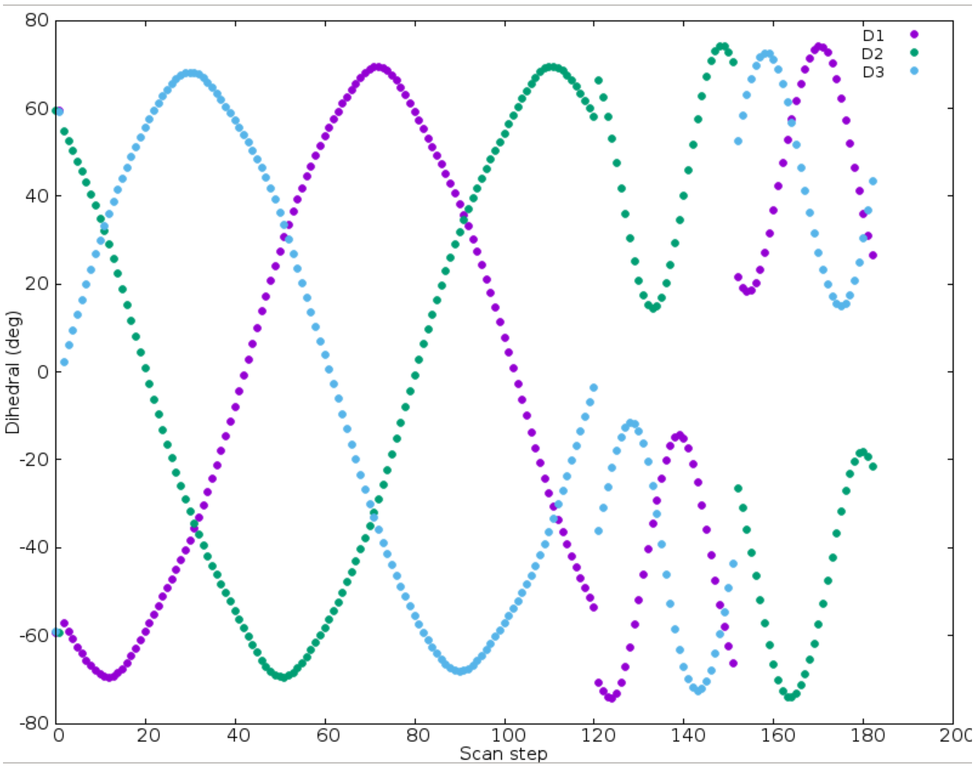

Figure S11. Evolution of the D1, D2 and D3 endocyclic dihedral angles along the scan conformational coordinate of the X=SO_2 6-membered ring ($C_5H_{10}X$) molecule.

$C_5H_{10}NH$ - Optimization $^4C_1$, optimization $^1C_4$, Scan at θ=90° (B), Scan at θ=45° and θ=135° (E)

#p opt=(modredundant,tight) b972/def2svp nosymm maxdisk=8GB scf=xqc

SAM opt

0 1

H	-2.21835700	1.11493700	-1.61016300
C	-4.87832600	-1.10544800	-1.29004100
C	-2.76373600	0.26246900	-1.18063300
C	-1.98807700	-1.03751200	-1.36406000
C	-2.72793100	-2.18397000	-0.68822300
H	-5.62445900	-1.47210700	-2.01218300
H	-2.33578200	-3.15506200	-1.02869500
H	-0.97474900	-0.98022100	-0.94009500
H	-5.44793400	-0.73630500	-0.41426400
H	-2.90312700	0.47894000	-0.10796800
H	-1.87053000	-1.23231800	-2.44238700
H	-2.51907200	-2.12515700	0.39918700
C	-4.10709500	0.07617200	-1.87602500
H	-4.73947200	0.97304800	-1.80378200
H	-3.91666500	-0.08564600	-2.94944500
N	-4.12159000	-2.21768700	-0.87323300
H	-4.51391437	-2.51467960	-0.00267170

#p opt=(modredundant,tight) b972/def2svp nosymm maxdisk=8GB scf=xqc

SAM opt

0 1

H	-2.40404200	0.26258700	-2.70919500
C	-4.81240300	-1.18076200	-1.30877100
C	-2.68313000	0.21894700	-1.64447700
C	-1.95311400	-0.98878000	-1.01287600
C	-2.83623300	-2.20499900	-0.73924400
H	-4.79367800	-1.79966400	-2.22864300
H	-2.93962700	-2.82692800	-1.65174100
H	-1.53300500	-0.67312700	-0.04539200
H	-5.86138900	-1.09949600	-0.99243000
H	-2.30017300	1.15108200	-1.20971200
H	-1.09346200	-1.28502900	-1.63040600
H	-2.38150700	-2.84007900	0.03305900
C	-4.22357500	0.19980800	-1.56343300
H	-4.54585900	0.84049600	-0.72789500
H	-4.66241800	0.63945000	-2.47073400
N	-4.11328700	-1.83937400	-0.28012000
H	-4.61656300	-2.65928957	-0.00726157

#p opt=(modredundant,tight) b972/def2svp nosymm maxdisk=8GB geom=GIC scf=xqc

SAM opt

0 1

H	-2.20132200	1.09061200	-1.76313500
C	-4.82621600	-1.12969500	-1.22101600
C	-2.74543700	0.29108000	-1.24050800
C	-1.96562300	-1.02053400	-1.30850500

C	-2.79538300	-2.16265600	-0.67465300
H	-5.05931200	-1.88035700	-2.00164100
H	-2.93392800	-2.99011700	-1.39793700
H	-0.99661700	-0.91793100	-0.79971200
H	-5.77665300	-0.85637700	-0.74421300
H	-2.84056200	0.60713300	-0.19123500
H	-1.73890300	-1.25311300	-2.36064900
H	-2.28603800	-2.58322700	0.20242500
C	-4.13642700	0.10669600	-1.84584300
H	-4.74257100	1.01021500	-1.68874500
H	-4.04613600	-0.01985800	-2.93580300
N	-4.04577500	-1.71773900	-0.21083900
H	-4.54434437	-2.49738194	0.16808772

```
XC(inactive)=XCntr(16,5,4,3,13,2)
YC(inactive)=YCntr(16,5,4,3,13,2)
ZC(inactive)=ZCntr(16,5,4,3,13,2)
X1(inactive)=X(16)-XC
X2(inactive)=X(5)-XC
X3(inactive)=X(4)-XC
X4(inactive)=X(3)-XC
X5(inactive)=X(13)-XC
X6(inactive)=X(2)-XC
Y1(inactive)=Y(16)-YC
Y2(inactive)=Y(5)-YC
Y3(inactive)=Y(4)-YC
Y4(inactive)=Y(3)-YC
Y5(inactive)=Y(13)-YC
Y6(inactive)=Y(2)-YC
Z1(inactive)=Z(16)-ZC
Z2(inactive)=Z(5)-ZC
Z3(inactive)=Z(4)-ZC
Z4(inactive)=Z(3)-ZC
Z5(inactive)=Z(13)-ZC
Z6(inactive)=Z(2)-ZC
Rpx(inactive)=0.866025403*(X2+X3-X5-X6)
Rpy(inactive)=0.866025403*(Y2+Y3-Y5-Y6)
Rpz(inactive)=0.866025403*(Z2+Z3-Z5-Z6)
Rppx(inactive)=X1+0.5*(X2+X6-X3-X5)
Rppy(inactive)=Y1+0.5*(Y2+Y6-Y3-Y5)
Rppz(inactive)=Z1+0.5*(Z2+Z6-Z3-Z5)
prx(inactive)=Rpy*Rppz-Rppy*Rpz
pry(inactive)=Rpz*Rppx-Rppz*Rpx
prz(inactive)=Rpx*Rppy-Rppx*Rpy
nx(inactive)=prx/SQRT(prx*prx+pry*pry+prz*prz)
ny(inactive)=pry/SQRT(prx*prx+pry*pry+prz*prz)
nz(inactive)=prz/SQRT(prx*prx+pry*pry+prz*prz)
Zt1(inactive)=X1*nx+Y1*ny+Z1*nz
Zt2(inactive)=X2*nx+Y2*ny+Z2*nz
Zt3(inactive)=X3*nx+Y3*ny+Z3*nz
Zt4(inactive)=X4*nx+Y4*ny+Z4*nz
Zt5(inactive)=X5*nx+Y5*ny+Z5*nz
Zt6(inactive)=X6*nx+Y6*ny+Z6*nz
PUCKB(inactive)=SQRT(1/3)*(Zt1+Zt4-0.5*(Zt2+Zt3+Zt5+Zt6))
PUCKA(inactive)=-1*SQRT(1/3)*0.866025403*(Zt2+Zt5-Zt3-Zt6)
```

q2(inactive)=SQRT(PUCKA*PUCKA+PUCKB*PUCKB)
q3(inactive)=SQRT(1/6)*(Zt1-Zt2+Zt3-Zt4+Zt5-Zt6)
PHI=180+(57.29577951*(PUCKA/(SQRT(PUCKA**2)+0.0001))*ARCCOS(PUCKB/q2))
THETA=57.29577951*ARCCOS(q3/SQRT(q2*q2+q3*q3))
Qrad=SQRT(q2*q2+q3*q3)
PHI(NSteps=118,StepSize=-3.0)

#p opt=(modredundant,tight) b972/def2svp nosymm maxdisk=8GB geom=GIC scf=xqc

SAM opt

0 1

H	-2.21835700	1.11493700	-1.61016300
C	-4.87832600	-1.10544800	-1.29004100
C	-2.76373600	0.26246900	-1.18063300
C	-1.98807700	-1.03751200	-1.36406000
C	-2.72793100	-2.18397000	-0.68822300
H	-5.62445900	-1.47210700	-2.01218300
H	-2.33578200	-3.15506200	-1.02869500
H	-0.97474900	-0.98022100	-0.94009500
H	-5.44793400	-0.73630500	-0.41426400
H	-2.90312700	0.47894000	-0.10796800
H	-1.87053000	-1.23231800	-2.44238700
H	-2.51907200	-2.12515700	0.39918700
C	-4.10709500	0.07617200	-1.87602500
H	-4.73947200	0.97304800	-1.80378200
H	-3.91666500	-0.08564600	-2.94944500
N	-4.12159000	-2.21768700	-0.87323300
H	-4.51391437	-2.51467960	-0.00267170

XC(inactive)=XCntr(16,5,4,3,13,2)
YC(inactive)=YCntr(16,5,4,3,13,2)
ZC(inactive)=ZCntr(16,5,4,3,13,2)
X1(inactive)=X(16)-XC
X2(inactive)=X(5)-XC
X3(inactive)=X(4)-XC
X4(inactive)=X(3)-XC
X5(inactive)=X(13)-XC
X6(inactive)=X(2)-XC
Y1(inactive)=Y(16)-YC
Y2(inactive)=Y(5)-YC
Y3(inactive)=Y(4)-YC
Y4(inactive)=Y(3)-YC
Y5(inactive)=Y(13)-YC
Y6(inactive)=Y(2)-YC
Z1(inactive)=Z(16)-ZC
Z2(inactive)=Z(5)-ZC
Z3(inactive)=Z(4)-ZC
Z4(inactive)=Z(3)-ZC
Z5(inactive)=Z(13)-ZC
Z6(inactive)=Z(2)-ZC
Rpx(inactive)=0.866025403*(X2+X3-X5-X6)
Rpy(inactive)=0.866025403*(Y2+Y3-Y5-Y6)
Rpz(inactive)=0.866025403*(Z2+Z3-Z5-Z6)
Rppx(inactive)=X1+0.5*(X2+X6-X3-X5)

Rppy(inactive)=Y1+0.5*(Y2+Y6-Y3-Y5)
Rppz(inactive)=Z1+0.5*(Z2+Z6-Z3-Z5)
prx(inactive)=Rpy*Rppz-Rppy*Rpz
pry(inactive)=Rpz*Rppx-Rppz*Rpx
prz(inactive)=Rpx*Rppy-Rppx*Rpy
nx(inactive)=prx/SQRT(prx*prx+pry*pry+prz*prz)
ny(inactive)=pry/SQRT(prx*prx+pry*pry+prz*prz)
nz(inactive)=prz/SQRT(prx*prx+pry*pry+prz*prz)
Zt1(inactive)=X1*nx+Y1*ny+Z1*nz
Zt2(inactive)=X2*nx+Y2*ny+Z2*nz
Zt3(inactive)=X3*nx+Y3*ny+Z3*nz
Zt4(inactive)=X4*nx+Y4*ny+Z4*nz
Zt5(inactive)=X5*nx+Y5*ny+Z5*nz
Zt6(inactive)=X6*nx+Y6*ny+Z6*nz
PUCKB(inactive)=SQRT(1/3)*(Zt1+Zt4-0.5*(Zt2+Zt3+Zt5+Zt6))
PUCKA(inactive)=-1*SQRT(1/3)*0.866025403*(Zt2+Zt5-Zt3-Zt6)
q2(inactive)=SQRT(PUCKA*PUCKA+PUCKB*PUCKB)
q3(inactive)=SQRT(1/6)*(Zt1-Zt2+Zt3-Zt4+Zt5-Zt6)
PHI=180+(57.29577951*(PUCKA/(SQRT(PUCKA**2)+0.0001))*ARCCOS(PUCKB/q2))
THETA(frozen)=57.29577951*ARCCOS(q3/SQRT(q2*q2+q3*q3))
Qrad=SQRT(q2*q2+q3*q3)
PHI(NSteps=30,StepSize=-11.4453)

#p opt=(modredundant,tight) b972/def2svp nosymm maxdisk=8GB geom=GIC scf=xqc

SAM opt

0 1
```
 H   -2.40404200    0.26258700   -2.70919500
 C   -4.81240300   -1.18076200   -1.30877100
 C   -2.68313000    0.21894700   -1.64447700
 C   -1.95311400   -0.98878000   -1.01287600
 C   -2.83623300   -2.20499900   -0.73924400
 H   -4.79367800   -1.79966400   -2.22864300
 H   -2.93962700   -2.82692800   -1.65174100
 H   -1.53300500   -0.67312700   -0.04539200
 H   -5.86138900   -1.09949600   -0.99243000
 H   -2.30017300    1.15108200   -1.20971200
 H   -1.09346200   -1.28502900   -1.63040600
 H   -2.38150700   -2.84007900    0.03305900
 C   -4.22357500    0.19980800   -1.56343300
 H   -4.54585900    0.84049600   -0.72789500
 H   -4.66241800    0.63945000   -2.47073400
 N   -4.11328700   -1.83937400   -0.28012000
 H   -4.61656300   -2.65928957   -0.00726157
```

XC(inactive)=XCntr(16,5,4,3,13,2)
YC(inactive)=YCntr(16,5,4,3,13,2)
ZC(inactive)=ZCntr(16,5,4,3,13,2)
X1(inactive)=X(16)-XC
X2(inactive)=X(5)-XC
X3(inactive)=X(4)-XC
X4(inactive)=X(3)-XC
X5(inactive)=X(13)-XC

```
X6(inactive)=X(2)-XC
Y1(inactive)=Y(16)-YC
Y2(inactive)=Y(5)-YC
Y3(inactive)=Y(4)-YC
Y4(inactive)=Y(3)-YC
Y5(inactive)=Y(13)-YC
Y6(inactive)=Y(2)-YC
Z1(inactive)=Z(16)-ZC
Z2(inactive)=Z(5)-ZC
Z3(inactive)=Z(4)-ZC
Z4(inactive)=Z(3)-ZC
Z5(inactive)=Z(13)-ZC
Z6(inactive)=Z(2)-ZC
Rpx(inactive)=0.866025403*(X2+X3-X5-X6)
Rpy(inactive)=0.866025403*(Y2+Y3-Y5-Y6)
Rpz(inactive)=0.866025403*(Z2+Z3-Z5-Z6)
Rppx(inactive)=X1+0.5*(X2+X6-X3-X5)
Rppy(inactive)=Y1+0.5*(Y2+Y6-Y3-Y5)
Rppz(inactive)=Z1+0.5*(Z2+Z6-Z3-Z5)
prx(inactive)=Rpy*Rppz-Rppy*Rpz
pry(inactive)=Rpz*Rppx-Rppz*Rpx
prz(inactive)=Rpx*Rppy-Rppx*Rpy
nx(inactive)=prx/SQRT(prx*prx+pry*pry+prz*prz)
ny(inactive)=pry/SQRT(prx*prx+pry*pry+prz*prz)
nz(inactive)=prz/SQRT(prx*prx+pry*pry+prz*prz)
Zt1(inactive)=X1*nx+Y1*ny+Z1*nz
Zt2(inactive)=X2*nx+Y2*ny+Z2*nz
Zt3(inactive)=X3*nx+Y3*ny+Z3*nz
Zt4(inactive)=X4*nx+Y4*ny+Z4*nz
Zt5(inactive)=X5*nx+Y5*ny+Z5*nz
Zt6(inactive)=X6*nx+Y6*ny+Z6*nz
PUCKB(inactive)=SQRT(1/3)*(Zt1+Zt4-0.5*(Zt2+Zt3+Zt5+Zt6))
PUCKA(inactive)=-1*SQRT(1/3)*0.866025403*(Zt2+Zt5-Zt3-Zt6)
q2(inactive)=SQRT(PUCKA*PUCKA+PUCKB*PUCKB)
q3(inactive)=SQRT(1/6)*(Zt1-Zt2+Zt3-Zt4+Zt5-Zt6)
PHI=180+(57.29577951*(PUCKA/(SQRT(PUCKA**2)+0.0001))*ARCCOS(PUCKB/q2))
THETA(frozen)=57.29577951*ARCCOS(q3/SQRT(q2*q2+q3*q3))
Qrad=SQRT(q2*q2+q3*q3)
PHI(NSteps=30,StepSize=-11.4453)
```

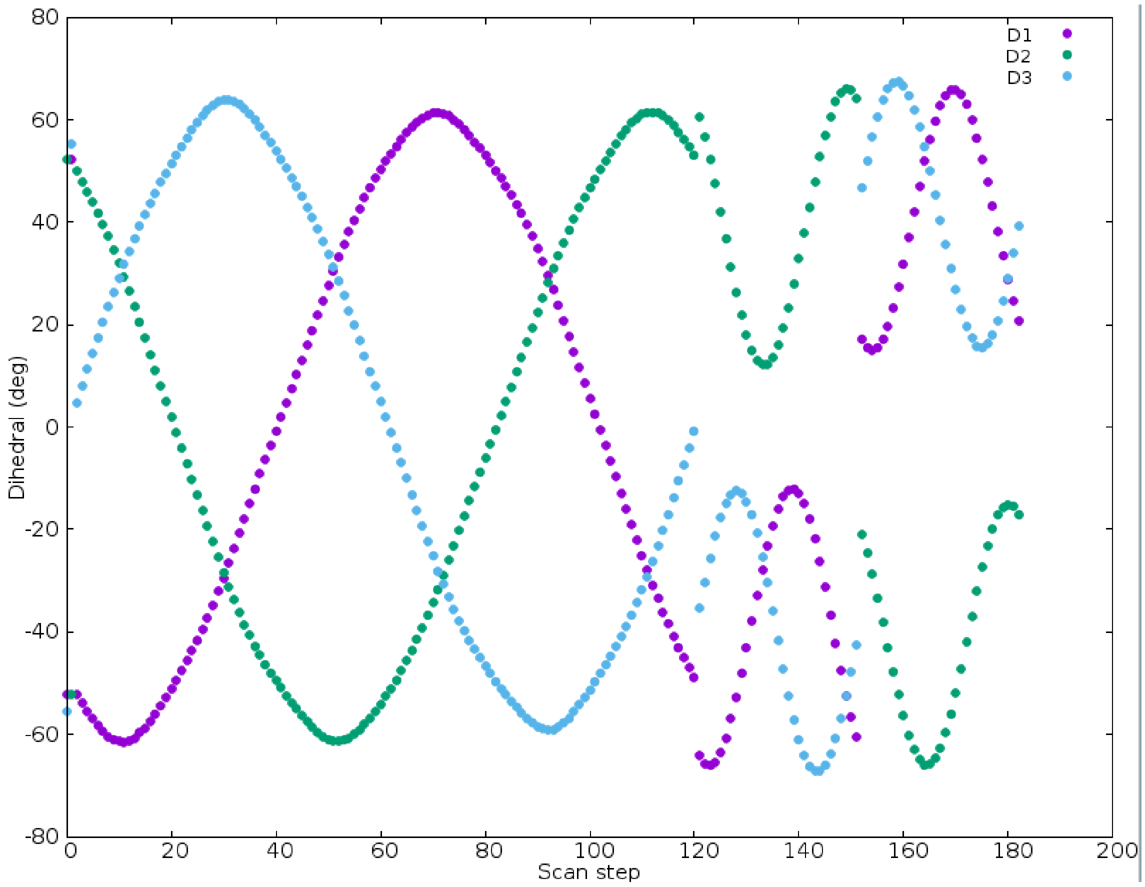

Figure S12. Evolution of the D1, D2 and D3 endocyclic dihedral angles along the scan conformational coordinate of the X=NH 6-membered ring ($C_5H_{10}X$) molecule.

$C_5H_{10}BH$ - Optimization $^4C_1$, optimization $^1C_4$, Scan at θ=90º (B), Scan at θ=45º and θ=135º (E)

#p opt=(modredundant,tight) b972/def2svp nosymm maxdisk=8GB scf=xqc

SAM opt

0 1

H	-2.21835700	1.11493700	-1.61016300
C	-4.87832600	-1.10544800	-1.29004100
C	-2.76373600	0.26246900	-1.18063300
C	-1.98807700	-1.03751200	-1.36406000
C	-2.72793100	-2.18397000	-0.68822300
H	-5.62445900	-1.47210700	-2.01218300
H	-2.33578200	-3.15506200	-1.02869500
H	-0.97474900	-0.98022100	-0.94009500
H	-5.44793400	-0.73630500	-0.41426400
H	-2.90312700	0.47894000	-0.10796800
H	-1.87053000	-1.23231800	-2.44238700
H	-2.51907200	-2.12515700	0.39918700
C	-4.10709500	0.07617200	-1.87602500
H	-4.73947200	0.97304800	-1.80378200
H	-3.91666500	-0.08564600	-2.94944500

```
 B            -4.12159000   -2.21768700   -0.87323300
 H            -4.51391437   -2.51467960   -0.00267170

#p opt=(modredundant,tight) b972/def2svp nosymm maxdisk=8GB scf=xqc

SAM opt

0 1
 H            -2.40404200    0.26258700   -2.70919500
 C            -4.81240300   -1.18076200   -1.30877100
 C            -2.68313000    0.21894700   -1.64447700
 C            -1.95311400   -0.98878000   -1.01287600
 C            -2.83623300   -2.20499900   -0.73924400
 H            -4.79367800   -1.79966400   -2.22864300
 H            -2.93962700   -2.82692800   -1.65174100
 H            -1.53300500   -0.67312700   -0.04539200
 H            -5.86138900   -1.09949600   -0.99243000
 H            -2.30017300    1.15108200   -1.20971200
 H            -1.09346200   -1.28502900   -1.63040600
 H            -2.38150700   -2.84007900    0.03305900
 C            -4.22357500    0.19980800   -1.56343300
 H            -4.54585900    0.84049600   -0.72789500
 H            -4.66241800    0.63945000   -2.47073400
 B            -4.11328700   -1.83937400   -0.28012000
 H            -4.61656300   -2.65928957   -0.00726157

#p opt=(modredundant,tight) b972/def2svp nosymm maxdisk=8GB geom=GIC scf=xqc

SAM opt

0 1
 H            -2.20132200    1.09061200   -1.76313500
 C            -4.82621600   -1.12969500   -1.22101600
 C            -2.74543700    0.29108000   -1.24050800
 C            -1.96562300   -1.02053400   -1.30850500
 C            -2.79538300   -2.16265600   -0.67465300
 H            -5.05931200   -1.88035700   -2.00164100
 H            -2.93392800   -2.99011700   -1.39793700
 H            -0.99661700   -0.91793100   -0.79971200
 H            -5.77665300   -0.85637700   -0.74421300
 H            -2.84056200    0.60713300   -0.19123500
 H            -1.73890300   -1.25311300   -2.36064900
 H            -2.28603800   -2.58322700    0.20242500
 C            -4.13642700    0.10669600   -1.84584300
 H            -4.74257100    1.01021500   -1.68874500
 H            -4.04613600   -0.01985800   -2.93580300
 B            -4.04577500   -1.71773900   -0.21083900
 H            -4.54434437   -2.49738194    0.16808772

XC(inactive)=XCntr(16,5,4,3,13,2)
YC(inactive)=YCntr(16,5,4,3,13,2)
ZC(inactive)=ZCntr(16,5,4,3,13,2)
X1(inactive)=X(16)-XC
X2(inactive)=X(5)-XC
X3(inactive)=X(4)-XC
```

X4(inactive)=X(3)-XC
X5(inactive)=X(13)-XC
X6(inactive)=X(2)-XC
Y1(inactive)=Y(16)-YC
Y2(inactive)=Y(5)-YC
Y3(inactive)=Y(4)-YC
Y4(inactive)=Y(3)-YC
Y5(inactive)=Y(13)-YC
Y6(inactive)=Y(2)-YC
Z1(inactive)=Z(16)-ZC
Z2(inactive)=Z(5)-ZC
Z3(inactive)=Z(4)-ZC
Z4(inactive)=Z(3)-ZC
Z5(inactive)=Z(13)-ZC
Z6(inactive)=Z(2)-ZC
Rpx(inactive)=0.866025403*(X2+X3-X5-X6)
Rpy(inactive)=0.866025403*(Y2+Y3-Y5-Y6)
Rpz(inactive)=0.866025403*(Z2+Z3-Z5-Z6)
Rppx(inactive)=X1+0.5*(X2+X6-X3-X5)
Rppy(inactive)=Y1+0.5*(Y2+Y6-Y3-Y5)
Rppz(inactive)=Z1+0.5*(Z2+Z6-Z3-Z5)
prx(inactive)=Rpy*Rppz-Rppy*Rpz
pry(inactive)=Rpz*Rppx-Rppz*Rpx
prz(inactive)=Rpx*Rppy-Rppx*Rpy
nx(inactive)=prx/SQRT(prx*prx+pry*pry+prz*prz)
ny(inactive)=pry/SQRT(prx*prx+pry*pry+prz*prz)
nz(inactive)=prz/SQRT(prx*prx+pry*pry+prz*prz)
Zt1(inactive)=X1*nx+Y1*ny+Z1*nz
Zt2(inactive)=X2*nx+Y2*ny+Z2*nz
Zt3(inactive)=X3*nx+Y3*ny+Z3*nz
Zt4(inactive)=X4*nx+Y4*ny+Z4*nz
Zt5(inactive)=X5*nx+Y5*ny+Z5*nz
Zt6(inactive)=X6*nx+Y6*ny+Z6*nz
PUCKB(inactive)=SQRT(1/3)*(Zt1+Zt4-0.5*(Zt2+Zt3+Zt5+Zt6))
PUCKA(inactive)=-1*SQRT(1/3)*0.866025403*(Zt2+Zt5-Zt3-Zt6)
q2(inactive)=SQRT(PUCKA*PUCKA+PUCKB*PUCKB)
q3(inactive)=SQRT(1/6)*(Zt1-Zt2+Zt3-Zt4+Zt5-Zt6)
PHI=180+(57.29577951*(PUCKA/(SQRT(PUCKA**2)+0.0001))*ARCCOS(PUCKB/q2))
THETA(frozen)=57.29577951*ARCCOS(q3/SQRT(q2*q2+q3*q3))
Qrad=SQRT(q2*q2+q3*q3)
PHI(NSteps=118,StepSize=-3.0)

#p opt=(modredundant,tight) b972/def2svp nosymm maxdisk=8GB geom=GIC scf=xqc

SAM opt

0 1

H	-2.21835700	1.11493700	-1.61016300
C	-4.87832600	-1.10544800	-1.29004100
C	-2.76373600	0.26246900	-1.18063300
C	-1.98807700	-1.03751200	-1.36406000
C	-2.72793100	-2.18397000	-0.68822300
H	-5.62445900	-1.47210700	-2.01218300
H	-2.33578200	-3.15506200	-1.02869500
H	-0.97474900	-0.98022100	-0.94009500

H	-5.44793400	-0.73630500	-0.41426400
H	-2.90312700	0.47894000	-0.10796800
H	-1.87053000	-1.23231800	-2.44238700
H	-2.51907200	-2.12515700	0.39918700
C	-4.10709500	0.07617200	-1.87602500
H	-4.73947200	0.97304800	-1.80378200
H	-3.91666500	-0.08564600	-2.94944500
B	-4.12159000	-2.21768700	-0.87323300
H	-4.51391437	-2.51467960	-0.00267170

```
XC(inactive)=XCntr(16,5,4,3,13,2)
YC(inactive)=YCntr(16,5,4,3,13,2)
ZC(inactive)=ZCntr(16,5,4,3,13,2)
X1(inactive)=X(16)-XC
X2(inactive)=X(5)-XC
X3(inactive)=X(4)-XC
X4(inactive)=X(3)-XC
X5(inactive)=X(13)-XC
X6(inactive)=X(2)-XC
Y1(inactive)=Y(16)-YC
Y2(inactive)=Y(5)-YC
Y3(inactive)=Y(4)-YC
Y4(inactive)=Y(3)-YC
Y5(inactive)=Y(13)-YC
Y6(inactive)=Y(2)-YC
Z1(inactive)=Z(16)-ZC
Z2(inactive)=Z(5)-ZC
Z3(inactive)=Z(4)-ZC
Z4(inactive)=Z(3)-ZC
Z5(inactive)=Z(13)-ZC
Z6(inactive)=Z(2)-ZC
Rpx(inactive)=0.866025403*(X2+X3-X5-X6)
Rpy(inactive)=0.866025403*(Y2+Y3-Y5-Y6)
Rpz(inactive)=0.866025403*(Z2+Z3-Z5-Z6)
Rppx(inactive)=X1+0.5*(X2+X6-X3-X5)
Rppy(inactive)=Y1+0.5*(Y2+Y6-Y3-Y5)
Rppz(inactive)=Z1+0.5*(Z2+Z6-Z3-Z5)
prx(inactive)=Rpy*Rppz-Rppy*Rpz
pry(inactive)=Rpz*Rppx-Rppz*Rpx
prz(inactive)=Rpx*Rppy-Rppx*Rpy
nx(inactive)=prx/SQRT(prx*prx+pry*pry+prz*prz)
ny(inactive)=pry/SQRT(prx*prx+pry*pry+prz*prz)
nz(inactive)=prz/SQRT(prx*prx+pry*pry+prz*prz)
Zt1(inactive)=X1*nx+Y1*ny+Z1*nz
Zt2(inactive)=X2*nx+Y2*ny+Z2*nz
Zt3(inactive)=X3*nx+Y3*ny+Z3*nz
Zt4(inactive)=X4*nx+Y4*ny+Z4*nz
Zt5(inactive)=X5*nx+Y5*ny+Z5*nz
Zt6(inactive)=X6*nx+Y6*ny+Z6*nz
PUCKB(inactive)=SQRT(1/3)*(Zt1+Zt4-0.5*(Zt2+Zt3+Zt5+Zt6))
PUCKA(inactive)=-1*SQRT(1/3)*0.866025403*(Zt2+Zt5-Zt3-Zt6)
q2(inactive)=SQRT(PUCKA*PUCKA+PUCKB*PUCKB)
q3(inactive)=SQRT(1/6)*(Zt1-Zt2+Zt3-Zt4+Zt5-Zt6)
PHI=180+(57.29577951*(PUCKA/(SQRT(PUCKA**2)+0.0001))*ARCCOS(PUCKB/q2))
THETA(frozen)=57.29577951*ARCCOS(q3/SQRT(q2*q2+q3*q3))
```

```
Qrad=SQRT(q2*q2+q3*q3)
PHI(NSteps=30,StepSize=-11.4453)


#p opt=(modredundant,tight) b972/def2svp nosymm maxdisk=8GB geom=GIC scf=xqc

SAM opt

0 1
 H        -2.40404200    0.26258700   -2.70919500
 C        -4.81240300   -1.18076200   -1.30877100
 C        -2.68313000    0.21894700   -1.64447700
 C        -1.95311400   -0.98878000   -1.01287600
 C        -2.83623300   -2.20499900   -0.73924400
 H        -4.79367800   -1.79966400   -2.22864300
 H        -2.93962700   -2.82692800   -1.65174100
 H        -1.53300500   -0.67312700   -0.04539200
 H        -5.86138900   -1.09949600   -0.99243000
 H        -2.30017300    1.15108200   -1.20971200
 H        -1.09346200   -1.28502900   -1.63040600
 H        -2.38150700   -2.84007900    0.03305900
 C        -4.22357500    0.19980800   -1.56343300
 H        -4.54585900    0.84049600   -0.72789500
 H        -4.66241800    0.63945000   -2.47073400
 B        -4.11328700   -1.83937400   -0.28012000
 H        -4.61656300   -2.65928957   -0.00726157

XC(inactive)=XCntr(16,5,4,3,13,2)
YC(inactive)=YCntr(16,5,4,3,13,2)
ZC(inactive)=ZCntr(16,5,4,3,13,2)
X1(inactive)=X(16)-XC
X2(inactive)=X(5)-XC
X3(inactive)=X(4)-XC
X4(inactive)=X(3)-XC
X5(inactive)=X(13)-XC
X6(inactive)=X(2)-XC
Y1(inactive)=Y(16)-YC
Y2(inactive)=Y(5)-YC
Y3(inactive)=Y(4)-YC
Y4(inactive)=Y(3)-YC
Y5(inactive)=Y(13)-YC
Y6(inactive)=Y(2)-YC
Z1(inactive)=Z(16)-ZC
Z2(inactive)=Z(5)-ZC
Z3(inactive)=Z(4)-ZC
Z4(inactive)=Z(3)-ZC
Z5(inactive)=Z(13)-ZC
Z6(inactive)=Z(2)-ZC
Rpx(inactive)=0.866025403*(X2+X3-X5-X6)
Rpy(inactive)=0.866025403*(Y2+Y3-Y5-Y6)
Rpz(inactive)=0.866025403*(Z2+Z3-Z5-Z6)
Rppx(inactive)=X1+0.5*(X2+X6-X3-X5)
Rppy(inactive)=Y1+0.5*(Y2+Y6-Y3-Y5)
Rppz(inactive)=Z1+0.5*(Z2+Z6-Z3-Z5)
prx(inactive)=Rpy*Rppz-Rppy*Rpz
```

pry(inactive)=Rpz*Rppx-Rppz*Rpx
prz(inactive)=Rpx*Rppy-Rppx*Rpy
nx(inactive)=prx/SQRT(prx*prx+pry*pry+prz*prz)
ny(inactive)=pry/SQRT(prx*prx+pry*pry+prz*prz)
nz(inactive)=prz/SQRT(prx*prx+pry*pry+prz*prz)
Zt1(inactive)=X1*nx+Y1*ny+Z1*nz
Zt2(inactive)=X2*nx+Y2*ny+Z2*nz
Zt3(inactive)=X3*nx+Y3*ny+Z3*nz
Zt4(inactive)=X4*nx+Y4*ny+Z4*nz
Zt5(inactive)=X5*nx+Y5*ny+Z5*nz
Zt6(inactive)=X6*nx+Y6*ny+Z6*nz
PUCKB(inactive)=SQRT(1/3)*(Zt1+Zt4-0.5*(Zt2+Zt3+Zt5+Zt6))
PUCKA(inactive)=-1*SQRT(1/3)*0.866025403*(Zt2+Zt5-Zt3-Zt6)
q2(inactive)=SQRT(PUCKA*PUCKA+PUCKB*PUCKB)
q3(inactive)=SQRT(1/6)*(Zt1-Zt2+Zt3-Zt4+Zt5-Zt6)
PHI=180+(57.29577951*(PUCKA/(SQRT(PUCKA**2)+0.0001))*ARCCOS(PUCKB/q2))
THETA(frozen)=57.29577951*ARCCOS(q3/SQRT(q2*q2+q3*q3))
Qrad=SQRT(q2*q2+q3*q3)
PHI(NSteps=30,StepSize=-11.4453)

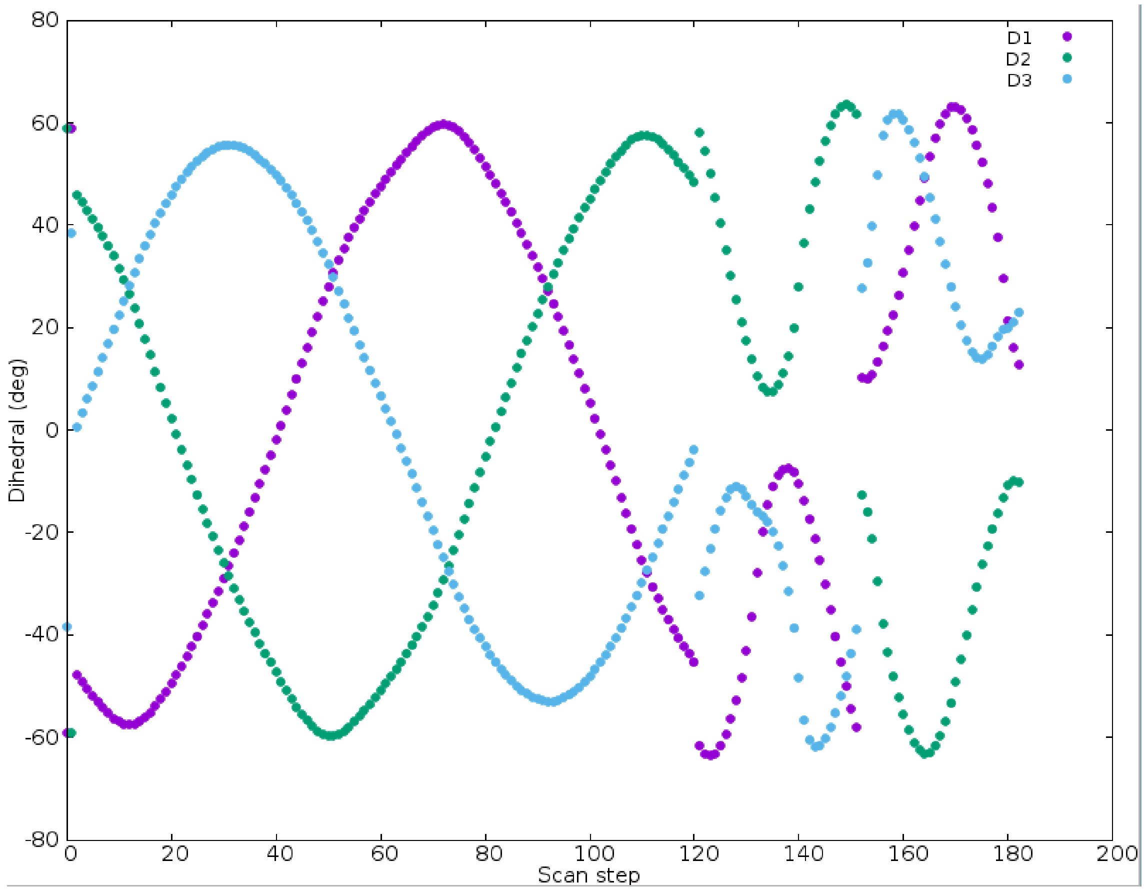

Figure S13. Evolution of the D1, D2 and D3 endocyclic dihedral angles along the scan conformational coordinate of the X=BH 6-membered ring ($C_5H_{10}X$) molecule.

$C_5H_{10}PH$ - Optimization $^4C_1$, optimization $^1C_4$, Scan at θ=90º (B), Scan at θ=45º and θ=135º (E)

#p opt=(modredundant,tight) b972/def2svp nosymm maxdisk=8GB scf=xqc

SAM opt

0 1

H	-2.21835700	1.11493700	-1.61016300
C	-4.87832600	-1.10544800	-1.29004100
C	-2.76373600	0.26246900	-1.18063300
C	-1.98807700	-1.03751200	-1.36406000
C	-2.72793100	-2.18397000	-0.68822300
H	-5.62445900	-1.47210700	-2.01218300
H	-2.33578200	-3.15506200	-1.02869500
H	-0.97474900	-0.98022100	-0.94009500
H	-5.44793400	-0.73630500	-0.41426400
H	-2.90312700	0.47894000	-0.10796800
H	-1.87053000	-1.23231800	-2.44238700
H	-2.51907200	-2.12515700	0.39918700
C	-4.10709500	0.07617200	-1.87602500
H	-4.73947200	0.97304800	-1.80378200
H	-3.91666500	-0.08564600	-2.94944500
P	-4.12159000	-2.21768700	-0.87323300
H	-4.51391437	-2.51467960	-0.00267170

#p opt=(modredundant,tight) b972/def2svp nosymm maxdisk=8GB scf=xqc

SAM opt

0 1

H	-2.40404200	0.26258700	-2.70919500
C	-4.81240300	-1.18076200	-1.30877100
C	-2.68313000	0.21894700	-1.64447700
C	-1.95311400	-0.98878000	-1.01287600
C	-2.83623300	-2.20499900	-0.73924400
H	-4.79367800	-1.79966400	-2.22864300
H	-2.93962700	-2.82692800	-1.65174100
H	-1.53300500	-0.67312700	-0.04539200
H	-5.86138900	-1.09949600	-0.99243000
H	-2.30017300	1.15108200	-1.20971200
H	-1.09346200	-1.28502900	-1.63040600
H	-2.38150700	-2.84007900	0.03305900
C	-4.22357500	0.19980800	-1.56343300
H	-4.54585900	0.84049600	-0.72789500
H	-4.66241800	0.63945000	-2.47073400
P	-4.11328700	-1.83937400	-0.28012000
H	-4.61656300	-2.65928957	-0.00726157

#p opt=(modredundant,tight) b972/def2svp nosymm maxdisk=8GB geom=GIC scf=xqc

SAM opt

0 1

H	-2.20132200	1.09061200	-1.76313500
C	-4.82621600	-1.12969500	-1.22101600
C	-2.74543700	0.29108000	-1.24050800
C	-1.96562300	-1.02053400	-1.30850500

C	-2.79538300	-2.16265600	-0.67465300
H	-5.05931200	-1.88035700	-2.00164100
H	-2.93392800	-2.99011700	-1.39793700
H	-0.99661700	-0.91793100	-0.79971200
H	-5.77665300	-0.85637700	-0.74421300
H	-2.84056200	0.60713300	-0.19123500
H	-1.73890300	-1.25311300	-2.36064900
H	-2.28603800	-2.58322700	0.20242500
C	-4.13642700	0.10669600	-1.84584300
H	-4.74257100	1.01021500	-1.68874500
H	-4.04613600	-0.01985800	-2.93580300
P	-4.04577500	-1.71773900	-0.21083900
H	-4.54434437	-2.49738194	0.16808772

```
XC(inactive)=XCntr(16,5,4,3,13,2)
YC(inactive)=YCntr(16,5,4,3,13,2)
ZC(inactive)=ZCntr(16,5,4,3,13,2)
X1(inactive)=X(16)-XC
X2(inactive)=X(5)-XC
X3(inactive)=X(4)-XC
X4(inactive)=X(3)-XC
X5(inactive)=X(13)-XC
X6(inactive)=X(2)-XC
Y1(inactive)=Y(16)-YC
Y2(inactive)=Y(5)-YC
Y3(inactive)=Y(4)-YC
Y4(inactive)=Y(3)-YC
Y5(inactive)=Y(13)-YC
Y6(inactive)=Y(2)-YC
Z1(inactive)=Z(16)-ZC
Z2(inactive)=Z(5)-ZC
Z3(inactive)=Z(4)-ZC
Z4(inactive)=Z(3)-ZC
Z5(inactive)=Z(13)-ZC
Z6(inactive)=Z(2)-ZC
Rpx(inactive)=0.866025403*(X2+X3-X5-X6)
Rpy(inactive)=0.866025403*(Y2+Y3-Y5-Y6)
Rpz(inactive)=0.866025403*(Z2+Z3-Z5-Z6)
Rppx(inactive)=X1+0.5*(X2+X6-X3-X5)
Rppy(inactive)=Y1+0.5*(Y2+Y6-Y3-Y5)
Rppz(inactive)=Z1+0.5*(Z2+Z6-Z3-Z5)
prx(inactive)=Rpy*Rppz-Rppy*Rpz
pry(inactive)=Rpz*Rppx-Rppz*Rpx
prz(inactive)=Rpx*Rppy-Rppx*Rpy
nx(inactive)=prx/SQRT(prx*prx+pry*pry+prz*prz)
ny(inactive)=pry/SQRT(prx*prx+pry*pry+prz*prz)
nz(inactive)=prz/SQRT(prx*prx+pry*pry+prz*prz)
Zt1(inactive)=X1*nx+Y1*ny+Z1*nz
Zt2(inactive)=X2*nx+Y2*ny+Z2*nz
Zt3(inactive)=X3*nx+Y3*ny+Z3*nz
Zt4(inactive)=X4*nx+Y4*ny+Z4*nz
Zt5(inactive)=X5*nx+Y5*ny+Z5*nz
Zt6(inactive)=X6*nx+Y6*ny+Z6*nz
PUCKB(inactive)=SQRT(1/3)*(Zt1+Zt4-0.5*(Zt2+Zt3+Zt5+Zt6))
PUCKA(inactive)=-1*SQRT(1/3)*0.866025403*(Zt2+Zt5-Zt3-Zt6)
```

```
q2(inactive)=SQRT(PUCKA*PUCKA+PUCKB*PUCKB)
q3(inactive)=SQRT(1/6)*(Zt1-Zt2+Zt3-Zt4+Zt5-Zt6)
PHI=180+(57.29577951*(PUCKA/(SQRT(PUCKA**2)+0.0001))*ARCCOS(PUCKB/q2))
THETA=57.29577951*ARCCOS(q3/SQRT(q2*q2+q3*q3))
Qrad=SQRT(q2*q2+q3*q3)
PHI(NSteps=118,StepSize=-3.0)

#p opt=(modredundant,tight) b972/def2svp nosymm maxdisk=8GB geom=GIC scf=xqc

SAM opt

0 1
 H              -2.21835700    1.11493700   -1.61016300
 C              -4.87832600   -1.10544800   -1.29004100
 C              -2.76373600    0.26246900   -1.18063300
 C              -1.98807700   -1.03751200   -1.36406000
 C              -2.72793100   -2.18397000   -0.68822300
 H              -5.62445900   -1.47210700   -2.01218300
 H              -2.33578200   -3.15506200   -1.02869500
 H              -0.97474900   -0.98022100   -0.94009500
 H              -5.44793400   -0.73630500   -0.41426400
 H              -2.90312700    0.47894000   -0.10796800
 H              -1.87053000   -1.23231800   -2.44238700
 H              -2.51907200   -2.12515700    0.39918700
 C              -4.10709500    0.07617200   -1.87602500
 H              -4.73947200    0.97304800   -1.80378200
 H              -3.91666500   -0.08564600   -2.94944500
 P              -4.12159000   -2.21768700   -0.87323300
 H              -4.51391437   -2.51467960   -0.00267170

XC(inactive)=XCntr(16,5,4,3,13,2)
YC(inactive)=YCntr(16,5,4,3,13,2)
ZC(inactive)=ZCntr(16,5,4,3,13,2)
X1(inactive)=X(16)-XC
X2(inactive)=X(5)-XC
X3(inactive)=X(4)-XC
X4(inactive)=X(3)-XC
X5(inactive)=X(13)-XC
X6(inactive)=X(2)-XC
Y1(inactive)=Y(16)-YC
Y2(inactive)=Y(5)-YC
Y3(inactive)=Y(4)-YC
Y4(inactive)=Y(3)-YC
Y5(inactive)=Y(13)-YC
Y6(inactive)=Y(2)-YC
Z1(inactive)=Z(16)-ZC
Z2(inactive)=Z(5)-ZC
Z3(inactive)=Z(4)-ZC
Z4(inactive)=Z(3)-ZC
Z5(inactive)=Z(13)-ZC
Z6(inactive)=Z(2)-ZC
Rpx(inactive)=0.866025403*(X2+X3-X5-X6)
Rpy(inactive)=0.866025403*(Y2+Y3-Y5-Y6)
Rpz(inactive)=0.866025403*(Z2+Z3-Z5-Z6)
Rppx(inactive)=X1+0.5*(X2+X6-X3-X5)
```

Rppy(inactive)=Y1+0.5*(Y2+Y6-Y3-Y5)
Rppz(inactive)=Z1+0.5*(Z2+Z6-Z3-Z5)
prx(inactive)=Rpy*Rppz-Rppy*Rpz
pry(inactive)=Rpz*Rppx-Rppz*Rpx
prz(inactive)=Rpx*Rppy-Rppx*Rpy
nx(inactive)=prx/SQRT(prx*prx+pry*pry+prz*prz)
ny(inactive)=pry/SQRT(prx*prx+pry*pry+prz*prz)
nz(inactive)=prz/SQRT(prx*prx+pry*pry+prz*prz)
Zt1(inactive)=X1*nx+Y1*ny+Z1*nz
Zt2(inactive)=X2*nx+Y2*ny+Z2*nz
Zt3(inactive)=X3*nx+Y3*ny+Z3*nz
Zt4(inactive)=X4*nx+Y4*ny+Z4*nz
Zt5(inactive)=X5*nx+Y5*ny+Z5*nz
Zt6(inactive)=X6*nx+Y6*ny+Z6*nz
PUCKB(inactive)=SQRT(1/3)*(Zt1+Zt4-0.5*(Zt2+Zt3+Zt5+Zt6))
PUCKA(inactive)=-1*SQRT(1/3)*0.866025403*(Zt2+Zt5-Zt3-Zt6)
q2(inactive)=SQRT(PUCKA*PUCKA+PUCKB*PUCKB)
q3(inactive)=SQRT(1/6)*(Zt1-Zt2+Zt3-Zt4+Zt5-Zt6)
PHI=180+(57.29577951*(PUCKA/(SQRT(PUCKA**2)+0.0001))*ARCCOS(PUCKB/q2))
THETA(frozen)=57.29577951*ARCCOS(q3/SQRT(q2*q2+q3*q3))
Qrad=SQRT(q2*q2+q3*q3)
PHI(NSteps=30,StepSize=-11.4453)

#p opt=(modredundant,tight) b972/def2svp nosymm maxdisk=8GB geom=GIC scf=xqc

SAM opt

0 1

H	-2.40404200	0.26258700	-2.70919500
C	-4.81240300	-1.18076200	-1.30877100
C	-2.68313000	0.21894700	-1.64447700
C	-1.95311400	-0.98878000	-1.01287600
C	-2.83623300	-2.20499900	-0.73924400
H	-4.79367800	-1.79966400	-2.22864300
H	-2.93962700	-2.82692800	-1.65174100
H	-1.53300500	-0.67312700	-0.04539200
H	-5.86138900	-1.09949600	-0.99243000
H	-2.30017300	1.15108200	-1.20971200
H	-1.09346200	-1.28502900	-1.63040600
H	-2.38150700	-2.84007900	0.03305900
C	-4.22357500	0.19980800	-1.56343300
H	-4.54585900	0.84049600	-0.72789500
H	-4.66241800	0.63945000	-2.47073400
P	-4.11328700	-1.83937400	-0.28012000
H	-4.61656300	-2.65928957	-0.00726157

XC(inactive)=XCntr(16,5,4,3,13,2)
YC(inactive)=YCntr(16,5,4,3,13,2)
ZC(inactive)=ZCntr(16,5,4,3,13,2)
X1(inactive)=X(16)-XC
X2(inactive)=X(5)-XC
X3(inactive)=X(4)-XC
X4(inactive)=X(3)-XC
X5(inactive)=X(13)-XC

```
X6(inactive)=X(2)-XC
Y1(inactive)=Y(16)-YC
Y2(inactive)=Y(5)-YC
Y3(inactive)=Y(4)-YC
Y4(inactive)=Y(3)-YC
Y5(inactive)=Y(13)-YC
Y6(inactive)=Y(2)-YC
Z1(inactive)=Z(16)-ZC
Z2(inactive)=Z(5)-ZC
Z3(inactive)=Z(4)-ZC
Z4(inactive)=Z(3)-ZC
Z5(inactive)=Z(13)-ZC
Z6(inactive)=Z(2)-ZC
Rpx(inactive)=0.866025403*(X2+X3-X5-X6)
Rpy(inactive)=0.866025403*(Y2+Y3-Y5-Y6)
Rpz(inactive)=0.866025403*(Z2+Z3-Z5-Z6)
Rppx(inactive)=X1+0.5*(X2+X6-X3-X5)
Rppy(inactive)=Y1+0.5*(Y2+Y6-Y3-Y5)
Rppz(inactive)=Z1+0.5*(Z2+Z6-Z3-Z5)
prx(inactive)=Rpy*Rppz-Rppy*Rpz
pry(inactive)=Rpz*Rppx-Rppz*Rpx
prz(inactive)=Rpx*Rppy-Rppx*Rpy
nx(inactive)=prx/SQRT(prx*prx+pry*pry+prz*prz)
ny(inactive)=pry/SQRT(prx*prx+pry*pry+prz*prz)
nz(inactive)=prz/SQRT(prx*prx+pry*pry+prz*prz)
Zt1(inactive)=X1*nx+Y1*ny+Z1*nz
Zt2(inactive)=X2*nx+Y2*ny+Z2*nz
Zt3(inactive)=X3*nx+Y3*ny+Z3*nz
Zt4(inactive)=X4*nx+Y4*ny+Z4*nz
Zt5(inactive)=X5*nx+Y5*ny+Z5*nz
Zt6(inactive)=X6*nx+Y6*ny+Z6*nz
PUCKB(inactive)=SQRT(1/3)*(Zt1+Zt4-0.5*(Zt2+Zt3+Zt5+Zt6))
PUCKA(inactive)=-1*SQRT(1/3)*0.866025403*(Zt2+Zt5-Zt3-Zt6)
q2(inactive)=SQRT(PUCKA*PUCKA+PUCKB*PUCKB)
q3(inactive)=SQRT(1/6)*(Zt1-Zt2+Zt3-Zt4+Zt5-Zt6)
PHI=180+(57.29577951*(PUCKA/(SQRT(PUCKA**2)+0.0001))*ARCCOS(PUCKB/q2))
THETA(frozen)=57.29577951*ARCCOS(q3/SQRT(q2*q2+q3*q3))
Qrad=SQRT(q2*q2+q3*q3)
PHI(NSteps=30,StepSize=-11.4453)
```

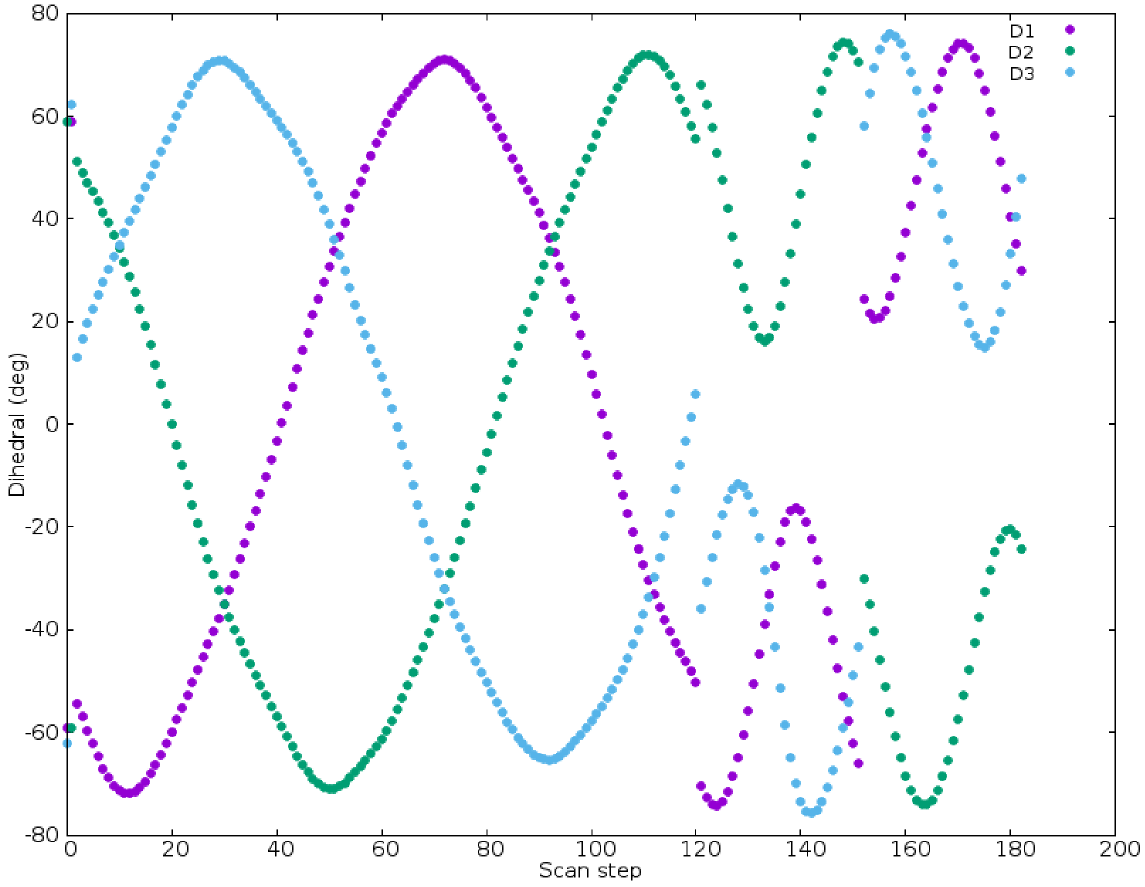

Figure S14. Evolution of the D1, D2 and D3 endocyclic dihedral angles along the scan conformational coordinate of the X=PH 6-membered ring ($C_5H_{10}X$) molecule.

7-membered rings

C_7H_{14} – TC/C plane and TB/B plane

%mem=8GB
#p opt=(modredundant,newton) b972/def2svp nosymm maxdisk=8GB scf=xqc geom=GIC

SAM dihedral scan

0 1

C	-1.845876	1.528675	0.018960
C	-2.208676	0.845024	-1.149494
C	-1.868696	0.789078	1.211181
H	-1.556776	-0.041043	-1.297788
C	-3.673092	0.417252	-1.231869
H	-1.990355	1.543141	-1.970158
C	-3.272673	0.454704	1.716388
H	-1.357312	1.421119	1.951028
H	-1.268308	-0.138223	1.108761
H	-4.301110	1.276053	-0.946723
C	-4.051910	-0.835145	-0.441131

H	-3.896643	0.222541	-2.293120
C	-3.929261	-0.783489	1.100880
H	-3.215874	0.298838	2.805510
H	-3.898187	1.349097	1.573451
H	-3.431601	-1.662359	-0.822000
H	-5.083148	-1.109120	-0.708689
H	-4.929649	-0.885471	1.547894
H	-3.372967	-1.670236	1.442436
H	-4.249771	1.869124	0.379434
H	-1.978155	3.191658	-0.347016

```
XC(inactive)=XCntr(1,2,5,11,13,7,3)
YC(inactive)=YCntr(1,2,5,11,13,7,3)
ZC(inactive)=ZCntr(1,2,5,11,13,7,3)
X1(inactive)=X(1)-XC
X2(inactive)=X(2)-XC
X3(inactive)=X(5)-XC
X4(inactive)=X(11)-XC
X5(inactive)=X(13)-XC
X6(inactive)=X(7)-XC
X7(inactive)=X(3)-XC
Y1(inactive)=Y(1)-YC
Y2(inactive)=Y(2)-YC
Y3(inactive)=Y(5)-YC
Y4(inactive)=Y(11)-YC
Y5(inactive)=Y(13)-YC
Y6(inactive)=Y(7)-YC
Y7(inactive)=Y(3)-YC
Z1(inactive)=Z(1)-ZC
Z2(inactive)=Z(2)-ZC
Z3(inactive)=Z(5)-ZC
Z4(inactive)=Z(11)-ZC
Z5(inactive)=Z(13)-ZC
Z6(inactive)=Z(7)-ZC
Z7(inactive)=Z(3)-ZC
Rpx(inactive)=0.781831482*(X2-X7)+0.974927912*(X3-X6)+0.433883739*(X4-X5)
Rpy(inactive)=0.781831482*(Y2-Y7)+0.974927912*(Y3-Y6)+0.433883739*(Y4-Y5)
Rpz(inactive)=0.781831482*(Z2-Z7)+0.974927912*(Z3-Z6)+0.433883739*(Z4-Z5)
Rppx(inactive)=X1+0.623489801*(X2+X7)-0.222520934*(X3+X6)-0.900968867*(X4+X5)
Rppy(inactive)=Y1+0.623489801*(Y2+Y7)-0.222520934*(Y3+Y6)-0.900968867*(Y4+Y5)
Rppz(inactive)=Z1+0.623489801*(Z2+Z7)-0.222520934*(Z3+Z6)-0.900968867*(Z4+Z5)
prx(inactive)=Rpy*Rppz-Rppy*Rpz
pry(inactive)=Rpz*Rppx-Rppz*Rpx
prz(inactive)=Rpx*Rppy-Rppx*Rpy
nx(inactive)=prx/SQRT(prx*prx+pry*pry+prz*prz)
ny(inactive)=pry/SQRT(prx*prx+pry*pry+prz*prz)
nz(inactive)=prz/SQRT(prx*prx+pry*pry+prz*prz)
Zt1(inactive)=X1*nx+Y1*ny+Z1*nz
Zt2(inactive)=X2*nx+Y2*ny+Z2*nz
Zt3(inactive)=X3*nx+Y3*ny+Z3*nz
Zt4(inactive)=X4*nx+Y4*ny+Z4*nz
Zt5(inactive)=X5*nx+Y5*ny+Z5*nz
Zt6(inactive)=X6*nx+Y6*ny+Z6*nz
Zt7(inactive)=X7*nx+Y7*ny+Z7*nz
```

PUCKB2(inactive)=SQRT(2/7)*(Zt1-0.222520934*(Zt2+Zt7)-0.900968867*(Zt3+Zt6)+0.623489801*(Zt4+Zt5))
PUCKA2(inactive)=-1*SQRT(2/7)*(0.974927912*(Zt2-Zt7)+0.433883739*(Zt6-Zt3)+0.781831482*(Zt5-Zt4))
PUCKB3(inactive)=SQRT(2/7)*(Zt1-0.900968867*(Zt2+Zt7)+0.623489801*(Zt3+Zt6)-0.222520934*(Zt4+Zt5))
PUCKA3(inactive)=-1*SQRT(2/7)*(0.433883739*(Zt2-Zt7)+0.781831482*(Zt6-Zt3)+0.974927912*(Zt4-Zt5))
PHI3=57.29577951*(PUCKA3/(SQRT(PUCKA3**2)+0.0001))*ARCCOS(PUCKB3/SQRT(PUCKA3*PUCKA3+PUCKB3*PUCKB3))
q2=SQRT(PUCKA2*PUCKA2+PUCKB2*PUCKB2)*0.52918
q3=SQRT(PUCKA3*PUCKA3+PUCKB3*PUCKB3)*0.52918
PHI3(NSteps=118,StepSize=3.0)

%mem=8GB
#p opt=(modredundant,newton) b972/def2svp nosymm maxdisk=8GB scf=xqc geom=GIC

SAM dihedral scan

0 1

C	-2.63662100	1.01596500	-1.29269800
C	-1.91620700	1.07735700	1.07427300
H	-1.63178000	0.93295800	-1.76315600
C	-3.23516600	-0.38958900	-1.19069100
H	-3.24911900	1.62060500	-1.98054000
C	-2.63300400	-0.18341900	1.61067800
H	-1.86731800	1.82459700	1.88028100
H	-0.86164100	0.82164900	0.83226900
H	-3.53919000	-0.70896000	-2.19929700
C	-4.42450500	-0.51556100	-0.23651600
H	-2.45277500	-1.10306200	-0.89138800
C	-4.11926200	-0.23044400	1.25625800
H	-2.13428200	-1.09362200	1.24598100
H	-2.51861800	-0.21202800	2.70463000
H	-4.81751400	-1.53915200	-0.33728900
H	-5.23456700	0.14757700	-0.57598700
H	-4.57365100	0.72645900	1.54716200
H	-4.60308000	-1.00067400	1.87583200
C	-2.62189900	1.70624600	-0.02175900
H	-2.18266954	2.66350891	-0.21050540
H	-3.63049375	1.83599032	0.31111191

XC(inactive)=XCntr(19,1,4,10,12,6,2)
YC(inactive)=YCntr(19,1,4,10,12,6,2)
ZC(inactive)=ZCntr(19,1,4,10,12,6,2)
X1(inactive)=X(19)-XC
X2(inactive)=X(1)-XC
X3(inactive)=X(4)-XC
X4(inactive)=X(10)-XC
X5(inactive)=X(12)-XC
X6(inactive)=X(6)-XC
X7(inactive)=X(2)-XC
Y1(inactive)=Y(19)-YC
Y2(inactive)=Y(1)-YC
Y3(inactive)=Y(4)-YC

Y4(inactive)=Y(10)-YC
Y5(inactive)=Y(12)-YC
Y6(inactive)=Y(6)-YC
Y7(inactive)=Y(2)-YC
Z1(inactive)=Z(19)-ZC
Z2(inactive)=Z(1)-ZC
Z3(inactive)=Z(4)-ZC
Z4(inactive)=Z(10)-ZC
Z5(inactive)=Z(12)-ZC
Z6(inactive)=Z(6)-ZC
Z7(inactive)=Z(2)-ZC
Rpx(inactive)=0.781831482*(X2-X7)+0.974927912*(X3-X6)+0.433883739*(X4-X5)
Rpy(inactive)=0.781831482*(Y2-Y7)+0.974927912*(Y3-Y6)+0.433883739*(Y4-Y5)
Rpz(inactive)=0.781831482*(Z2-Z7)+0.974927912*(Z3-Z6)+0.433883739*(Z4-Z5)
Rppx(inactive)=X1+0.623489801*(X2+X7)-0.222520934*(X3+X6)-0.900968867*(X4+X5)
Rppy(inactive)=Y1+0.623489801*(Y2+Y7)-0.222520934*(Y3+Y6)-0.900968867*(Y4+Y5)
Rppz(inactive)=Z1+0.623489801*(Z2+Z7)-0.222520934*(Z3+Z6)-0.900968867*(Z4+Z5)
prx(inactive)=Rpy*Rppz-Rppy*Rpz
pry(inactive)=Rpz*Rppx-Rppz*Rpx
prz(inactive)=Rpx*Rppy-Rppx*Rpy
nx(inactive)=prx/SQRT(prx*prx+pry*pry+prz*prz)
ny(inactive)=pry/SQRT(prx*prx+pry*pry+prz*prz)
nz(inactive)=prz/SQRT(prx*prx+pry*pry+prz*prz)
Zt1(inactive)=X1*nx+Y1*ny+Z1*nz
Zt2(inactive)=X2*nx+Y2*ny+Z2*nz
Zt3(inactive)=X3*nx+Y3*ny+Z3*nz
Zt4(inactive)=X4*nx+Y4*ny+Z4*nz
Zt5(inactive)=X5*nx+Y5*ny+Z5*nz
Zt6(inactive)=X6*nx+Y6*ny+Z6*nz
Zt7(inactive)=X7*nx+Y7*ny+Z7*nz
PUCKB2(inactive)=SQRT(2/7)*(Zt1-0.222520934*(Zt2+Zt7)-0.900968867*(Zt3+Zt6)+0.623489801*(Zt4+Zt5))
PUCKA2(inactive)=-1*SQRT(2/7)*(0.974927912*(Zt2-Zt7)+0.433883739*(Zt6-Zt3)+0.781831482*(Zt5-Zt4))
PUCKB3(inactive)=SQRT(2/7)*(Zt1-0.900968867*(Zt2+Zt7)+0.623489801*(Zt3+Zt6)-0.222520934*(Zt4+Zt5))
PUCKA3(inactive)=-1*SQRT(2/7)*(0.433883739*(Zt2-Zt7)+0.781831482*(Zt6-Zt3)+0.974927912*(Zt4-Zt5))
PHI3=57.29577951*(PUCKA3/(SQRT(PUCKA3**2)+0.0001))*ARCCOS(PUCKB3/SQRT(PUCKA3*PUCKA3+PUCKB3*PUCKB3))
q2=SQRT(PUCKA2*PUCKA2+PUCKB2*PUCKB2)*0.52918
q3=SQRT(PUCKA3*PUCKA3+PUCKB3*PUCKB3)*0.52918
PHI3(NSteps=118,StepSize=3.0)

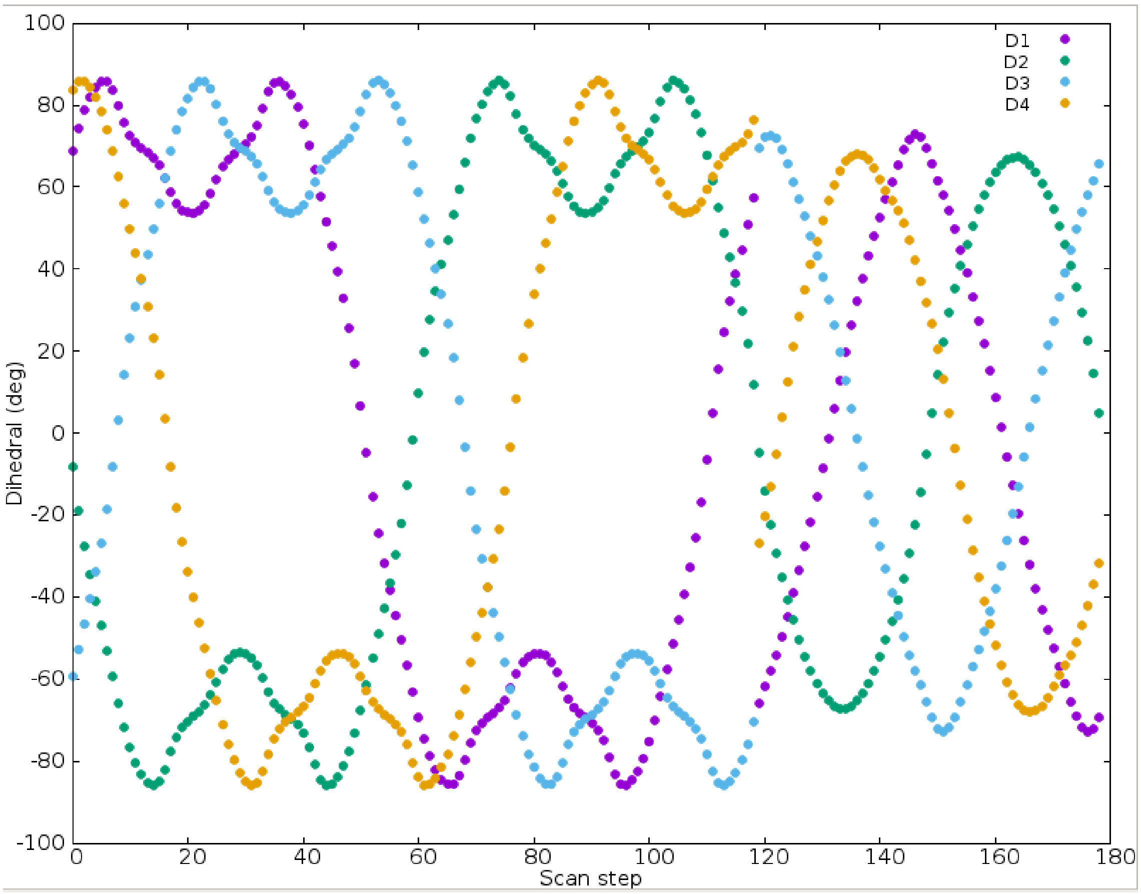

Figure S15. Evolution of the D1, D2 and D3 endocyclic dihedral angles along the scan conformational coordinate of the X=CH_2 7-membered ring ($C_6H_{10}X$) molecule.

$C_6H_{12}O$ – TC/C plane and TB/B plane

%mem=8GB
#p opt=(modredundant,newton) b972/def2svp nosymm maxdisk=8GB scf=xqc geom=GIC

SAM dihedral scan

0 1

O	-1.845876	1.528675	0.018960
C	-2.208676	0.845024	-1.149494
C	-1.868696	0.789078	1.211181
H	-1.556776	-0.041043	-1.297788
C	-3.673092	0.417252	-1.231869
H	-1.990355	1.543141	-1.970158
C	-3.272673	0.454704	1.716388
H	-1.357312	1.421119	1.951028
H	-1.268308	-0.138223	1.108761
H	-4.301110	1.276053	-0.946723
C	-4.051910	-0.835145	-0.441131

H	-3.896643	0.222541	-2.293120
C	-3.929261	-0.783489	1.100880
H	-3.215874	0.298838	2.805510
H	-3.898187	1.349097	1.573451
H	-3.431601	-1.662359	-0.822000
H	-5.083148	-1.109120	-0.708689
H	-4.929649	-0.885471	1.547894
H	-3.372967	-1.670236	1.442436

```
XC(inactive)=XCntr(1,2,5,11,13,7,3)
YC(inactive)=YCntr(1,2,5,11,13,7,3)
ZC(inactive)=ZCntr(1,2,5,11,13,7,3)
X1(inactive)=X(1)-XC
X2(inactive)=X(2)-XC
X3(inactive)=X(5)-XC
X4(inactive)=X(11)-XC
X5(inactive)=X(13)-XC
X6(inactive)=X(7)-XC
X7(inactive)=X(3)-XC
Y1(inactive)=Y(1)-YC
Y2(inactive)=Y(2)-YC
Y3(inactive)=Y(5)-YC
Y4(inactive)=Y(11)-YC
Y5(inactive)=Y(13)-YC
Y6(inactive)=Y(7)-YC
Y7(inactive)=Y(3)-YC
Z1(inactive)=Z(1)-ZC
Z2(inactive)=Z(2)-ZC
Z3(inactive)=Z(5)-ZC
Z4(inactive)=Z(11)-ZC
Z5(inactive)=Z(13)-ZC
Z6(inactive)=Z(7)-ZC
Z7(inactive)=Z(3)-ZC
Rpx(inactive)=0.781831482*(X2-X7)+0.974927912*(X3-X6)+0.433883739*(X4-X5)
Rpy(inactive)=0.781831482*(Y2-Y7)+0.974927912*(Y3-Y6)+0.433883739*(Y4-Y5)
Rpz(inactive)=0.781831482*(Z2-Z7)+0.974927912*(Z3-Z6)+0.433883739*(Z4-Z5)
Rppx(inactive)=X1+0.623489801*(X2+X7)-0.222520934*(X3+X6)-0.900968867*(X4+X5)
Rppy(inactive)=Y1+0.623489801*(Y2+Y7)-0.222520934*(Y3+Y6)-0.900968867*(Y4+Y5)
Rppz(inactive)=Z1+0.623489801*(Z2+Z7)-0.222520934*(Z3+Z6)-0.900968867*(Z4+Z5)
prx(inactive)=Rpy*Rppz-Rppy*Rpz
pry(inactive)=Rpz*Rppx-Rppz*Rpx
prz(inactive)=Rpx*Rppy-Rppx*Rpy
nx(inactive)=prx/SQRT(prx*prx+pry*pry+prz*prz)
ny(inactive)=pry/SQRT(prx*prx+pry*pry+prz*prz)
nz(inactive)=prz/SQRT(prx*prx+pry*pry+prz*prz)
Zt1(inactive)=X1*nx+Y1*ny+Z1*nz
Zt2(inactive)=X2*nx+Y2*ny+Z2*nz
Zt3(inactive)=X3*nx+Y3*ny+Z3*nz
Zt4(inactive)=X4*nx+Y4*ny+Z4*nz
Zt5(inactive)=X5*nx+Y5*ny+Z5*nz
Zt6(inactive)=X6*nx+Y6*ny+Z6*nz
Zt7(inactive)=X7*nx+Y7*ny+Z7*nz
PUCKB2(inactive)=SQRT(2/7)*(Zt1-0.222520934*(Zt2+Zt7)-
0.900968867*(Zt3+Zt6)+0.623489801*(Zt4+Zt5))
```

PUCKA2(inactive)=-1*SQRT(2/7)*(0.974927912*(Zt2-Zt7)+0.433883739*(Zt6-Zt3)+0.781831482*(Zt5-Zt4))
PUCKB3(inactive)=SQRT(2/7)*(Zt1-0.900968867*(Zt2+Zt7)+0.623489801*(Zt3+Zt6)-0.222520934*(Zt4+Zt5))
PUCKA3(inactive)=-1*SQRT(2/7)*(0.433883739*(Zt2-Zt7)+0.781831482*(Zt6-Zt3)+0.974927912*(Zt4-Zt5))
PHI3=57.29577951*(PUCKA3/(SQRT(PUCKA3**2)+0.0001))*ARCCOS(PUCKB3/SQRT(PUCKA3*PUCKA3+PUCKB3*PUCKB3))
q2=SQRT(PUCKA2*PUCKA2+PUCKB2*PUCKB2)*0.52918
q3=SQRT(PUCKA3*PUCKA3+PUCKB3*PUCKB3)*0.52918
PHI3(NSteps=118,StepSize=3.0)

%mem=8GB
#p opt=(modredundant,newton) b972/def2svp nosymm maxdisk=8GB scf=xqc geom=GIC

SAM dihedral scan

0 1

O	-2.65133600	1.68992500	-0.04737900
C	-2.59780800	0.96738600	-1.24935900
C	-1.98233500	1.13293400	1.05387100
H	-1.55343600	0.87609900	-1.60694600
C	-3.25705700	-0.41701200	-1.18515700
H	-3.13182800	1.59635600	-1.97620300
C	-2.60402300	-0.17390000	1.57779500
H	-2.04213300	1.90574500	1.83345100
H	-0.90816700	0.98563700	0.82899100
H	-3.58485000	-0.68668200	-2.20100700
C	-4.45110400	-0.46604500	-0.22938800
H	-2.52161400	-1.18479700	-0.90446500
C	-4.09789000	-0.28603200	1.27217900
H	-2.07313900	-1.04871400	1.17500600
H	-2.44841200	-0.21603200	2.66663700
H	-4.95941700	-1.43136200	-0.37085700
H	-5.18008200	0.30077900	-0.53044400
H	-4.60371500	0.60869500	1.66348100
H	-4.50376500	-1.13254500	1.84530800

XC(inactive)=XCntr(1,2,5,11,13,7,3)
YC(inactive)=YCntr(1,2,5,11,13,7,3)
ZC(inactive)=ZCntr(1,2,5,11,13,7,3)
X1(inactive)=X(1)-XC
X2(inactive)=X(2)-XC
X3(inactive)=X(5)-XC
X4(inactive)=X(11)-XC
X5(inactive)=X(13)-XC
X6(inactive)=X(7)-XC
X7(inactive)=X(3)-XC
Y1(inactive)=Y(1)-YC
Y2(inactive)=Y(2)-YC
Y3(inactive)=Y(5)-YC
Y4(inactive)=Y(11)-YC
Y5(inactive)=Y(13)-YC
Y6(inactive)=Y(7)-YC
Y7(inactive)=Y(3)-YC

```
Z1(inactive)=Z(1)-ZC
Z2(inactive)=Z(2)-ZC
Z3(inactive)=Z(5)-ZC
Z4(inactive)=Z(11)-ZC
Z5(inactive)=Z(13)-ZC
Z6(inactive)=Z(7)-ZC
Z7(inactive)=Z(3)-ZC
Rpx(inactive)=0.781831482*(X2-X7)+0.974927912*(X3-X6)+0.433883739*(X4-X5)
Rpy(inactive)=0.781831482*(Y2-Y7)+0.974927912*(Y3-Y6)+0.433883739*(Y4-Y5)
Rpz(inactive)=0.781831482*(Z2-Z7)+0.974927912*(Z3-Z6)+0.433883739*(Z4-Z5)
Rppx(inactive)=X1+0.623489801*(X2+X7)-0.222520934*(X3+X6)-0.900968867*(X4+X5)
Rppy(inactive)=Y1+0.623489801*(Y2+Y7)-0.222520934*(Y3+Y6)-0.900968867*(Y4+Y5)
Rppz(inactive)=Z1+0.623489801*(Z2+Z7)-0.222520934*(Z3+Z6)-0.900968867*(Z4+Z5)
prx(inactive)=Rpy*Rppz-Rppy*Rpz
pry(inactive)=Rpz*Rppx-Rppz*Rpx
prz(inactive)=Rpx*Rppy-Rppx*Rpy
nx(inactive)=prx/SQRT(prx*prx+pry*pry+prz*prz)
ny(inactive)=pry/SQRT(prx*prx+pry*pry+prz*prz)
nz(inactive)=prz/SQRT(prx*prx+pry*pry+prz*prz)
Zt1(inactive)=X1*nx+Y1*ny+Z1*nz
Zt2(inactive)=X2*nx+Y2*ny+Z2*nz
Zt3(inactive)=X3*nx+Y3*ny+Z3*nz
Zt4(inactive)=X4*nx+Y4*ny+Z4*nz
Zt5(inactive)=X5*nx+Y5*ny+Z5*nz
Zt6(inactive)=X6*nx+Y6*ny+Z6*nz
Zt7(inactive)=X7*nx+Y7*ny+Z7*nz
PUCKB2(inactive)=SQRT(2/7)*(Zt1-0.222520934*(Zt2+Zt7)-
0.900968867*(Zt3+Zt6)+0.623489801*(Zt4+Zt5))
PUCKA2(inactive)=-1*SQRT(2/7)*(0.974927912*(Zt2-Zt7)+0.433883739*(Zt6-Zt3)+0.781831482*(Zt5-
Zt4))
PUCKB3(inactive)=SQRT(2/7)*(Zt1-0.900968867*(Zt2+Zt7)+0.623489801*(Zt3+Zt6)-
0.222520934*(Zt4+Zt5))
PUCKA3(inactive)=-1*SQRT(2/7)*(0.433883739*(Zt2-Zt7)+0.781831482*(Zt6-Zt3)+0.974927912*(Zt4-
Zt5))
PHI2=57.29577951*(PUCKA2/(SQRT(PUCKA2**2)+0.0001))*ARCCOS(PUCKB2/SQRT(PUCKA2*PUC
KA2+PUCKB2*PUCKB2))
q2=SQRT(PUCKA2*PUCKA2+PUCKB2*PUCKB2)*0.52918
q3=SQRT(PUCKA3*PUCKA3+PUCKB3*PUCKB3)*0.52918
PHI2(NSteps=59,StepSize=-6.0)
```

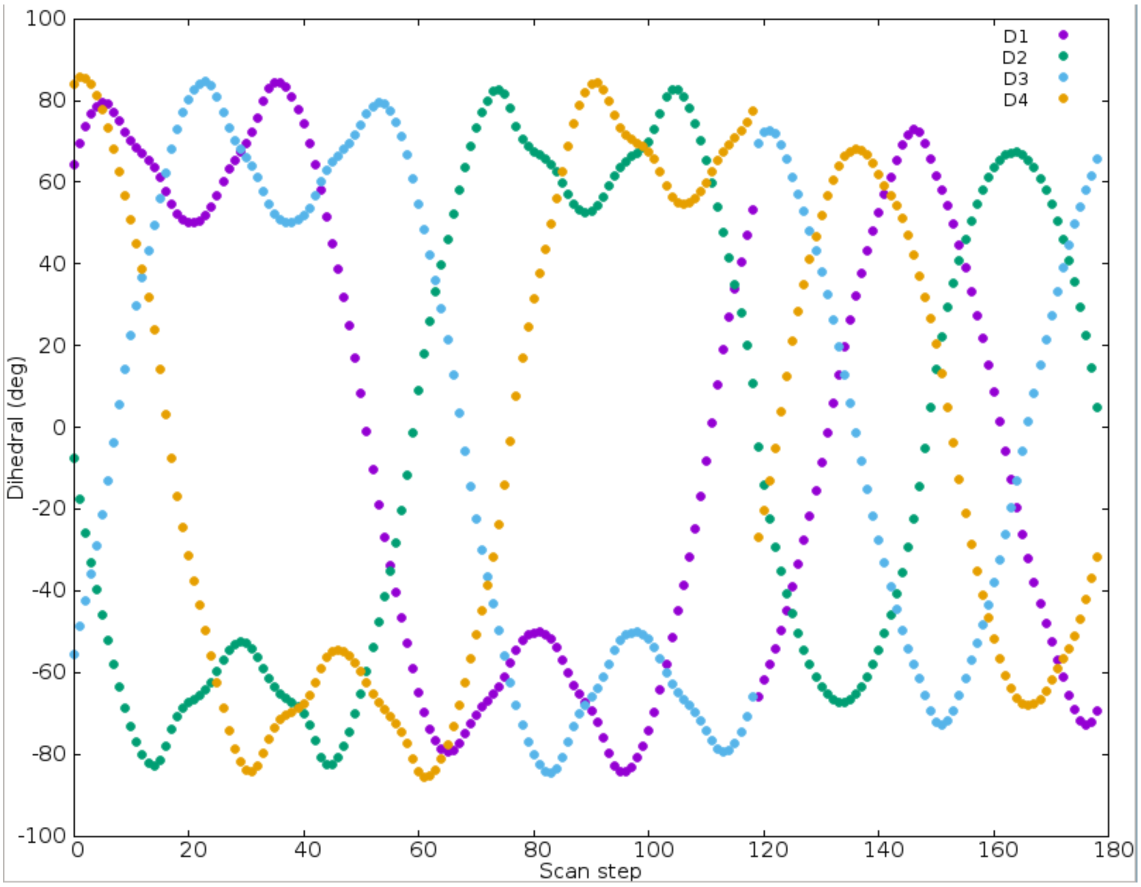

Figure S16. Evolution of the D1, D2 and D3 endocyclic dihedral angles along the scan conformational coordinate of the X=O 7-membered ring ($C_6H_{10}X$) molecule.

$C_6H_{12}S$ – TC/C plane and TB/B plane

%mem=8GB
#p opt=(modredundant,newton) b972/def2svp nosymm maxdisk=8GB scf=xqc geom=GIC

SAM dihedral scan

0 1

S	-1.845876	1.528675	0.018960
C	-2.208676	0.845024	-1.149494
C	-1.868696	0.789078	1.211181
H	-1.556776	-0.041043	-1.297788
C	-3.673092	0.417252	-1.231869
H	-1.990355	1.543141	-1.970158
C	-3.272673	0.454704	1.716388
H	-1.357312	1.421119	1.951028
H	-1.268308	-0.138223	1.108761
H	-4.301110	1.276053	-0.946723
C	-4.051910	-0.835145	-0.441131
H	-3.896643	0.222541	-2.293120
C	-3.929261	-0.783489	1.100880

H	-3.215874	0.298838	2.805510
H	-3.898187	1.349097	1.573451
H	-3.431601	-1.662359	-0.822000
H	-5.083148	-1.109120	-0.708689
H	-4.929649	-0.885471	1.547894
H	-3.372967	-1.670236	1.442436

```
XC(inactive)=XCntr(1,2,5,11,13,7,3)
YC(inactive)=YCntr(1,2,5,11,13,7,3)
ZC(inactive)=ZCntr(1,2,5,11,13,7,3)
X1(inactive)=X(1)-XC
X2(inactive)=X(2)-XC
X3(inactive)=X(5)-XC
X4(inactive)=X(11)-XC
X5(inactive)=X(13)-XC
X6(inactive)=X(7)-XC
X7(inactive)=X(3)-XC
Y1(inactive)=Y(1)-YC
Y2(inactive)=Y(2)-YC
Y3(inactive)=Y(5)-YC
Y4(inactive)=Y(11)-YC
Y5(inactive)=Y(13)-YC
Y6(inactive)=Y(7)-YC
Y7(inactive)=Y(3)-YC
Z1(inactive)=Z(1)-ZC
Z2(inactive)=Z(2)-ZC
Z3(inactive)=Z(5)-ZC
Z4(inactive)=Z(11)-ZC
Z5(inactive)=Z(13)-ZC
Z6(inactive)=Z(7)-ZC
Z7(inactive)=Z(3)-ZC
Rpx(inactive)=0.781831482*(X2-X7)+0.974927912*(X3-X6)+0.433883739*(X4-X5)
Rpy(inactive)=0.781831482*(Y2-Y7)+0.974927912*(Y3-Y6)+0.433883739*(Y4-Y5)
Rpz(inactive)=0.781831482*(Z2-Z7)+0.974927912*(Z3-Z6)+0.433883739*(Z4-Z5)
Rppx(inactive)=X1+0.623489801*(X2+X7)-0.222520934*(X3+X6)-0.900968867*(X4+X5)
Rppy(inactive)=Y1+0.623489801*(Y2+Y7)-0.222520934*(Y3+Y6)-0.900968867*(Y4+Y5)
Rppz(inactive)=Z1+0.623489801*(Z2+Z7)-0.222520934*(Z3+Z6)-0.900968867*(Z4+Z5)
prx(inactive)=Rpy*Rppz-Rppy*Rpz
pry(inactive)=Rpz*Rppx-Rppz*Rpx
prz(inactive)=Rpx*Rppy-Rppx*Rpy
nx(inactive)=prx/SQRT(prx*prx+pry*pry+prz*prz)
ny(inactive)=pry/SQRT(prx*prx+pry*pry+prz*prz)
nz(inactive)=prz/SQRT(prx*prx+pry*pry+prz*prz)
Zt1(inactive)=X1*nx+Y1*ny+Z1*nz
Zt2(inactive)=X2*nx+Y2*ny+Z2*nz
Zt3(inactive)=X3*nx+Y3*ny+Z3*nz
Zt4(inactive)=X4*nx+Y4*ny+Z4*nz
Zt5(inactive)=X5*nx+Y5*ny+Z5*nz
Zt6(inactive)=X6*nx+Y6*ny+Z6*nz
Zt7(inactive)=X7*nx+Y7*ny+Z7*nz
PUCKB2(inactive)=SQRT(2/7)*(Zt1-0.222520934*(Zt2+Zt7)-
0.900968867*(Zt3+Zt6)+0.623489801*(Zt4+Zt5))
PUCKA2(inactive)=-1*SQRT(2/7)*(0.974927912*(Zt2-Zt7)+0.433883739*(Zt6-Zt3)+0.781831482*(Zt5-
Zt4))
```

PUCKB3(inactive)=SQRT(2/7)*(Zt1-0.900968867*(Zt2+Zt7)+0.623489801*(Zt3+Zt6)-0.222520934*(Zt4+Zt5))
PUCKA3(inactive)=-1*SQRT(2/7)*(0.433883739*(Zt2-Zt7)+0.781831482*(Zt6-Zt3)+0.974927912*(Zt4-Zt5))
PHI3=57.29577951*(PUCKA3/(SQRT(PUCKA3**2)+0.0001))*ARCCOS(PUCKB3/SQRT(PUCKA3*PUCKA3+PUCKB3*PUCKB3))
q2=SQRT(PUCKA2*PUCKA2+PUCKB2*PUCKB2)*0.52918
q3=SQRT(PUCKA3*PUCKA3+PUCKB3*PUCKB3)*0.52918
PHI3(NSteps=118,StepSize=3.0)

%mem=8GB
#p opt=(modredundant,newton) b972/def2svp nosymm maxdisk=8GB scf=xqc geom=GIC

SAM dihedral scan

0 1

S	-2.65133600	1.68992500	-0.04737900
C	-2.59780800	0.96738600	-1.24935900
C	-1.98233500	1.13293400	1.05387100
H	-1.55343600	0.87609900	-1.60694600
C	-3.25705700	-0.41701200	-1.18515700
H	-3.13182800	1.59635600	-1.97620300
C	-2.60402300	-0.17390000	1.57779500
H	-2.04213300	1.90574500	1.83345100
H	-0.90816700	0.98563700	0.82899100
H	-3.58485000	-0.68668200	-2.20100700
C	-4.45110400	-0.46604500	-0.22938800
H	-2.52161400	-1.18479700	-0.90446500
C	-4.09789000	-0.28603200	1.27217900
H	-2.07313900	-1.04871400	1.17500600
H	-2.44841200	-0.21603200	2.66663700
H	-4.95941700	-1.43136200	-0.37085700
H	-5.18008200	0.30077900	-0.53044400
H	-4.60371500	0.60869500	1.66348100
H	-4.50376500	-1.13254500	1.84530800

XC(inactive)=XCntr(1,2,5,11,13,7,3)
YC(inactive)=YCntr(1,2,5,11,13,7,3)
ZC(inactive)=ZCntr(1,2,5,11,13,7,3)
X1(inactive)=X(1)-XC
X2(inactive)=X(2)-XC
X3(inactive)=X(5)-XC
X4(inactive)=X(11)-XC
X5(inactive)=X(13)-XC
X6(inactive)=X(7)-XC
X7(inactive)=X(3)-XC
Y1(inactive)=Y(1)-YC
Y2(inactive)=Y(2)-YC
Y3(inactive)=Y(5)-YC
Y4(inactive)=Y(11)-YC
Y5(inactive)=Y(13)-YC
Y6(inactive)=Y(7)-YC
Y7(inactive)=Y(3)-YC
Z1(inactive)=Z(1)-ZC
Z2(inactive)=Z(2)-ZC

Z3(inactive)=Z(5)-ZC
Z4(inactive)=Z(11)-ZC
Z5(inactive)=Z(13)-ZC
Z6(inactive)=Z(7)-ZC
Z7(inactive)=Z(3)-ZC
Rpx(inactive)=0.781831482*(X2-X7)+0.974927912*(X3-X6)+0.433883739*(X4-X5)
Rpy(inactive)=0.781831482*(Y2-Y7)+0.974927912*(Y3-Y6)+0.433883739*(Y4-Y5)
Rpz(inactive)=0.781831482*(Z2-Z7)+0.974927912*(Z3-Z6)+0.433883739*(Z4-Z5)
Rppx(inactive)=X1+0.623489801*(X2+X7)-0.222520934*(X3+X6)-0.900968867*(X4+X5)
Rppy(inactive)=Y1+0.623489801*(Y2+Y7)-0.222520934*(Y3+Y6)-0.900968867*(Y4+Y5)
Rppz(inactive)=Z1+0.623489801*(Z2+Z7)-0.222520934*(Z3+Z6)-0.900968867*(Z4+Z5)
prx(inactive)=Rpy*Rppz-Rppy*Rpz
pry(inactive)=Rpz*Rppx-Rppz*Rpx
prz(inactive)=Rpx*Rppy-Rppx*Rpy
nx(inactive)=prx/SQRT(prx*prx+pry*pry+prz*prz)
ny(inactive)=pry/SQRT(prx*prx+pry*pry+prz*prz)
nz(inactive)=prz/SQRT(prx*prx+pry*pry+prz*prz)
Zt1(inactive)=X1*nx+Y1*ny+Z1*nz
Zt2(inactive)=X2*nx+Y2*ny+Z2*nz
Zt3(inactive)=X3*nx+Y3*ny+Z3*nz
Zt4(inactive)=X4*nx+Y4*ny+Z4*nz
Zt5(inactive)=X5*nx+Y5*ny+Z5*nz
Zt6(inactive)=X6*nx+Y6*ny+Z6*nz
Zt7(inactive)=X7*nx+Y7*ny+Z7*nz
PUCKB2(inactive)=SQRT(2/7)*(Zt1-0.222520934*(Zt2+Zt7)-0.900968867*(Zt3+Zt6)+0.623489801*(Zt4+Zt5))
PUCKA2(inactive)=-1*SQRT(2/7)*(0.974927912*(Zt2-Zt7)+0.433883739*(Zt6-Zt3)+0.781831482*(Zt5-Zt4))
PUCKB3(inactive)=SQRT(2/7)*(Zt1-0.900968867*(Zt2+Zt7)+0.623489801*(Zt3+Zt6)-0.222520934*(Zt4+Zt5))
PUCKA3(inactive)=-1*SQRT(2/7)*(0.433883739*(Zt2-Zt7)+0.781831482*(Zt6-Zt3)+0.974927912*(Zt4-Zt5))
PHI2=57.29577951*(PUCKA2/(SQRT(PUCKA2**2)+0.0001))*ARCCOS(PUCKB2/SQRT(PUCKA2*PUCKA2+PUCKB2*PUCKB2))
q2=SQRT(PUCKA2*PUCKA2+PUCKB2*PUCKB2)*0.52918
q3=SQRT(PUCKA3*PUCKA3+PUCKB3*PUCKB3)*0.52918
PHI2(NSteps=59,StepSize=-6.0)

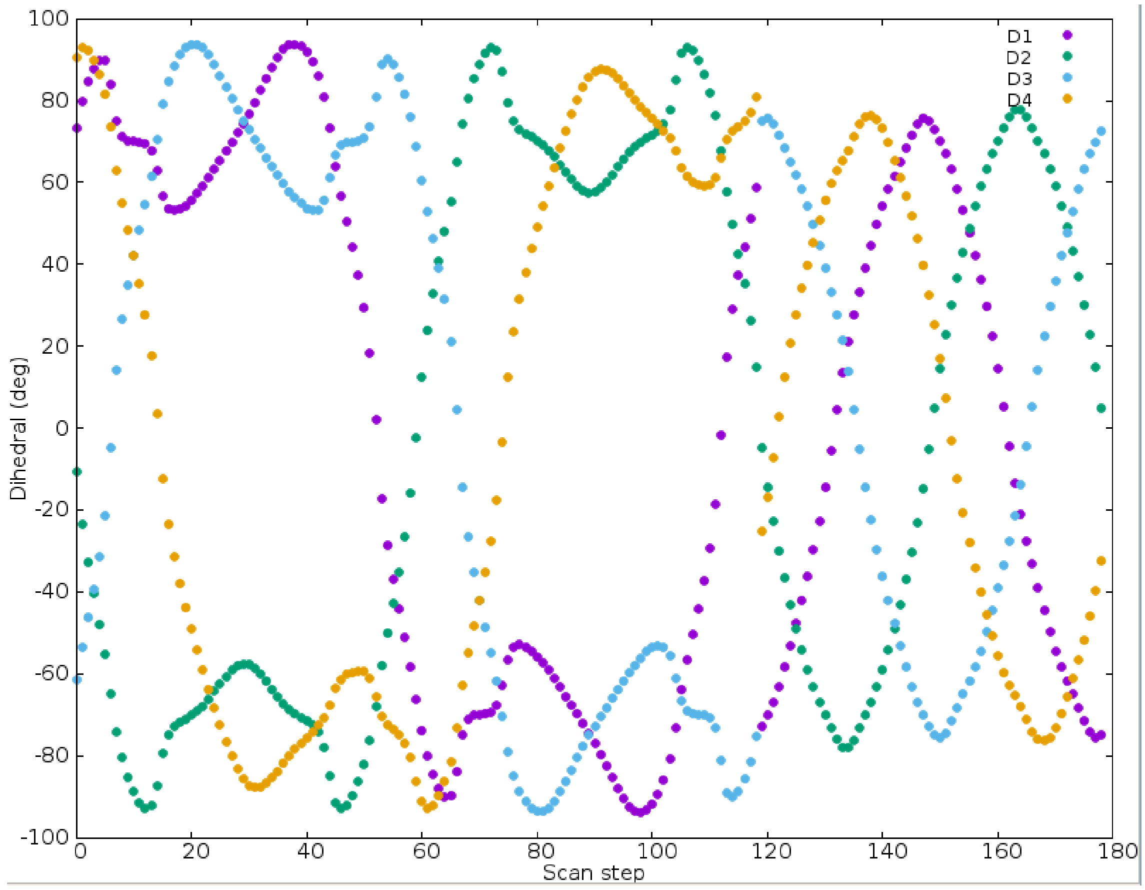

Figure S17. Evolution of the D1, D2 and D3 endocyclic dihedral angles along the scan conformational coordinate of the X=S 7-membered ring ($C_6H_{10}X$) molecule.

$C_6H_{12}SO_2$ – TC/C plane and TB/B plane

%mem=8GB
#p opt=(modredundant,newton) b972/def2svp nosymm maxdisk=8GB scf=xqc geom=GIC

SAM dihedral scan

0 1

S	-1.845876	1.528675	0.018960
C	-2.208676	0.845024	-1.149494
C	-1.868696	0.789078	1.211181
H	-1.556776	-0.041043	-1.297788
C	-3.673092	0.417252	-1.231869
H	-1.990355	1.543141	-1.970158
C	-3.272673	0.454704	1.716388
H	-1.357312	1.421119	1.951028
H	-1.268308	-0.138223	1.108761
H	-4.301110	1.276053	-0.946723
C	-4.051910	-0.835145	-0.441131
H	-3.896643	0.222541	-2.293120
C	-3.929261	-0.783489	1.100880

H	-3.215874	0.298838	2.805510
H	-3.898187	1.349097	1.573451
H	-3.431601	-1.662359	-0.822000
H	-5.083148	-1.109120	-0.708689
H	-4.929649	-0.885471	1.547894
H	-3.372967	-1.670236	1.442436
O	-4.249771	1.869124	0.379434
O	-1.978155	3.191658	-0.347016

```
XC(inactive)=XCntr(1,2,5,11,13,7,3)
YC(inactive)=YCntr(1,2,5,11,13,7,3)
ZC(inactive)=ZCntr(1,2,5,11,13,7,3)
X1(inactive)=X(1)-XC
X2(inactive)=X(2)-XC
X3(inactive)=X(5)-XC
X4(inactive)=X(11)-XC
X5(inactive)=X(13)-XC
X6(inactive)=X(7)-XC
X7(inactive)=X(3)-XC
Y1(inactive)=Y(1)-YC
Y2(inactive)=Y(2)-YC
Y3(inactive)=Y(5)-YC
Y4(inactive)=Y(11)-YC
Y5(inactive)=Y(13)-YC
Y6(inactive)=Y(7)-YC
Y7(inactive)=Y(3)-YC
Z1(inactive)=Z(1)-ZC
Z2(inactive)=Z(2)-ZC
Z3(inactive)=Z(5)-ZC
Z4(inactive)=Z(11)-ZC
Z5(inactive)=Z(13)-ZC
Z6(inactive)=Z(7)-ZC
Z7(inactive)=Z(3)-ZC
Rpx(inactive)=0.781831482*(X2-X7)+0.974927912*(X3-X6)+0.433883739*(X4-X5)
Rpy(inactive)=0.781831482*(Y2-Y7)+0.974927912*(Y3-Y6)+0.433883739*(Y4-Y5)
Rpz(inactive)=0.781831482*(Z2-Z7)+0.974927912*(Z3-Z6)+0.433883739*(Z4-Z5)
Rppx(inactive)=X1+0.623489801*(X2+X7)-0.222520934*(X3+X6)-0.900968867*(X4+X5)
Rppy(inactive)=Y1+0.623489801*(Y2+Y7)-0.222520934*(Y3+Y6)-0.900968867*(Y4+Y5)
Rppz(inactive)=Z1+0.623489801*(Z2+Z7)-0.222520934*(Z3+Z6)-0.900968867*(Z4+Z5)
prx(inactive)=Rpy*Rppz-Rppy*Rpz
pry(inactive)=Rpz*Rppx-Rppz*Rpx
prz(inactive)=Rpx*Rppy-Rppx*Rpy
nx(inactive)=prx/SQRT(prx*prx+pry*pry+prz*prz)
ny(inactive)=pry/SQRT(prx*prx+pry*pry+prz*prz)
nz(inactive)=prz/SQRT(prx*prx+pry*pry+prz*prz)
Zt1(inactive)=X1*nx+Y1*ny+Z1*nz
Zt2(inactive)=X2*nx+Y2*ny+Z2*nz
Zt3(inactive)=X3*nx+Y3*ny+Z3*nz
Zt4(inactive)=X4*nx+Y4*ny+Z4*nz
Zt5(inactive)=X5*nx+Y5*ny+Z5*nz
Zt6(inactive)=X6*nx+Y6*ny+Z6*nz
Zt7(inactive)=X7*nx+Y7*ny+Z7*nz
PUCKB2(inactive)=SQRT(2/7)*(Zt1-0.222520934*(Zt2+Zt7)-
0.900968867*(Zt3+Zt6)+0.623489801*(Zt4+Zt5))
```

PUCKA2(inactive)=-1*SQRT(2/7)*(0.974927912*(Zt2-Zt7)+0.433883739*(Zt6-Zt3)+0.781831482*(Zt5-Zt4))
PUCKB3(inactive)=SQRT(2/7)*(Zt1-0.900968867*(Zt2+Zt7)+0.623489801*(Zt3+Zt6)-0.222520934*(Zt4+Zt5))
PUCKA3(inactive)=-1*SQRT(2/7)*(0.433883739*(Zt2-Zt7)+0.781831482*(Zt6-Zt3)+0.974927912*(Zt4-Zt5))
PHI3=57.29577951*(PUCKA3/(SQRT(PUCKA3**2)+0.0001))*ARCCOS(PUCKB3/SQRT(PUCKA3*PUCKA3+PUCKB3*PUCKB3))
q2=SQRT(PUCKA2*PUCKA2+PUCKB2*PUCKB2)*0.52918
q3=SQRT(PUCKA3*PUCKA3+PUCKB3*PUCKB3)*0.52918
PHI3(NSteps=118,StepSize=3.0)

%mem=8GB
#p opt=(modredundant,newton) b972/def2svp nosymm maxdisk=8GB scf=xqc geom=GIC

SAM dihedral scan

0 1

C	-2.64422300	0.94769700	-1.47559200
C	-1.91273300	1.13709600	1.26847100
H	-1.62355000	0.83853300	-1.87203600
C	-3.27240400	-0.42775100	-1.22014200
H	-3.21845100	1.49457700	-2.23825600
C	-2.60325300	-0.19235300	1.62160900
H	-1.97561100	1.83538200	2.11603400
H	-0.84072200	0.98167800	1.07616500
H	-3.61743900	-0.81982900	-2.19004200
C	-4.43965300	-0.46254500	-0.23102900
H	-2.49134400	-1.12948600	-0.89408400
C	-4.09003300	-0.27774400	1.27203500
H	-2.06121500	-1.02446400	1.14994000
H	-2.48385000	-0.35300600	2.70439000
H	-4.94203700	-1.43321500	-0.35817100
H	-5.17933300	0.29528100	-0.52876700
H	-4.58953200	0.62347100	1.65560500
H	-4.51693600	-1.11954500	1.83727200
S	-2.64979100	2.10665900	-0.07788500
O	-1.73235549	3.45669891	-0.43087840
O	-4.20229621	2.57686404	0.31902154

XC(inactive)=XCntr(19,1,4,10,12,6,2)
YC(inactive)=YCntr(19,1,4,10,12,6,2)
ZC(inactive)=ZCntr(19,1,4,10,12,6,2)
X1(inactive)=X(19)-XC
X2(inactive)=X(1)-XC
X3(inactive)=X(4)-XC
X4(inactive)=X(10)-XC
X5(inactive)=X(12)-XC
X6(inactive)=X(6)-XC
X7(inactive)=X(2)-XC
Y1(inactive)=Y(19)-YC
Y2(inactive)=Y(1)-YC
Y3(inactive)=Y(4)-YC
Y4(inactive)=Y(10)-YC
Y5(inactive)=Y(12)-YC

Y6(inactive)=Y(6)-YC
Y7(inactive)=Y(2)-YC
Z1(inactive)=Z(19)-ZC
Z2(inactive)=Z(1)-ZC
Z3(inactive)=Z(4)-ZC
Z4(inactive)=Z(10)-ZC
Z5(inactive)=Z(12)-ZC
Z6(inactive)=Z(6)-ZC
Z7(inactive)=Z(2)-ZC
Rpx(inactive)=0.781831482*(X2-X7)+0.974927912*(X3-X6)+0.433883739*(X4-X5)
Rpy(inactive)=0.781831482*(Y2-Y7)+0.974927912*(Y3-Y6)+0.433883739*(Y4-Y5)
Rpz(inactive)=0.781831482*(Z2-Z7)+0.974927912*(Z3-Z6)+0.433883739*(Z4-Z5)
Rppx(inactive)=X1+0.623489801*(X2+X7)-0.222520934*(X3+X6)-0.900968867*(X4+X5)
Rppy(inactive)=Y1+0.623489801*(Y2+Y7)-0.222520934*(Y3+Y6)-0.900968867*(Y4+Y5)
Rppz(inactive)=Z1+0.623489801*(Z2+Z7)-0.222520934*(Z3+Z6)-0.900968867*(Z4+Z5)
prx(inactive)=Rpy*Rppz-Rppy*Rpz
pry(inactive)=Rpz*Rppx-Rppz*Rpx
prz(inactive)=Rpx*Rppy-Rppx*Rpy
nx(inactive)=prx/SQRT(prx*prx+pry*pry+prz*prz)
ny(inactive)=pry/SQRT(prx*prx+pry*pry+prz*prz)
nz(inactive)=prz/SQRT(prx*prx+pry*pry+prz*prz)
Zt1(inactive)=X1*nx+Y1*ny+Z1*nz
Zt2(inactive)=X2*nx+Y2*ny+Z2*nz
Zt3(inactive)=X3*nx+Y3*ny+Z3*nz
Zt4(inactive)=X4*nx+Y4*ny+Z4*nz
Zt5(inactive)=X5*nx+Y5*ny+Z5*nz
Zt6(inactive)=X6*nx+Y6*ny+Z6*nz
Zt7(inactive)=X7*nx+Y7*ny+Z7*nz
PUCKB2(inactive)=SQRT(2/7)*(Zt1-0.222520934*(Zt2+Zt7)-0.900968867*(Zt3+Zt6)+0.623489801*(Zt4+Zt5))
PUCKA2(inactive)=-1*SQRT(2/7)*(0.974927912*(Zt2-Zt7)+0.433883739*(Zt6-Zt3)+0.781831482*(Zt5-Zt4))
PUCKB3(inactive)=SQRT(2/7)*(Zt1-0.900968867*(Zt2+Zt7)+0.623489801*(Zt3+Zt6)-0.222520934*(Zt4+Zt5))
PUCKA3(inactive)=-1*SQRT(2/7)*(0.433883739*(Zt2-Zt7)+0.781831482*(Zt6-Zt3)+0.974927912*(Zt4-Zt5))
PHI3=57.29577951*(PUCKA3/(SQRT(PUCKA3**2)+0.0001))*ARCCOS(PUCKB3/SQRT(PUCKA3*PUCKA3+PUCKB3*PUCKB3))
q2=SQRT(PUCKA2*PUCKA2+PUCKB2*PUCKB2)*0.52918
q3=SQRT(PUCKA3*PUCKA3+PUCKB3*PUCKB3)*0.52918
PHI3(NSteps=118,StepSize=3.0)

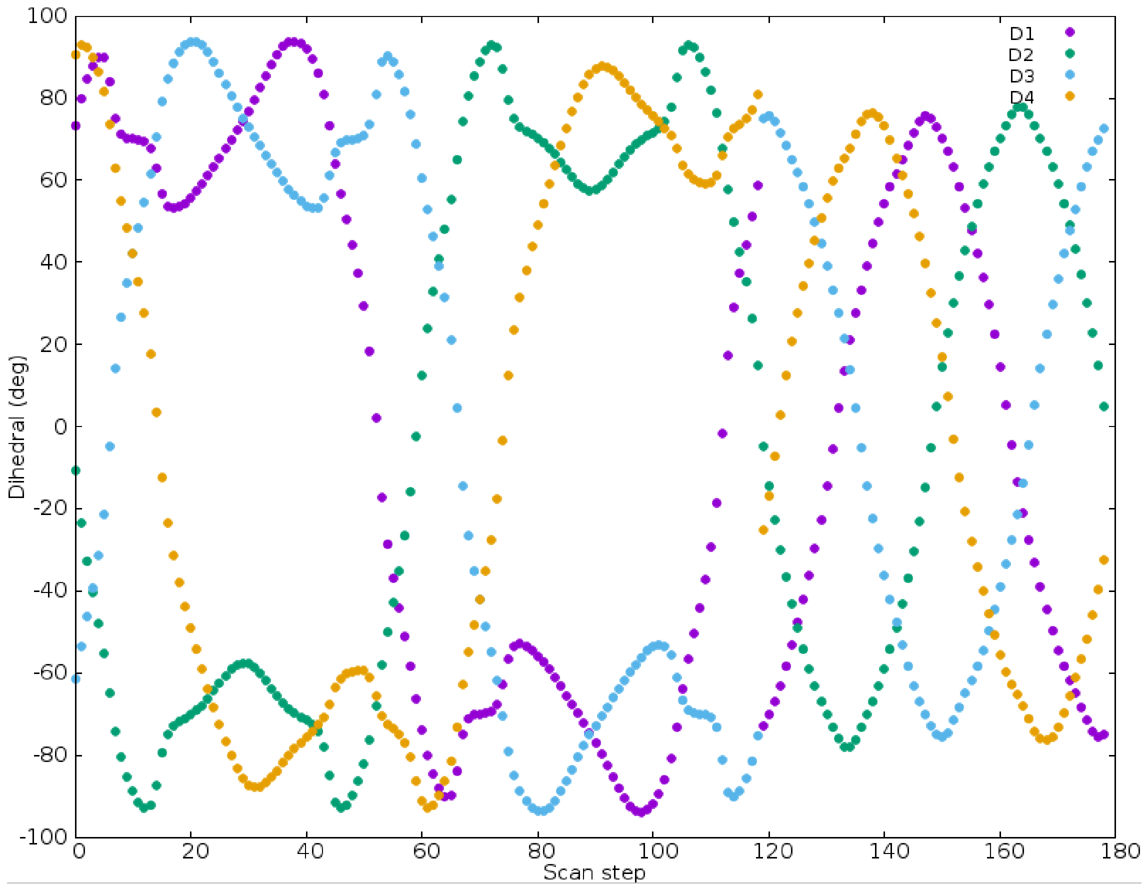

Figure S18. Evolution of the D1, D2 and D3 endocyclic dihedral angles along the scan conformational coordinate of the X=SO_2 7-membered ring ($C_6H_{10}X$) molecule.

$C_6H_{12}NH$ – TC/C plane and TB/B plane

%mem=8GB
#p opt=(modredundant,newton) b972/def2svp nosymm maxdisk=8GB scf=xqc geom=GIC

SAM dihedral scan

0 1

N	-1.845876	1.528675	0.018960
C	-2.208676	0.845024	-1.149494
C	-1.868696	0.789078	1.211181
H	-1.556776	-0.041043	-1.297788
C	-3.673092	0.417252	-1.231869
H	-1.990355	1.543141	-1.970158
C	-3.272673	0.454704	1.716388
H	-1.357312	1.421119	1.951028
H	-1.268308	-0.138223	1.108761
H	-4.301110	1.276053	-0.946723
C	-4.051910	-0.835145	-0.441131

H	-3.896643	0.222541	-2.293120
C	-3.929261	-0.783489	1.100880
H	-3.215874	0.298838	2.805510
H	-3.898187	1.349097	1.573451
H	-3.431601	-1.662359	-0.822000
H	-5.083148	-1.109120	-0.708689
H	-4.929649	-0.885471	1.547894
H	-3.372967	-1.670236	1.442436
H	-0.943110	1.831122	-0.104081

XC(inactive)=XCntr(1,2,5,11,13,7,3)
YC(inactive)=YCntr(1,2,5,11,13,7,3)
ZC(inactive)=ZCntr(1,2,5,11,13,7,3)
X1(inactive)=X(1)-XC
X2(inactive)=X(2)-XC
X3(inactive)=X(5)-XC
X4(inactive)=X(11)-XC
X5(inactive)=X(13)-XC
X6(inactive)=X(7)-XC
X7(inactive)=X(3)-XC
Y1(inactive)=Y(1)-YC
Y2(inactive)=Y(2)-YC
Y3(inactive)=Y(5)-YC
Y4(inactive)=Y(11)-YC
Y5(inactive)=Y(13)-YC
Y6(inactive)=Y(7)-YC
Y7(inactive)=Y(3)-YC
Z1(inactive)=Z(1)-ZC
Z2(inactive)=Z(2)-ZC
Z3(inactive)=Z(5)-ZC
Z4(inactive)=Z(11)-ZC
Z5(inactive)=Z(13)-ZC
Z6(inactive)=Z(7)-ZC
Z7(inactive)=Z(3)-ZC
Rpx(inactive)=0.781831482*(X2-X7)+0.974927912*(X3-X6)+0.433883739*(X4-X5)
Rpy(inactive)=0.781831482*(Y2-Y7)+0.974927912*(Y3-Y6)+0.433883739*(Y4-Y5)
Rpz(inactive)=0.781831482*(Z2-Z7)+0.974927912*(Z3-Z6)+0.433883739*(Z4-Z5)
Rppx(inactive)=X1+0.623489801*(X2+X7)-0.222520934*(X3+X6)-0.900968867*(X4+X5)
Rppy(inactive)=Y1+0.623489801*(Y2+Y7)-0.222520934*(Y3+Y6)-0.900968867*(Y4+Y5)
Rppz(inactive)=Z1+0.623489801*(Z2+Z7)-0.222520934*(Z3+Z6)-0.900968867*(Z4+Z5)
prx(inactive)=Rpy*Rppz-Rppy*Rpz
pry(inactive)=Rpz*Rppx-Rppz*Rpx
prz(inactive)=Rpx*Rppy-Rppx*Rpy
nx(inactive)=prx/SQRT(prx*prx+pry*pry+prz*prz)
ny(inactive)=pry/SQRT(prx*prx+pry*pry+prz*prz)
nz(inactive)=prz/SQRT(prx*prx+pry*pry+prz*prz)
Zt1(inactive)=X1*nx+Y1*ny+Z1*nz
Zt2(inactive)=X2*nx+Y2*ny+Z2*nz
Zt3(inactive)=X3*nx+Y3*ny+Z3*nz
Zt4(inactive)=X4*nx+Y4*ny+Z4*nz
Zt5(inactive)=X5*nx+Y5*ny+Z5*nz
Zt6(inactive)=X6*nx+Y6*ny+Z6*nz
Zt7(inactive)=X7*nx+Y7*ny+Z7*nz
PUCKB2(inactive)=SQRT(2/7)*(Zt1-0.222520934*(Zt2+Zt7)-
0.900968867*(Zt3+Zt6)+0.623489801*(Zt4+Zt5))

PUCKA2(inactive)=-1*SQRT(2/7)*(0.974927912*(Zt2-Zt7)+0.433883739*(Zt6-Zt3)+0.781831482*(Zt5-Zt4))
PUCKB3(inactive)=SQRT(2/7)*(Zt1-0.900968867*(Zt2+Zt7)+0.623489801*(Zt3+Zt6)-0.222520934*(Zt4+Zt5))
PUCKA3(inactive)=-1*SQRT(2/7)*(0.433883739*(Zt2-Zt7)+0.781831482*(Zt6-Zt3)+0.974927912*(Zt4-Zt5))
PHI3=57.29577951*(PUCKA3/(SQRT(PUCKA3**2)+0.0001))*ARCCOS(PUCKB3/SQRT(PUCKA3*PUCKA3+PUCKB3*PUCKB3))
q2=SQRT(PUCKA2*PUCKA2+PUCKB2*PUCKB2)*0.52918
q3=SQRT(PUCKA3*PUCKA3+PUCKB3*PUCKB3)*0.52918
PHI3(NSteps=118,StepSize=3.0)

%mem=8GB
#p opt=(modredundant,newton) b972/def2svp nosymm maxdisk=8GB scf=xqc geom=GIC

SAM dihedral scan

0 1

N	-2.65133600	1.68992500	-0.04737900
C	-2.59780800	0.96738600	-1.24935900
C	-1.98233500	1.13293400	1.05387100
H	-1.55343600	0.87609900	-1.60694600
C	-3.25705700	-0.41701200	-1.18515700
H	-3.13182800	1.59635600	-1.97620300
C	-2.60402300	-0.17390000	1.57779500
H	-2.04213300	1.90574500	1.83345100
H	-0.90816700	0.98563700	0.82899100
H	-3.58485000	-0.68668200	-2.20100700
C	-4.45110400	-0.46604500	-0.22938800
H	-2.52161400	-1.18479700	-0.90446500
C	-4.09789000	-0.28603200	1.27217900
H	-2.07313900	-1.04871400	1.17500600
H	-2.44841200	-0.21603200	2.66663700
H	-4.95941700	-1.43136200	-0.37085700
H	-5.18008200	0.30077900	-0.53044400
H	-4.60371500	0.60869500	1.66348100
H	-4.50376500	-1.13254500	1.84530800
H	-2.23259338	2.54002244	-0.21847960

XC(inactive)=XCntr(1,2,5,11,13,7,3)
YC(inactive)=YCntr(1,2,5,11,13,7,3)
ZC(inactive)=ZCntr(1,2,5,11,13,7,3)
X1(inactive)=X(1)-XC
X2(inactive)=X(2)-XC
X3(inactive)=X(5)-XC
X4(inactive)=X(11)-XC
X5(inactive)=X(13)-XC
X6(inactive)=X(7)-XC
X7(inactive)=X(3)-XC
Y1(inactive)=Y(1)-YC
Y2(inactive)=Y(2)-YC
Y3(inactive)=Y(5)-YC
Y4(inactive)=Y(11)-YC
Y5(inactive)=Y(13)-YC
Y6(inactive)=Y(7)-YC

Y7(inactive)=Y(3)-YC
Z1(inactive)=Z(1)-ZC
Z2(inactive)=Z(2)-ZC
Z3(inactive)=Z(5)-ZC
Z4(inactive)=Z(11)-ZC
Z5(inactive)=Z(13)-ZC
Z6(inactive)=Z(7)-ZC
Z7(inactive)=Z(3)-ZC
Rpx(inactive)=0.781831482*(X2-X7)+0.974927912*(X3-X6)+0.433883739*(X4-X5)
Rpy(inactive)=0.781831482*(Y2-Y7)+0.974927912*(Y3-Y6)+0.433883739*(Y4-Y5)
Rpz(inactive)=0.781831482*(Z2-Z7)+0.974927912*(Z3-Z6)+0.433883739*(Z4-Z5)
Rppx(inactive)=X1+0.623489801*(X2+X7)-0.222520934*(X3+X6)-0.900968867*(X4+X5)
Rppy(inactive)=Y1+0.623489801*(Y2+Y7)-0.222520934*(Y3+Y6)-0.900968867*(Y4+Y5)
Rppz(inactive)=Z1+0.623489801*(Z2+Z7)-0.222520934*(Z3+Z6)-0.900968867*(Z4+Z5)
prx(inactive)=Rpy*Rppz-Rppy*Rpz
pry(inactive)=Rpz*Rppx-Rppz*Rpx
prz(inactive)=Rpx*Rppy-Rppx*Rpy
nx(inactive)=prx/SQRT(prx*prx+pry*pry+prz*prz)
ny(inactive)=pry/SQRT(prx*prx+pry*pry+prz*prz)
nz(inactive)=prz/SQRT(prx*prx+pry*pry+prz*prz)
Zt1(inactive)=X1*nx+Y1*ny+Z1*nz
Zt2(inactive)=X2*nx+Y2*ny+Z2*nz
Zt3(inactive)=X3*nx+Y3*ny+Z3*nz
Zt4(inactive)=X4*nx+Y4*ny+Z4*nz
Zt5(inactive)=X5*nx+Y5*ny+Z5*nz
Zt6(inactive)=X6*nx+Y6*ny+Z6*nz
Zt7(inactive)=X7*nx+Y7*ny+Z7*nz
PUCKB2(inactive)=SQRT(2/7)*(Zt1-0.222520934*(Zt2+Zt7)-0.900968867*(Zt3+Zt6)+0.623489801*(Zt4+Zt5))
PUCKA2(inactive)=-1*SQRT(2/7)*(0.974927912*(Zt2-Zt7)+0.433883739*(Zt6-Zt3)+0.781831482*(Zt5-Zt4))
PUCKB3(inactive)=SQRT(2/7)*(Zt1-0.900968867*(Zt2+Zt7)+0.623489801*(Zt3+Zt6)-0.222520934*(Zt4+Zt5))
PUCKA3(inactive)=-1*SQRT(2/7)*(0.433883739*(Zt2-Zt7)+0.781831482*(Zt6-Zt3)+0.974927912*(Zt4-Zt5))
PHI2=57.29577951*(PUCKA2/(SQRT(PUCKA2**2)+0.0001))*ARCCOS(PUCKB2/SQRT(PUCKA2*PUCKA2+PUCKB2*PUCKB2))
q2=SQRT(PUCKA2*PUCKA2+PUCKB2*PUCKB2)*0.52918
q3=SQRT(PUCKA3*PUCKA3+PUCKB3*PUCKB3)*0.52918
PHI2(NSteps=59,StepSize=-6.0)

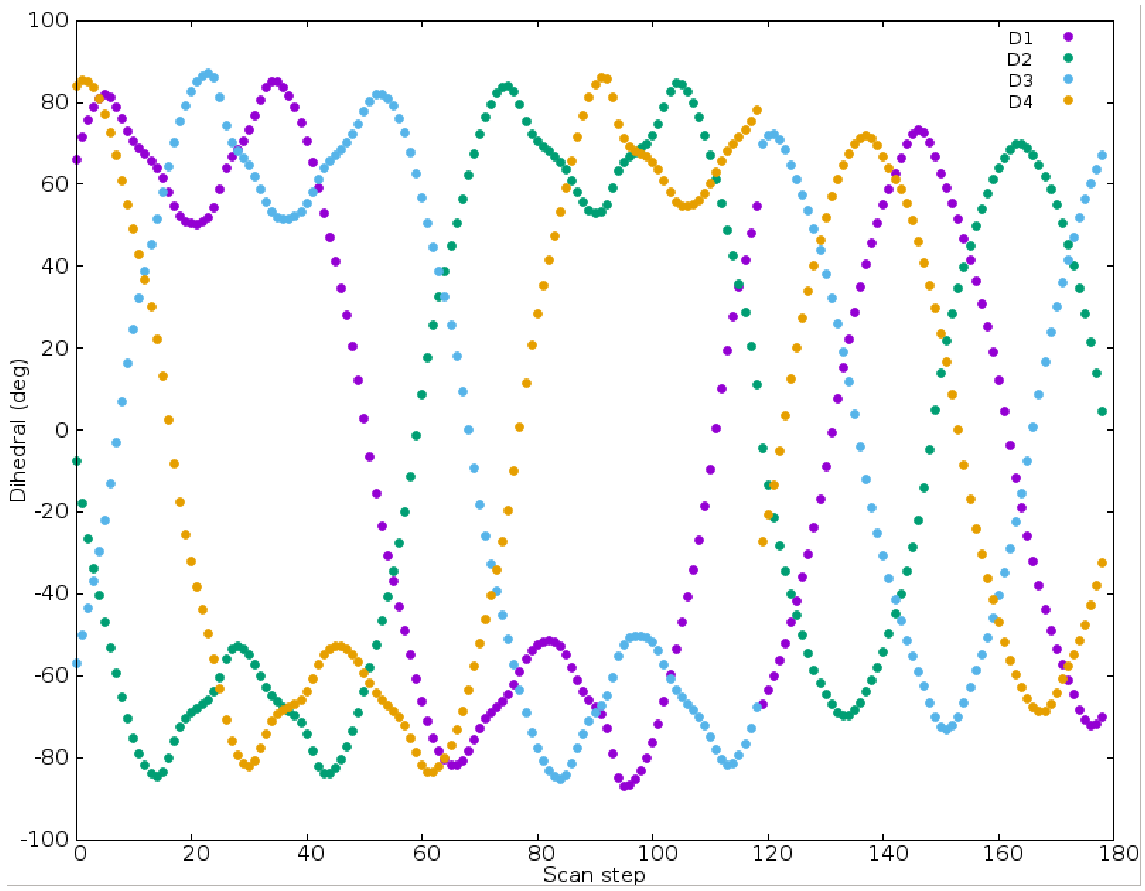

Figure S19. Evolution of the D1, D2 and D3 endocyclic dihedral angles along the scan conformational coordinate of the X=NH 7-membered ring ($C_6H_{10}X$) molecule.

$C_6H_{12}BH$ – TC/C plane and TB/B plane

%mem=8GB
#p opt=(modredundant,newton) b972/def2svp nosymm maxdisk=8GB scf=xqc geom=GIC

SAM dihedral scan

0 1

B	-1.845876	1.528675	0.018960
C	-2.208676	0.845024	-1.149494
C	-1.868696	0.789078	1.211181
H	-1.556776	-0.041043	-1.297788
C	-3.673092	0.417252	-1.231869
H	-1.990355	1.543141	-1.970158
C	-3.272673	0.454704	1.716388
H	-1.357312	1.421119	1.951028
H	-1.268308	-0.138223	1.108761
H	-4.301110	1.276053	-0.946723
C	-4.051910	-0.835145	-0.441131
H	-3.896643	0.222541	-2.293120

C	-3.929261	-0.783489	1.100880
H	-3.215874	0.298838	2.805510
H	-3.898187	1.349097	1.573451
H	-3.431601	-1.662359	-0.822000
H	-5.083148	-1.109120	-0.708689
H	-4.929649	-0.885471	1.547894
H	-3.372967	-1.670236	1.442436
H	-0.943110	1.831122	-0.104081

XC(inactive)=XCntr(1,2,5,11,13,7,3)
YC(inactive)=YCntr(1,2,5,11,13,7,3)
ZC(inactive)=ZCntr(1,2,5,11,13,7,3)
X1(inactive)=X(1)-XC
X2(inactive)=X(2)-XC
X3(inactive)=X(5)-XC
X4(inactive)=X(11)-XC
X5(inactive)=X(13)-XC
X6(inactive)=X(7)-XC
X7(inactive)=X(3)-XC
Y1(inactive)=Y(1)-YC
Y2(inactive)=Y(2)-YC
Y3(inactive)=Y(5)-YC
Y4(inactive)=Y(11)-YC
Y5(inactive)=Y(13)-YC
Y6(inactive)=Y(7)-YC
Y7(inactive)=Y(3)-YC
Z1(inactive)=Z(1)-ZC
Z2(inactive)=Z(2)-ZC
Z3(inactive)=Z(5)-ZC
Z4(inactive)=Z(11)-ZC
Z5(inactive)=Z(13)-ZC
Z6(inactive)=Z(7)-ZC
Z7(inactive)=Z(3)-ZC
Rpx(inactive)=0.781831482*(X2-X7)+0.974927912*(X3-X6)+0.433883739*(X4-X5)
Rpy(inactive)=0.781831482*(Y2-Y7)+0.974927912*(Y3-Y6)+0.433883739*(Y4-Y5)
Rpz(inactive)=0.781831482*(Z2-Z7)+0.974927912*(Z3-Z6)+0.433883739*(Z4-Z5)
Rppx(inactive)=X1+0.623489801*(X2+X7)-0.222520934*(X3+X6)-0.900968867*(X4+X5)
Rppy(inactive)=Y1+0.623489801*(Y2+Y7)-0.222520934*(Y3+Y6)-0.900968867*(Y4+Y5)
Rppz(inactive)=Z1+0.623489801*(Z2+Z7)-0.222520934*(Z3+Z6)-0.900968867*(Z4+Z5)
prx(inactive)=Rpy*Rppz-Rppy*Rpz
pry(inactive)=Rpz*Rppx-Rppz*Rpx
prz(inactive)=Rpx*Rppy-Rppx*Rpy
nx(inactive)=prx/SQRT(prx*prx+pry*pry+prz*prz)
ny(inactive)=pry/SQRT(prx*prx+pry*pry+prz*prz)
nz(inactive)=prz/SQRT(prx*prx+pry*pry+prz*prz)
Zt1(inactive)=X1*nx+Y1*ny+Z1*nz
Zt2(inactive)=X2*nx+Y2*ny+Z2*nz
Zt3(inactive)=X3*nx+Y3*ny+Z3*nz
Zt4(inactive)=X4*nx+Y4*ny+Z4*nz
Zt5(inactive)=X5*nx+Y5*ny+Z5*nz
Zt6(inactive)=X6*nx+Y6*ny+Z6*nz
Zt7(inactive)=X7*nx+Y7*ny+Z7*nz
PUCKB2(inactive)=SQRT(2/7)*(Zt1-0.222520934*(Zt2+Zt7)-
0.900968867*(Zt3+Zt6)+0.623489801*(Zt4+Zt5))

PUCKA2(inactive)=-1*SQRT(2/7)*(0.974927912*(Zt2-Zt7)+0.433883739*(Zt6-Zt3)+0.781831482*(Zt5-Zt4))
PUCKB3(inactive)=SQRT(2/7)*(Zt1-0.900968867*(Zt2+Zt7)+0.623489801*(Zt3+Zt6)-0.222520934*(Zt4+Zt5))
PUCKA3(inactive)=-1*SQRT(2/7)*(0.433883739*(Zt2-Zt7)+0.781831482*(Zt6-Zt3)+0.974927912*(Zt4-Zt5))
PHI3=57.29577951*(PUCKA3/(SQRT(PUCKA3**2)+0.0001))*ARCCOS(PUCKB3/SQRT(PUCKA3*PUCKA3+PUCKB3*PUCKB3))
q2(frozen)=SQRT(PUCKA2*PUCKA2+PUCKB2*PUCKB2)*0.52918
q3=SQRT(PUCKA3*PUCKA3+PUCKB3*PUCKB3)*0.52918
PHI3(NSteps=118,StepSize=3.0)

%mem=8GB
#p opt=(modredundant,newton) b972/def2svp nosymm maxdisk=8GB scf=xqc geom=GIC

SAM dihedral scan

0 1

B	-2.65133600	1.68992500	-0.04737900
C	-2.59780800	0.96738600	-1.24935900
C	-1.98233500	1.13293400	1.05387100
H	-1.55343600	0.87609900	-1.60694600
C	-3.25705700	-0.41701200	-1.18515700
H	-3.13182800	1.59635600	-1.97620300
C	-2.60402300	-0.17390000	1.57779500
H	-2.04213300	1.90574500	1.83345100
H	-0.90816700	0.98563700	0.82899100
H	-3.58485000	-0.68668200	-2.20100700
C	-4.45110400	-0.46604500	-0.22938800
H	-2.52161400	-1.18479700	-0.90446500
C	-4.09789000	-0.28603200	1.27217900
H	-2.07313900	-1.04871400	1.17500600
H	-2.44841200	-0.21603200	2.66663700
H	-4.95941700	-1.43136200	-0.37085700
H	-5.18008200	0.30077900	-0.53044400
H	-4.60371500	0.60869500	1.66348100
H	-4.50376500	-1.13254500	1.84530800
H	-2.23259338	2.54002244	-0.21847960

XC(inactive)=XCntr(1,2,5,11,13,7,3)
YC(inactive)=YCntr(1,2,5,11,13,7,3)
ZC(inactive)=ZCntr(1,2,5,11,13,7,3)
X1(inactive)=X(1)-XC
X2(inactive)=X(2)-XC
X3(inactive)=X(5)-XC
X4(inactive)=X(11)-XC
X5(inactive)=X(13)-XC
X6(inactive)=X(7)-XC
X7(inactive)=X(3)-XC
Y1(inactive)=Y(1)-YC
Y2(inactive)=Y(2)-YC
Y3(inactive)=Y(5)-YC
Y4(inactive)=Y(11)-YC
Y5(inactive)=Y(13)-YC
Y6(inactive)=Y(7)-YC

```
Y7(inactive)=Y(3)-YC
Z1(inactive)=Z(1)-ZC
Z2(inactive)=Z(2)-ZC
Z3(inactive)=Z(5)-ZC
Z4(inactive)=Z(11)-ZC
Z5(inactive)=Z(13)-ZC
Z6(inactive)=Z(7)-ZC
Z7(inactive)=Z(3)-ZC
Rpx(inactive)=0.781831482*(X2-X7)+0.974927912*(X3-X6)+0.433883739*(X4-X5)
Rpy(inactive)=0.781831482*(Y2-Y7)+0.974927912*(Y3-Y6)+0.433883739*(Y4-Y5)
Rpz(inactive)=0.781831482*(Z2-Z7)+0.974927912*(Z3-Z6)+0.433883739*(Z4-Z5)
Rppx(inactive)=X1+0.623489801*(X2+X7)-0.222520934*(X3+X6)-0.900968867*(X4+X5)
Rppy(inactive)=Y1+0.623489801*(Y2+Y7)-0.222520934*(Y3+Y6)-0.900968867*(Y4+Y5)
Rppz(inactive)=Z1+0.623489801*(Z2+Z7)-0.222520934*(Z3+Z6)-0.900968867*(Z4+Z5)
prx(inactive)=Rpy*Rppz-Rppy*Rpz
pry(inactive)=Rpz*Rppx-Rppz*Rpx
prz(inactive)=Rpx*Rppy-Rppx*Rpy
nx(inactive)=prx/SQRT(prx*prx+pry*pry+prz*prz)
ny(inactive)=pry/SQRT(prx*prx+pry*pry+prz*prz)
nz(inactive)=prz/SQRT(prx*prx+pry*pry+prz*prz)
Zt1(inactive)=X1*nx+Y1*ny+Z1*nz
Zt2(inactive)=X2*nx+Y2*ny+Z2*nz
Zt3(inactive)=X3*nx+Y3*ny+Z3*nz
Zt4(inactive)=X4*nx+Y4*ny+Z4*nz
Zt5(inactive)=X5*nx+Y5*ny+Z5*nz
Zt6(inactive)=X6*nx+Y6*ny+Z6*nz
Zt7(inactive)=X7*nx+Y7*ny+Z7*nz
PUCKB2(inactive)=SQRT(2/7)*(Zt1-0.222520934*(Zt2+Zt7)-
0.900968867*(Zt3+Zt6)+0.623489801*(Zt4+Zt5))
PUCKA2(inactive)=-1*SQRT(2/7)*(0.974927912*(Zt2-Zt7)+0.433883739*(Zt6-Zt3)+0.781831482*(Zt5-
Zt4))
PUCKB3(inactive)=SQRT(2/7)*(Zt1-0.900968867*(Zt2+Zt7)+0.623489801*(Zt3+Zt6)-
0.222520934*(Zt4+Zt5))
PUCKA3(inactive)=-1*SQRT(2/7)*(0.433883739*(Zt2-Zt7)+0.781831482*(Zt6-Zt3)+0.974927912*(Zt4-
Zt5))
PHI2=57.29577951*(PUCKA2/(SQRT(PUCKA2**2)+0.0001))*ARCCOS(PUCKB2/SQRT(PUCKA2*PUC
KA2+PUCKB2*PUCKB2))
q2(frozen)=SQRT(PUCKA2*PUCKA2+PUCKB2*PUCKB2)*0.52918
q3=SQRT(PUCKA3*PUCKA3+PUCKB3*PUCKB3)*0.52918
PHI2(NSteps=59,StepSize=-6.0)
```

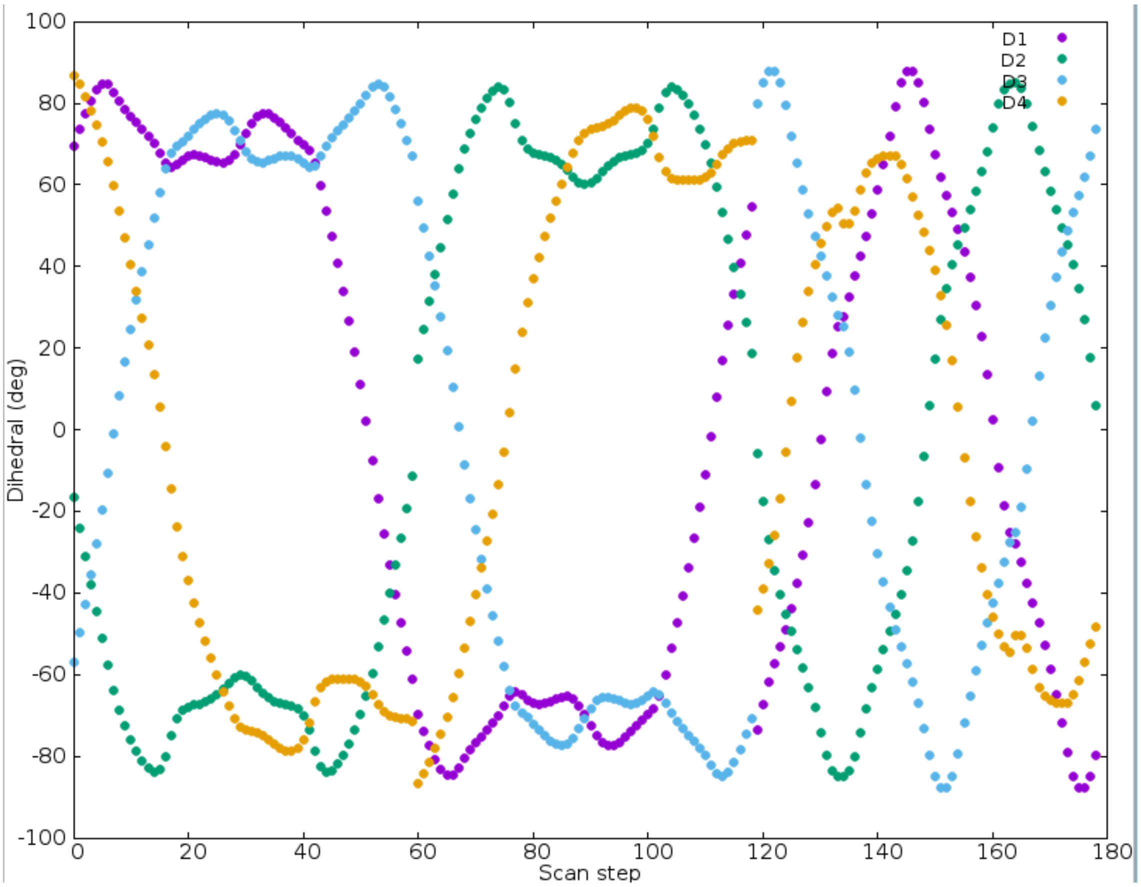

Figure S20. Evolution of the D1, D2 and D3 endocyclic dihedral angles along the scan conformational coordinate of the X=BH 7-membered ring ($C_6H_{10}X$) molecule.

C_6H_{12}PH – TC/C plane and TB/B plane

%mem=8GB
#p opt=(modredundant,newton) b972/def2svp nosymm maxdisk=8GB scf=xqc geom=GIC

SAM dihedral scan

0 1

P	-1.845876	1.528675	0.018960
C	-2.208676	0.845024	-1.149494
C	-1.868696	0.789078	1.211181
H	-1.556776	-0.041043	-1.297788
C	-3.673092	0.417252	-1.231869
H	-1.990355	1.543141	-1.970158
C	-3.272673	0.454704	1.716388
H	-1.357312	1.421119	1.951028
H	-1.268308	-0.138223	1.108761
H	-4.301110	1.276053	-0.946723
C	-4.051910	-0.835145	-0.441131
H	-3.896643	0.222541	-2.293120
C	-3.929261	-0.783489	1.100880

H	-3.215874	0.298838	2.805510
H	-3.898187	1.349097	1.573451
H	-3.431601	-1.662359	-0.822000
H	-5.083148	-1.109120	-0.708689
H	-4.929649	-0.885471	1.547894
H	-3.372967	-1.670236	1.442436
H	-0.943110	1.831122	-0.104081

```
XC(inactive)=XCntr(1,2,5,11,13,7,3)
YC(inactive)=YCntr(1,2,5,11,13,7,3)
ZC(inactive)=ZCntr(1,2,5,11,13,7,3)
X1(inactive)=X(1)-XC
X2(inactive)=X(2)-XC
X3(inactive)=X(5)-XC
X4(inactive)=X(11)-XC
X5(inactive)=X(13)-XC
X6(inactive)=X(7)-XC
X7(inactive)=X(3)-XC
Y1(inactive)=Y(1)-YC
Y2(inactive)=Y(2)-YC
Y3(inactive)=Y(5)-YC
Y4(inactive)=Y(11)-YC
Y5(inactive)=Y(13)-YC
Y6(inactive)=Y(7)-YC
Y7(inactive)=Y(3)-YC
Z1(inactive)=Z(1)-ZC
Z2(inactive)=Z(2)-ZC
Z3(inactive)=Z(5)-ZC
Z4(inactive)=Z(11)-ZC
Z5(inactive)=Z(13)-ZC
Z6(inactive)=Z(7)-ZC
Z7(inactive)=Z(3)-ZC
Rpx(inactive)=0.781831482*(X2-X7)+0.974927912*(X3-X6)+0.433883739*(X4-X5)
Rpy(inactive)=0.781831482*(Y2-Y7)+0.974927912*(Y3-Y6)+0.433883739*(Y4-Y5)
Rpz(inactive)=0.781831482*(Z2-Z7)+0.974927912*(Z3-Z6)+0.433883739*(Z4-Z5)
Rppx(inactive)=X1+0.623489801*(X2+X7)-0.222520934*(X3+X6)-0.900968867*(X4+X5)
Rppy(inactive)=Y1+0.623489801*(Y2+Y7)-0.222520934*(Y3+Y6)-0.900968867*(Y4+Y5)
Rppz(inactive)=Z1+0.623489801*(Z2+Z7)-0.222520934*(Z3+Z6)-0.900968867*(Z4+Z5)
prx(inactive)=Rpy*Rppz-Rppy*Rpz
pry(inactive)=Rpz*Rppx-Rppz*Rpx
prz(inactive)=Rpx*Rppy-Rppx*Rpy
nx(inactive)=prx/SQRT(prx*prx+pry*pry+prz*prz)
ny(inactive)=pry/SQRT(prx*prx+pry*pry+prz*prz)
nz(inactive)=prz/SQRT(prx*prx+pry*pry+prz*prz)
Zt1(inactive)=X1*nx+Y1*ny+Z1*nz
Zt2(inactive)=X2*nx+Y2*ny+Z2*nz
Zt3(inactive)=X3*nx+Y3*ny+Z3*nz
Zt4(inactive)=X4*nx+Y4*ny+Z4*nz
Zt5(inactive)=X5*nx+Y5*ny+Z5*nz
Zt6(inactive)=X6*nx+Y6*ny+Z6*nz
Zt7(inactive)=X7*nx+Y7*ny+Z7*nz
PUCKB2(inactive)=SQRT(2/7)*(Zt1-0.222520934*(Zt2+Zt7)-
0.900968867*(Zt3+Zt6)+0.623489801*(Zt4+Zt5))
PUCKA2(inactive)=-1*SQRT(2/7)*(0.974927912*(Zt2-Zt7)+0.433883739*(Zt6-Zt3)+0.781831482*(Zt5-
Zt4))
```

PUCKB3(inactive)=SQRT(2/7)*(Zt1-0.900968867*(Zt2+Zt7)+0.623489801*(Zt3+Zt6)-0.222520934*(Zt4+Zt5))
PUCKA3(inactive)=-1*SQRT(2/7)*(0.433883739*(Zt2-Zt7)+0.781831482*(Zt6-Zt3)+0.974927912*(Zt4-Zt5))
PHI3=57.29577951*(PUCKA3/(SQRT(PUCKA3**2)+0.0001))*ARCCOS(PUCKB3/SQRT(PUCKA3*PUCKA3+PUCKB3*PUCKB3))
q2=SQRT(PUCKA2*PUCKA2+PUCKB2*PUCKB2)*0.52918
q3=SQRT(PUCKA3*PUCKA3+PUCKB3*PUCKB3)*0.52918
PHI3(NSteps=118,StepSize=3.0)

%mem=8GB
#p opt=(modredundant,newton) b972/def2svp nosymm maxdisk=8GB scf=xqc geom=GIC

SAM dihedral scan

0 1

P	-2.65133600	1.68992500	-0.04737900
C	-2.59780800	0.96738600	-1.24935900
C	-1.98233500	1.13293400	1.05387100
H	-1.55343600	0.87609900	-1.60694600
C	-3.25705700	-0.41701200	-1.18515700
H	-3.13182800	1.59635600	-1.97620300
C	-2.60402300	-0.17390000	1.57779500
H	-2.04213300	1.90574500	1.83345100
H	-0.90816700	0.98563700	0.82899100
H	-3.58485000	-0.68668200	-2.20100700
C	-4.45110400	-0.46604500	-0.22938800
H	-2.52161400	-1.18479700	-0.90446500
C	-4.09789000	-0.28603200	1.27217900
H	-2.07313900	-1.04871400	1.17500600
H	-2.44841200	-0.21603200	2.66663700
H	-4.95941700	-1.43136200	-0.37085700
H	-5.18008200	0.30077900	-0.53044400
H	-4.60371500	0.60869500	1.66348100
H	-4.50376500	-1.13254500	1.84530800
H	-2.23259338	2.54002244	-0.21847960

XC(inactive)=XCntr(1,2,5,11,13,7,3)
YC(inactive)=YCntr(1,2,5,11,13,7,3)
ZC(inactive)=ZCntr(1,2,5,11,13,7,3)
X1(inactive)=X(1)-XC
X2(inactive)=X(2)-XC
X3(inactive)=X(5)-XC
X4(inactive)=X(11)-XC
X5(inactive)=X(13)-XC
X6(inactive)=X(7)-XC
X7(inactive)=X(3)-XC
Y1(inactive)=Y(1)-YC
Y2(inactive)=Y(2)-YC
Y3(inactive)=Y(5)-YC
Y4(inactive)=Y(11)-YC
Y5(inactive)=Y(13)-YC
Y6(inactive)=Y(7)-YC
Y7(inactive)=Y(3)-YC
Z1(inactive)=Z(1)-ZC

```
Z2(inactive)=Z(2)-ZC
Z3(inactive)=Z(5)-ZC
Z4(inactive)=Z(11)-ZC
Z5(inactive)=Z(13)-ZC
Z6(inactive)=Z(7)-ZC
Z7(inactive)=Z(3)-ZC
Rpx(inactive)=0.781831482*(X2-X7)+0.974927912*(X3-X6)+0.433883739*(X4-X5)
Rpy(inactive)=0.781831482*(Y2-Y7)+0.974927912*(Y3-Y6)+0.433883739*(Y4-Y5)
Rpz(inactive)=0.781831482*(Z2-Z7)+0.974927912*(Z3-Z6)+0.433883739*(Z4-Z5)
Rppx(inactive)=X1+0.623489801*(X2+X7)-0.222520934*(X3+X6)-0.900968867*(X4+X5)
Rppy(inactive)=Y1+0.623489801*(Y2+Y7)-0.222520934*(Y3+Y6)-0.900968867*(Y4+Y5)
Rppz(inactive)=Z1+0.623489801*(Z2+Z7)-0.222520934*(Z3+Z6)-0.900968867*(Z4+Z5)
prx(inactive)=Rpy*Rppz-Rppy*Rpz
pry(inactive)=Rpz*Rppx-Rppz*Rpx
prz(inactive)=Rpx*Rppy-Rppx*Rpy
nx(inactive)=prx/SQRT(prx*prx+pry*pry+prz*prz)
ny(inactive)=pry/SQRT(prx*prx+pry*pry+prz*prz)
nz(inactive)=prz/SQRT(prx*prx+pry*pry+prz*prz)
Zt1(inactive)=X1*nx+Y1*ny+Z1*nz
Zt2(inactive)=X2*nx+Y2*ny+Z2*nz
Zt3(inactive)=X3*nx+Y3*ny+Z3*nz
Zt4(inactive)=X4*nx+Y4*ny+Z4*nz
Zt5(inactive)=X5*nx+Y5*ny+Z5*nz
Zt6(inactive)=X6*nx+Y6*ny+Z6*nz
Zt7(inactive)=X7*nx+Y7*ny+Z7*nz
PUCKB2(inactive)=SQRT(2/7)*(Zt1-0.222520934*(Zt2+Zt7)-
0.900968867*(Zt3+Zt6)+0.623489801*(Zt4+Zt5))
PUCKA2(inactive)=-1*SQRT(2/7)*(0.974927912*(Zt2-Zt7)+0.433883739*(Zt6-Zt3)+0.781831482*(Zt5-
Zt4))
PUCKB3(inactive)=SQRT(2/7)*(Zt1-0.900968867*(Zt2+Zt7)+0.623489801*(Zt3+Zt6)-
0.222520934*(Zt4+Zt5))
PUCKA3(inactive)=-1*SQRT(2/7)*(0.433883739*(Zt2-Zt7)+0.781831482*(Zt6-Zt3)+0.974927912*(Zt4-
Zt5))
PHI2=57.29577951*(PUCKA2/(SQRT(PUCKA2**2)+0.0001))*ARCCOS(PUCKB2/SQRT(PUCKA2*PUC
KA2+PUCKB2*PUCKB2))
q2=SQRT(PUCKA2*PUCKA2+PUCKB2*PUCKB2)*0.52918
q3=SQRT(PUCKA3*PUCKA3+PUCKB3*PUCKB3)*0.52918
PHI2(NSteps=59,StepSize=-6.0)
```

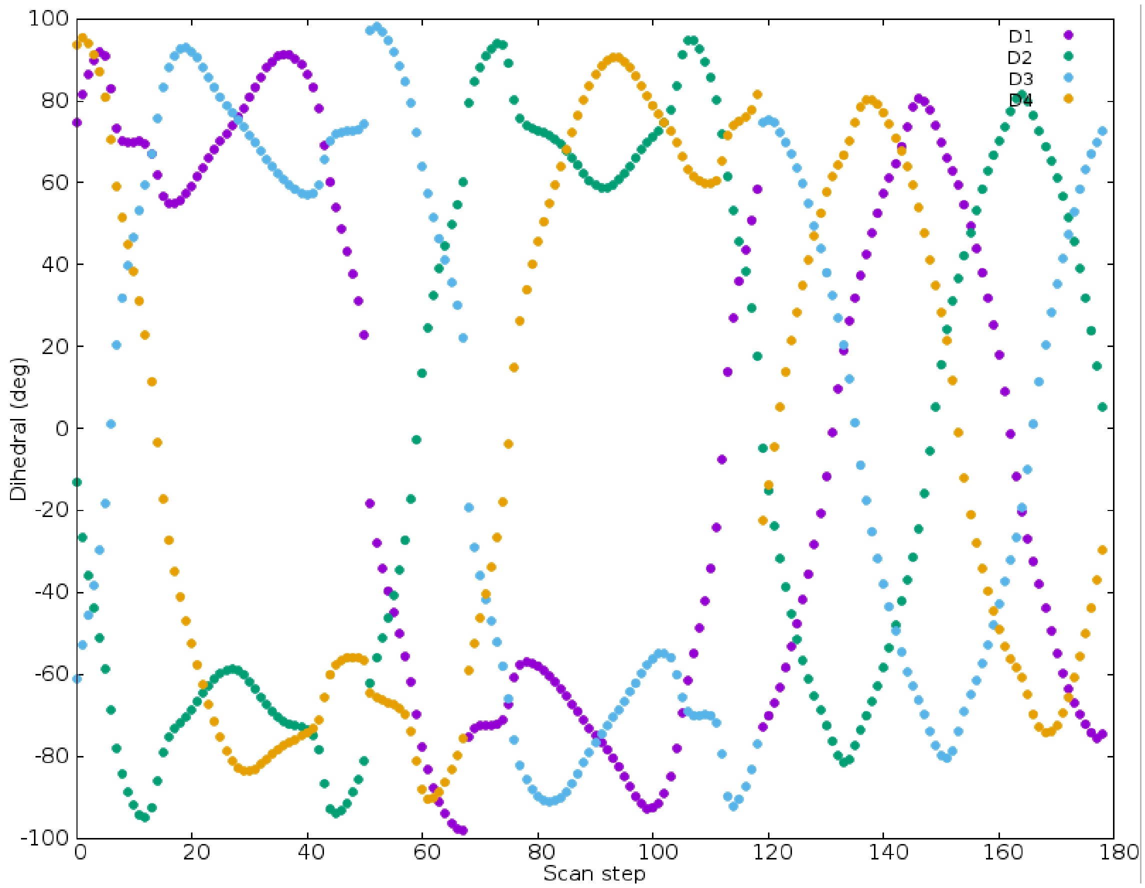

Figure S21. Evolution of the D1, D2 and D3 endocyclic dihedral angles along the scan conformational coordinate of the X=PH 7-membered ring ($C_6H_{10}X$) molecule.

List of available functional groups in *MonteCarbo*

Table S1. Order, code, name, number of rotamers and structure of the functional groups available in MonteCarbo for the construction of the multifunctionalized saccharides.

#	Code	Name	# Rotamers	Structure
1	H (default)	Hydrogen	0	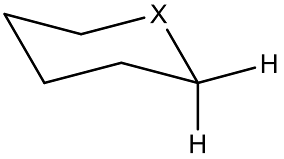
2	F	Fluor	0	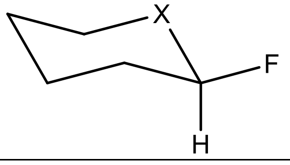
3	Cl	Chloride	0	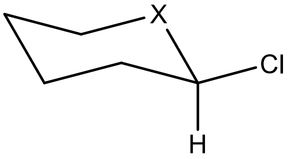
4	Br	Bromide	0	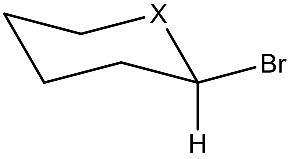
5	CH3	Methyl	0	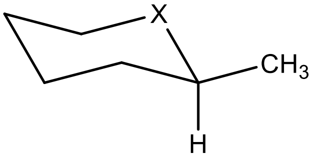
6	C<br>O	Endocyclic carbonyl	0	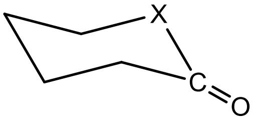
7	NH2	Amino	1	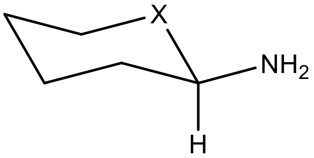
8	OH	Alcohol	1	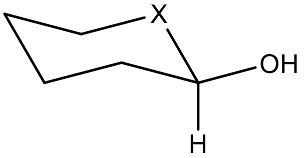
9	SH	Thiol	1	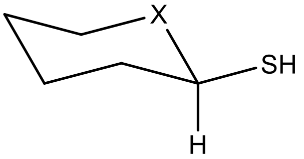
10	NO2	Nitro	1	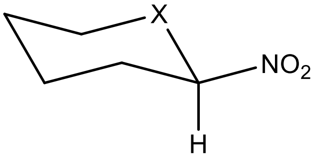

11	CH2OH	Methyl-alcohol	2	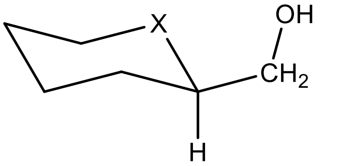
12	CH2OCH3	Methyl methyl ether	2	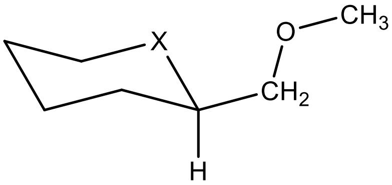
13	CO2H	Carboxylic acid	1	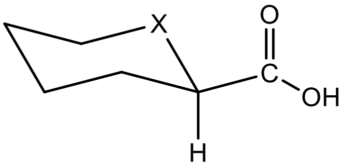
14	Bz	Benzyl	1	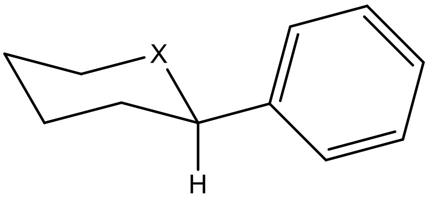
15	NAc	N-Acetyl	1	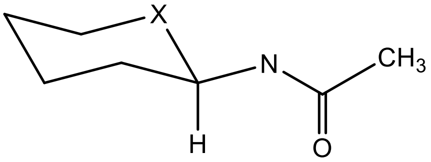
16	pNP	Para-nitro-phenyl (-O-Bz-NO2)	1	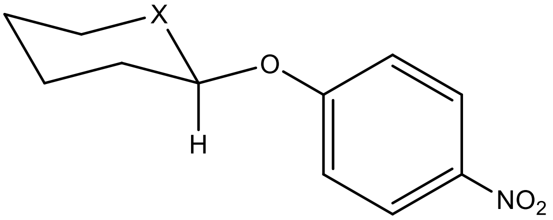
17	OCH3	Methoxy	1	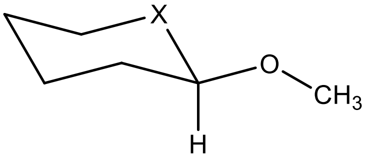
18	OAc	Acetyl	2	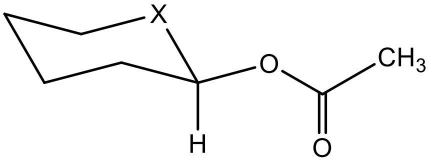
19	SCH3	Methyl-thiol	1	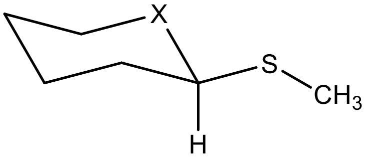
20	H2PO4	Dihydrogenphosphate	1	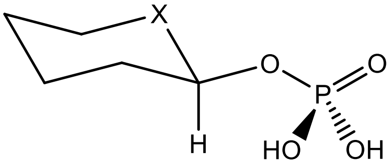

21	HSO4	Hydrogensulphate	1	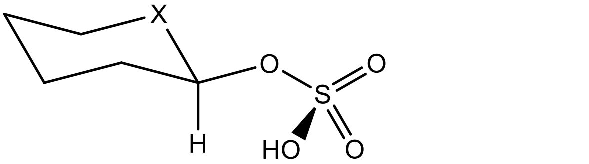
22	Ade	Adenine	1	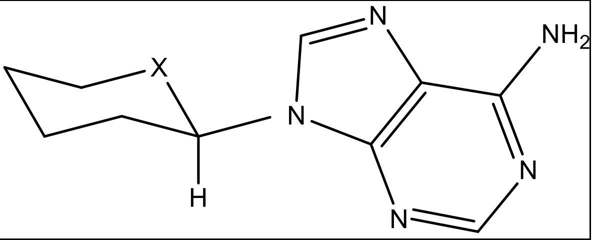
23	Thy	Thymine	1	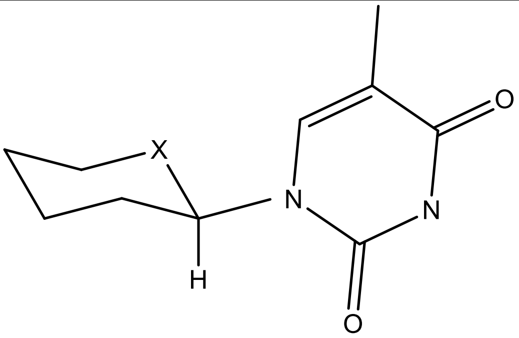
24	Ura	Uracyl	1	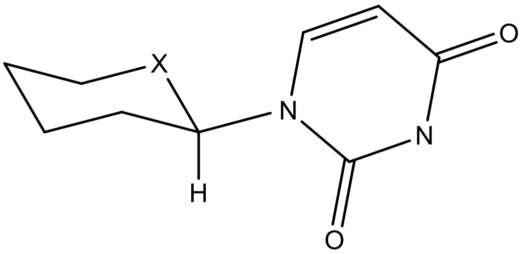
25	Cyt	Cytosine	1	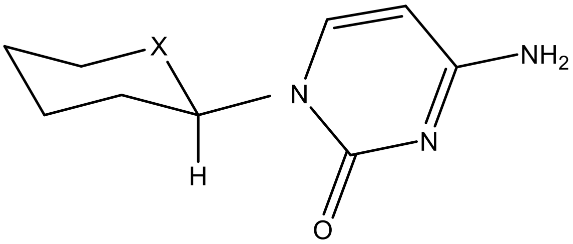
26	Gua	Guanine	1	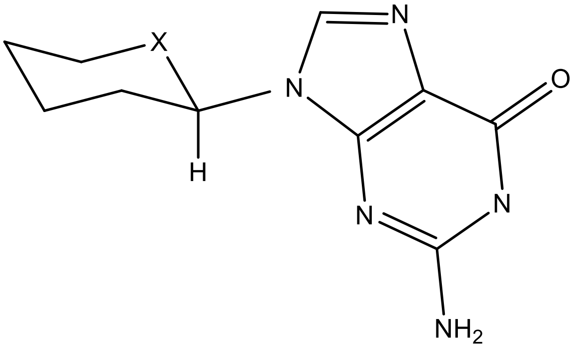

Applications

Simple case: 2-hydroxy-tetrahydrofurane

MonteCarbo input (Figure 8)

test5
NOPUCKPDB
5
1
Oxy
H
H
H
H
H
OH
H
H
H
500

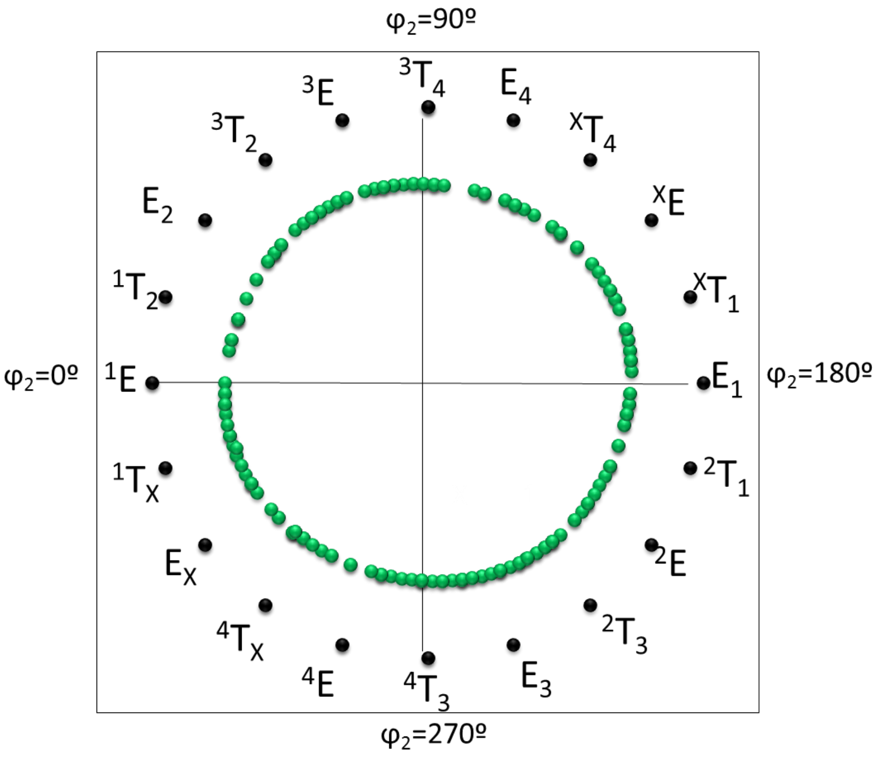

Figure S22. Puckering coordinates of the 500 5-membered ring structures generated by *MonteCarbo* (Figure 8).

Test case: α-D-glucose

MonteCarbo input (Figure 9)

test6
NOPUCKPDB
6
1
Oxy
H
OH
H
OH
OH
H
H
OH
CH2OH
H
500

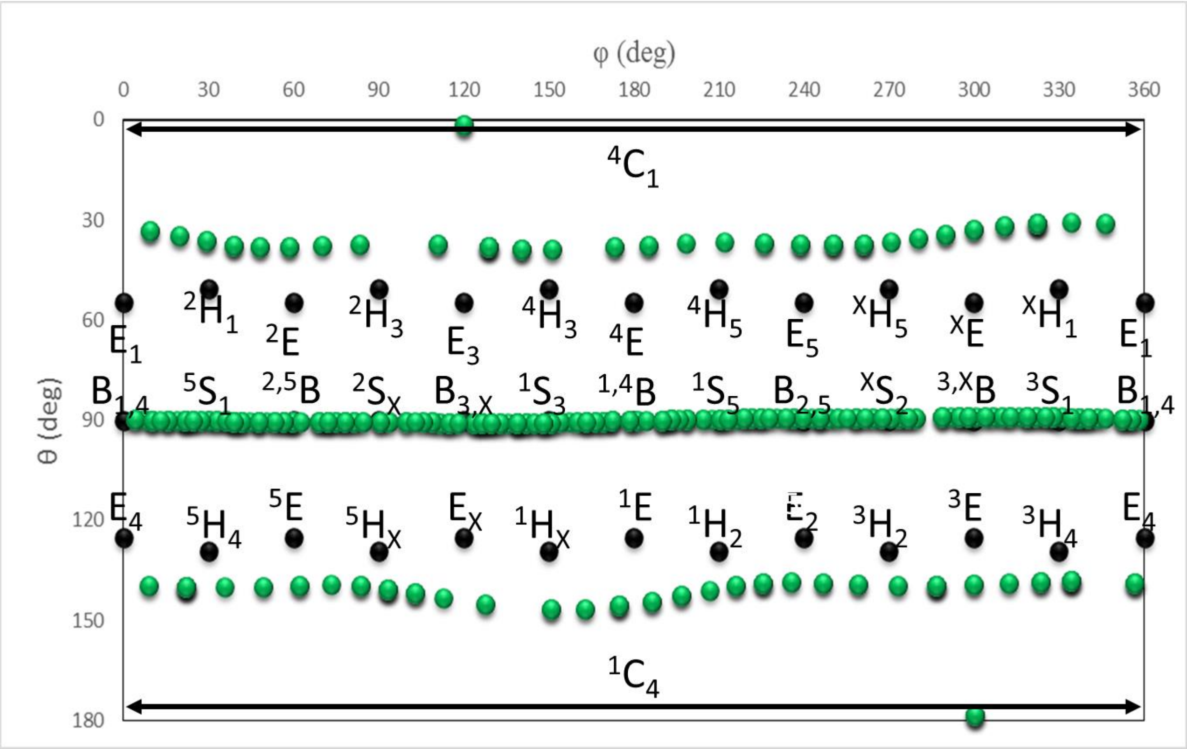

Figure S23. Puckering coordinates of the 500 6-membered ring structures generated by *MonteCarbo* (Figure 9).

Docking: a 7-membered ring mimics mannose

MonteCarbo input (Generation of 500 structures of α-D-glycero-D-idoseptanoside)

testAIDOSEPTA
NOPUCKPDB
7
1
Oxy
H
OH
OH
H
H
OH
OH
H
H
OH
CH2OH
H
500

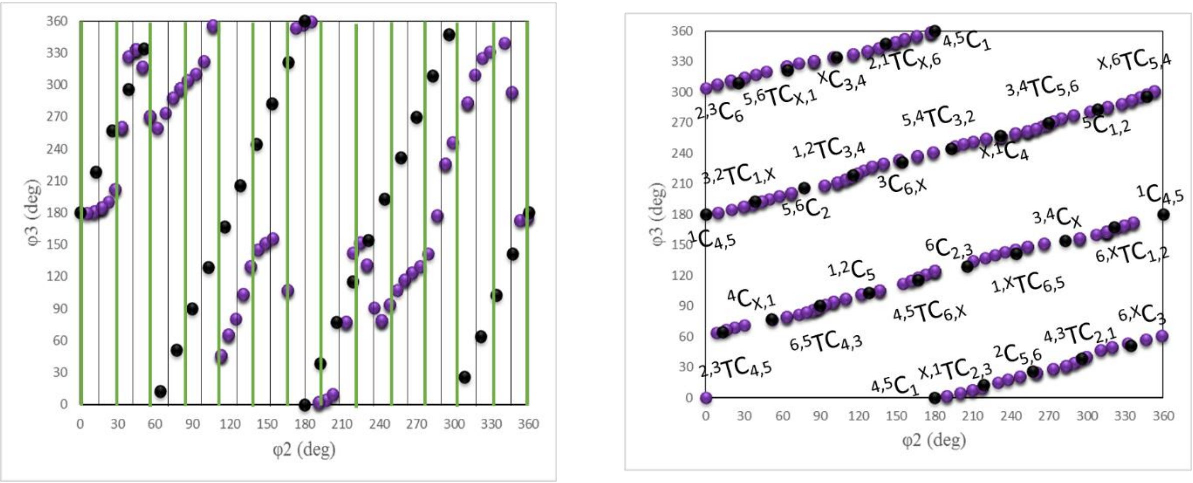

Figure S24. Puckering coordinates for the 500 6-membered ring structures generated by *MonteCarbo*.

MonteCarbo input (Generation and optimization of 50 structures of α-*D*-glycero-*D*-idoseptanoside)

testAIDOSEPTA
PUCK
7
1
Oxy
H
OH
OH
H
H
OH
OH
H
H
OH
CH2OH
H
50

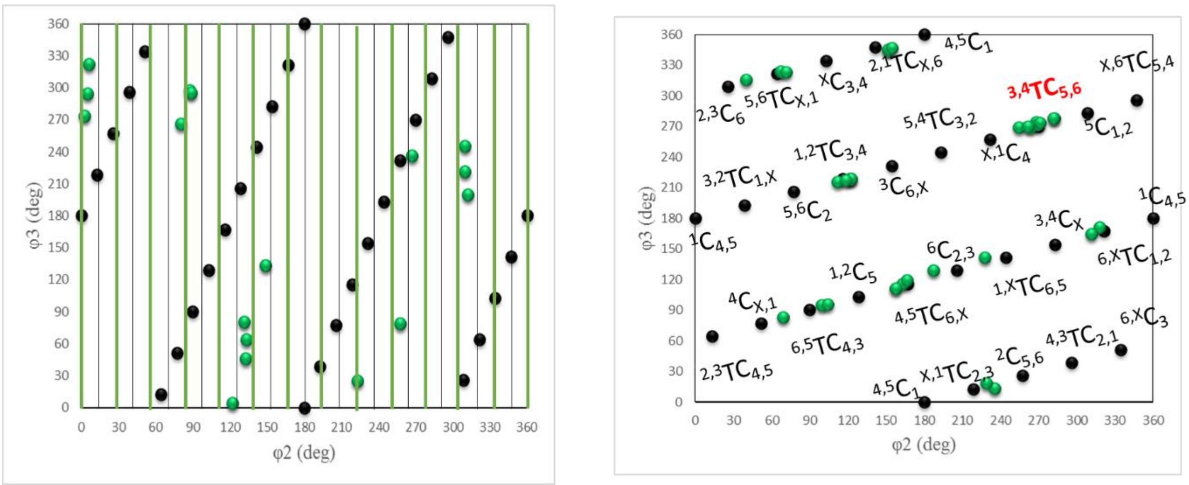

Figure S25. Puckering coordinates for the 50 6-membered ring optimized structures generated by *MonteCarbo*.

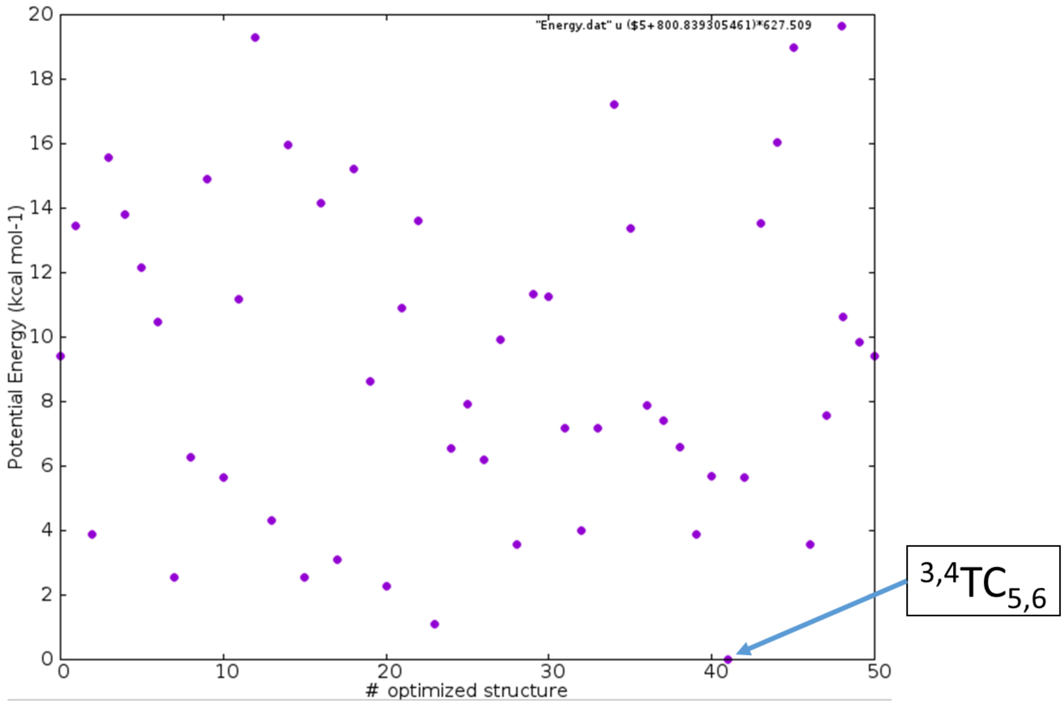

Figure S26. Energetics of the 50 6-membered ring optimized structures generated by *MonteCarbo*. The $^{3,4}TC_{5,6}$ conformer is the structure with the lowest potential energy.

Optimization of the $^{3,4}TC_{5,6}$ structure of hydrolyzed α-D-glycerol-D-idoseptanoside, 1-methyl-α-D-glycerol-D-idoseptanoside and dimethyl-1,X-α-D-glycerol-D-idoseptanoside, for X=2, 3, 4, 5 and 7

α-D-glycerol-D-idoseptanoside

28
calc7.gjf.out Energy: -502522.8852669

C	0.09013	-0.08718	-0.10375
C	-0.03434	0.01346	1.42487
C	1.33266	0.04380	2.13590
C	1.99457	1.43240	2.20231
C	2.48893	1.98948	0.86271
C	1.40788	1.92189	-0.22347
O	1.22499	0.57625	-0.59765
H	0.26179	-1.14226	-0.35416
O	-1.07801	0.29114	-0.74833
H	-1.48226	1.02367	-0.27575
O	-0.79414	-1.08033	1.84590
H	-0.66005	-1.12614	2.80033
H	-0.55985	0.96167	1.67600
H	2.01937	-0.66014	1.64098
O	1.11412	-0.37283	3.48733

H	1.63871	-1.17729	3.65047
O	3.10104	1.37011	3.05839
H	2.76550	1.03809	3.89550
H	1.23704	2.14296	2.59458
H	3.31430	1.33362	0.53391
O	3.00628	3.28177	1.02759
H	2.40136	3.90819	0.61203
C	1.74898	2.73961	-1.46398
O	1.49733	4.09686	-1.15191
H	1.89440	4.65571	-1.82913
H	2.80091	2.57358	-1.75168
H	1.10606	2.38346	-2.28531
H	0.46910	2.36303	0.15935

1-methyl-α-D-glycerol-D-idoseptanoside

31
calc7-1CH3.gjf.out Energy: -527164.2964828

C	0.13801	-0.25755	0.18958
C	0.14710	-0.11184	1.70833
C	1.56472	0.00579	2.26821
C	2.10779	1.44046	2.24257
C	2.32803	2.11183	0.88384
C	1.20741	1.88858	-0.14955
O	1.09078	0.51121	-0.47429
H	0.41260	-1.29946	-0.05233
O	-1.17073	0.00310	-0.22268
O	-0.44876	-1.25685	2.28376
H	-1.38363	-1.24352	2.06016
H	-0.42808	0.78857	1.98625
H	2.24352	-0.65503	1.69653
O	1.56974	-0.35561	3.63018
H	0.92399	-1.06560	3.72763
O	3.33893	1.46600	2.91128
H	3.19813	0.99176	3.73808
H	1.36203	2.06514	2.77378
H	3.25132	1.66914	0.45789
O	2.48568	3.47700	1.15974
H	2.42788	3.95570	0.32298
C	1.50139	2.54504	-1.49932
O	1.64463	3.94189	-1.35724
H	1.96754	4.30890	-2.18332
H	2.41642	2.07998	-1.90703
H	0.66989	2.29751	-2.18060
H	0.25756	2.29429	0.23628
C	-1.48535	-0.43023	-1.52318
H	-2.52819	-0.15374	-1.71824
H	-0.84270	0.04527	-2.27973
H	-1.38359	-1.52541	-1.61943

dimethyl-1,2-α-D-glycerol-D-idoseptanoside

34
calc-CH3-2-12-34TC56.gjf.out Energy: -551798.7690993

C	0.14321	-0.25695	0.08100
C	0.10304	-0.14791	1.60680
C	1.50896	0.01059	2.20447
C	2.00624	1.46190	2.22504
C	2.28226	2.14515	0.88387
C	1.23345	1.88105	-0.21143
O	1.15860	0.49561	-0.51323
H	0.39747	-1.30086	-0.17213
O	-1.12313	0.06702	-0.40382
O	-0.45867	-1.32813	2.14691
H	-0.51438	0.71972	1.89606
H	2.22393	-0.61439	1.63756
O	1.48511	-0.38216	3.55659
H	0.89430	-1.14529	3.59225
O	3.19785	1.51905	2.96040
H	3.03417	1.01392	3.76443
H	1.21423	2.06041	2.71853
H	3.24549	1.73639	0.51664
O	2.37282	3.51540	1.16494
H	2.36981	3.98705	0.32222
C	1.59931	2.52112	-1.55128
O	1.72512	3.92095	-1.42050
H	2.09915	4.27841	-2.22895
H	2.53987	2.05770	-1.89822
H	0.81112	2.25919	-2.27721
H	0.25502	2.27499	0.10940
C	-1.37664	-0.34446	-1.72483
H	-2.38697	-0.00766	-1.98497
H	-0.66011	0.09414	-2.43603
H	-1.33286	-1.44340	-1.81976
C	-1.84188	-1.29664	2.40566
H	-2.42512	-1.11707	1.49087
H	-2.11446	-2.27523	2.81995
H	-2.09680	-0.51700	3.14446

dimethyl-1,3-α-D-glycerol-D-idoseptanoside

34
calc-CH3-2-13-34TC56.gjf.out Energy: -551795.9667318

C	-0.05241	-0.16484	0.14552
C	-0.15338	0.04834	1.65601
C	1.23555	0.04921	2.30881
C	1.89272	1.44366	2.27246
C	2.24486	2.05755	0.91336
C	1.19311	1.87638	-0.19570
O	1.00042	0.49941	-0.47826

H	0.15679	-1.23501	-0.03121
O	-1.30386	0.17010	-0.37942
O	-0.95825	-0.97004	2.19394
H	-1.81711	-0.88790	1.76775
H	-0.62429	1.03095	1.84076
H	1.89556	-0.67828	1.80280
O	1.16261	-0.25649	3.68433
O	3.08217	1.39814	3.00931
H	2.83971	0.96765	3.83744
H	1.16974	2.14074	2.74095
H	3.16484	1.54222	0.56941
O	2.47471	3.41750	1.16371
H	2.51199	3.87105	0.31177
C	1.63740	2.45733	-1.53860
O	1.87180	3.84489	-1.42939
H	2.28238	4.15789	-2.23858
H	2.54431	1.91379	-1.85788
H	0.84425	2.24405	-2.27498
H	0.24984	2.36434	0.09959
C	-1.55557	-0.30636	-1.67831
H	-2.55615	0.03717	-1.96631
H	-0.82347	0.07920	-2.40425
H	-1.53392	-1.40965	-1.71520
C	1.29912	-1.61221	4.02963
H	0.49661	-2.22727	3.60083
H	2.27593	-2.01437	3.70464
H	1.24576	-1.67112	5.12424

dimethyl-1,4-α-D-glycerol-D-idoseptanoside

34

calc-CH3-2-14-34TC56.gjf.out Energy: -551796.8119236

C	0.05613	-0.32719	0.06400
C	0.04518	-0.29354	1.58811
C	1.44912	-0.31151	2.20651
C	2.08835	1.08117	2.27209
C	2.30115	1.87589	0.97374
C	1.17393	1.81084	-0.07064
O	1.01896	0.47906	-0.53488
H	0.31796	-1.35177	-0.25358
O	-1.24739	-0.01909	-0.33912
O	-0.63875	-1.43894	2.05357
H	-1.55453	-1.36224	1.77059
H	-0.48394	0.61658	1.92231
H	2.10162	-0.98049	1.61271
O	1.36063	-0.76091	3.53339
H	0.67021	-1.43427	3.53950
O	3.35665	0.96086	2.86817
H	1.42384	1.69699	2.90271
H	3.20067	1.44400	0.49134

O	2.52518	3.19651	1.39581
H	2.49515	3.76556	0.61577
C	1.49351	2.59557	-1.34355
O	1.69669	3.96145	-1.04998
H	2.01844	4.40585	-1.83762
H	2.38778	2.13953	-1.80405
H	0.65153	2.45906	-2.04304
H	0.23642	2.20492	0.35512
C	-1.55333	-0.34702	-1.67189
H	-2.59067	-0.04305	-1.85549
H	-0.89681	0.17739	-2.38294
H	-1.46371	-1.43272	-1.85171
C	3.48385	1.47255	4.16600
H	3.28136	2.55691	4.19666
H	2.82003	0.95754	4.87839
H	4.52421	1.30694	4.47587

dimethyl-1,5-α-D-glycerol-D-idoseptanoside

34
calc-CH3-2-15-34TC56.gjf.out Energy: -551802.9380175

C	0.14035	-0.39203	0.12171
C	0.10466	-0.07590	1.61679
C	1.47644	0.30033	2.17601
C	1.86095	1.77157	1.95973
C	2.06477	2.22226	0.51157
C	0.90222	1.83601	-0.41839
O	0.96954	0.43363	-0.63188
H	0.57052	-1.40198	-0.00304
O	-1.19042	-0.38154	-0.29941
O	-0.33132	-1.22077	2.32031
H	-1.24417	-1.38661	2.06711
H	-0.59850	0.75736	1.78871
H	2.24884	-0.34493	1.71570
O	1.47826	0.13573	3.57535
H	0.93232	-0.63827	3.76085
O	3.04987	2.02245	2.66402
H	2.93114	1.62074	3.53306
H	1.03208	2.38243	2.37050
H	2.97088	1.72926	0.11512
O	2.21937	3.62801	0.45236
C	0.97587	2.52941	-1.78175
O	0.59945	3.87084	-1.72287
H	1.13456	4.25264	-1.01436
H	1.98973	2.38425	-2.20614
H	0.27572	2.01086	-2.45163
H	-0.06446	2.12484	0.02477
C	-1.42420	-0.96518	-1.55871
H	-2.49742	-0.87960	-1.76472
H	-0.86526	-0.45203	-2.35524

H	-1.14315	-2.03290	-1.56610
C	3.52474	4.13918	0.61241
H	4.22503	3.67783	-0.10521
H	3.47213	5.21573	0.40682
H	3.90197	3.97661	1.62957

dimethyl-1,7-α-D-glycerol-D-idoseptanoside

34
calc7-2CH3.gjf.out   Energy: -551801.0001891

C	0.16636	-0.22187	0.17872
C	0.14005	-0.10725	1.69988
C	1.54510	-0.02357	2.29638
C	2.10984	1.40288	2.31424
C	2.37316	2.09992	0.97622
C	1.27692	1.91348	-0.08993
O	1.14736	0.54460	-0.44539
H	0.43052	-1.26293	-0.07796
O	-1.12743	0.06839	-0.26020
O	-0.48747	-1.25401	2.23684
H	-1.41604	-1.22168	1.99015
H	-0.42781	0.79644	1.98223
H	2.22768	-0.68293	1.72757
O	1.51122	-0.41301	3.65019
H	0.85283	-1.11487	3.71704
O	3.32444	1.39603	3.01299
H	3.15667	0.90706	3.82604
H	1.36037	2.02729	2.84054
H	3.30239	1.65636	0.56500
O	2.53708	3.45713	1.28556
H	2.52199	3.95091	0.45576
C	1.61731	2.58824	-1.41980
O	1.81276	3.96915	-1.25905
H	2.52405	2.10645	-1.83237
H	0.79147	2.39417	-2.12772
H	0.32382	2.32637	0.27919
C	-1.41622	-0.33375	-1.57670
H	-2.44920	-0.03601	-1.79193
H	-0.74703	0.14569	-2.30729
H	-1.32999	-1.42836	-1.69244
C	2.14788	4.64475	-2.43969
H	1.35214	4.54771	-3.19916
H	2.27094	5.70590	-2.19314
H	3.09031	4.26790	-2.87535

Docking using *MCdock*

Table S2. PDB code, glycoside hydrolase family (GH), stereochemistry of the anomeric carbon, endo/exo activity, retaining/inverting activity, presence of a metal in the active site, expected conformation of the natural substrate and anomeric configuration for the 9 mannanase/mannosidase active enzymes available in the library of *MCdock*.

PDB	GH	α/β	Endo/exo	Ret./Inv.	Metal	Conf. MC	Anomeric config.
2vx6	26	β	Endo	Ret.	-	$^1S_3$	-1,4-
**2wzs**	92	α	Exo	Inv.	Ca2+	**"$^OS_2$"**	-1,(2,3,4,6)-
**3d4y**	38	α	Exo	Ret.	Zn2+	**$^OS_2$**	-1,(3,6)-
4ayp	47	α	Exo	Inv.	Ca2+	$^3S_1$	-1,2-
**5agd**	76	α	Endo	Ret.	-	**$^OS_2$**	-1,6-
5b0s	130	β	Exo	Inv.	PO43-	$^1S_5$	-1,2- (phosphorylase)
5jug	134	β	Endo	Inv.	-	$^1C_4$	-1,4-
**5m7i**	125	α	Exo	Inv.	-	**$^OS_2$**	-1,6-
6fwg	99	α	Endo	Ret.	-	$^2E/^2H_3$	-1,2- (epoxid int.)

After performing the docking calculations of all the septanosides in the active site of the 9 mannanase/mannosidase enzymes of **Table S2**, the binding energies of the best enzyme-septanoside complexes are summarize in **Table S3**.

Table S2. Summary of the binding energies of the best match between septanosides and mannanase/mannosidase enzymes.

	Hydrolized	1-CH_3	-1,2-	-1,3-	-1,4-	-1,5-	-1,7-
2vx6	-5.9	-5.8	-5.5	-5.7	-5.1	-5.6	-5.2
2wzs	-5.8	-6.2	-4.6	-4.3	-3.6	-4.2	-5.3
3d4y	-7.4*	-6.3	-5.1	-3.5	-3.2	-5.0	-6.1
4ayp	-4.5	-4.1	-3.3	-3.3	-2.7	-2.7	-2.8
5agd	-5.9	-5.3	-5.1	-4.7	-5.2	-5.3	-5.6
5b0s	-5.4	-5.3	-4.4	-5.0	-5.2	-4.6	-4.9
5jug	-6.0	-6.1	-5.6	-5.3	-5.2	-5.4	-5.9
5m7i	-5.8	-5.8	-2.8	-3.3	-4.4	-4.2	-4.8
6fwg	-5.7	-5.3	-4.6	-5.0	-5.5	-4.6	-5.0

*The orientation of the sugar is slightly different from the natural substrate. However, the 8th match of the docking calculation (-5.0 kcal mol^{-1}) presents a perfect mimicry with the 6-membered ring.

The 5m7i-(1-CH_3) complex presented in **Figure 10** results in a binding energy of -5.8 kcal mol^{-1}.

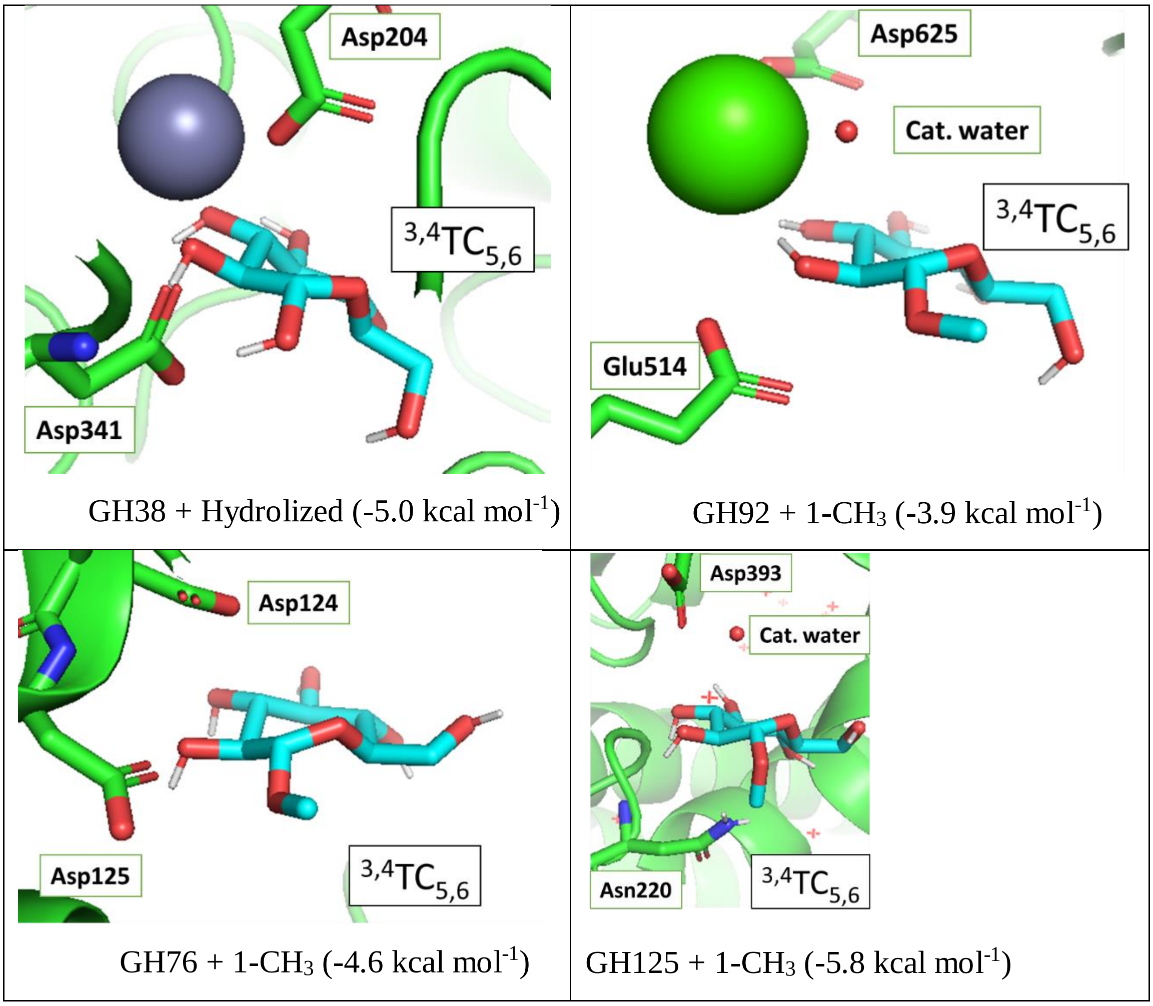

GH38 + Hydrolized (-5.0 kcal mol^{-1})

GH92 + 1-CH_3 (-3.9 kcal mol^{-1})

GH76 + 1-CH_3 (-4.6 kcal mol^{-1})

GH125 + 1-CH_3 (-5.8 kcal mol^{-1})

Figure S27. Glycoside hydrolase-septanoside structures obtained by docking using *AutoDock Vina* in combination with *MCdock*.
